# Design and Optimization of Quinazoline Derivatives: New Non-nucleoside Inhibitors of Bovine Viral Diarrhea Virus

**DOI:** 10.3389/fchem.2020.590235

**Published:** 2020-12-10

**Authors:** Gabriela A. Fernández, Eliana F. Castro, Rocío A. Rosas, Daniela M. Fidalgo, Natalia S. Adler, Leandro Battini, Maria J. España de Marco, Matias Fabiani, Ana M. Bruno, Mariela Bollini, Lucia V. Cavallaro

**Affiliations:** ^1^Laboratorio de Química Medicinal, Centro de Investigaciones en Bionanociencias (CIBION)-Consejo Nacional de Investigaciones Científicas y Técnicas (CONICET), Buenos Aires, Argentina; ^2^Centro de Investigaciones en Ciencias Veterinarias y Agronómicas, Instituto de Virología e Innovaciones Tecnológicas, Instituto Nacional de Tecnología Agropecuaria, Consejo Nacional de Investigaciones Científicas y Técnicas (CONICET), Buenos Aires, Argentina; ^3^Departamento de Microbiología, Inmunología, Biotecnología y Genética, Cátedra Virología, Facultad de Farmacia y Bioquímica, Universidad de Buenos Aires, Buenos Aires, Argentina; ^4^Departamento de Microbiología, Inmunología, Biotecnología y Genética, Cátedra Virología, Facultad de Farmacia y Bioquímica, Instituto de Investigaciones en Bacteriología y Virología Molecular (IBaViM), Universidad de Buenos Aires, Buenos Aires, Argentina; ^5^Consejo Nacional de Investigaciones Científicas y Técnicas (CONICET), Buenos Aires, Argentina; ^6^Departamento de Química Orgánica, Facultad de Farmacia y Bioquímica, Universidad de Buenos Aires, Buenos Aires, Argentina

**Keywords:** quinazoline derivatives, BVDV inhibitors, RdRp protein, pharmacokinetics *in vitro* properties, molecular dynamics

## Abstract

Bovine viral diarrhea virus (BVDV) belongs to the *Pestivirus* genus (*Flaviviridae*). In spite of the availability of vaccines, the virus is still causing substantial financial losses to the livestock industry. In this context, the use of antiviral agents could be an alternative strategy to control and reduce viral infections. The viral RNA-dependent RNA polymerase (RdRp) is essential for the replication of the viral genome and constitutes an attractive target for the identification of antiviral compounds. In a previous work, we have identified potential molecules that dock into an allosteric binding pocket of BVDV RdRp via a structure-based virtual screening approach. One of them, *N*-(2-morpholinoethyl)-2-phenylquinazolin-4-amine [**1**, 50% effective concentration (EC_50_) = 9.7 ± 0.5 μM], was selected to perform different chemical modifications. Among 24 derivatives synthesized, eight of them showed considerable antiviral activity. Molecular modeling of the most active compounds showed that they bind to a pocket located in the fingers and thumb domains in BVDV RdRp, which is different from that identified for other non-nucleoside inhibitors (NNIs) such as thiosemicarbazone (TSC). We selected compound 2-[4-(2-phenylquinazolin-4-yl)piperazin-1-yl]ethanol (**1.9**; EC_50_ = 1.7 ± 0.4 μM) for further analysis. Compound **1.9** was found to inhibit the *in vitro* replication of TSC-resistant BVDV variants, which carry the N264D mutation in the RdRp. In addition, **1.9** presented adequate solubility in different media and a high-stability profile in murine and bovine plasma.

## Introduction

Bovine viral diarrhea virus 1 (BVDV) (*Pestivirus* A) belongs to the *Pestivirus* genus of the *Flaviviridae* family (Smith et al., 2017). BVDV is distributed worldwide and is endemic in many countries (Baker, 1995; Chimeno Zoth and Taboga, 2006). BVDV infections can manifest as generalized immunosuppression, fertility disorders, and other signs such as fever, diarrhea, and respiratory dysfunction (Fray et al., 2000; Fulton et al., 2003; Beaudeau et al., 2005). Thus, this disease leads to considerable financial losses within the livestock industry (Houe, 2003).

The control of BVDV infections combines vaccination (which is not mandatory in some countries) with the removal of persistently infected animals, which are the main source of infective virus in the field (Hessman et al., 2009). The period taken to develop a protective immune response after vaccination provides a window for viral transmission. The use of antiviral agents could be an attractive strategy as a stopgap measure to prevent infection in naïve calves (Newcomer et al., 2014). Moreover, antivirals could also be used to treat valuable animals infected with pestiviruses in zoological collections and in in-breeding programs and *in vitro* embryo production, to cure established cell lines contaminated with pestiviruses, which are used, for example, to produce interferons and vaccines for medical use (Stringfellow et al., 2005; Newcomer and Givens, 2013; Newcomer et al., 2014; Musiu et al., 2020). However, no antivirals are currently available for controlling BVDV infections in either laboratories or farms. Over the last decades, several selective anti-BVDV compounds have been reported, including virus targeting and host targeting derivatives, e.g., polymerase inhibitors (Castro et al., 2019), protease inhibitors (Bukhtiyarova et al., 2001), human cellular enzyme inhibitors (Branza-Nichita et al., 2001), and entry inhibitors (Pascual et al., 2018). Furthermore, the design of effective antiviral therapies will likely require a combination of drugs with complementary mechanisms of action to decrease the probability of selecting resistant viral mutants (Newcomer and Givens, 2013). In the host cell, the viral replication is carried out by a large membrane-bound replication complex composed of viral RNA, viral polymerase, and other viral and cellular proteins. In this replication complex, a negative-strand RNA is synthesized from a positive-strand RNA genome, resulting in dsRNA intermediates from which numerous positive strands are produced for progeny viruses (Choi et al., 2006). The viral RNA-dependent RNA polymerase (NS5B RdRp) is responsible of viral RNA synthesis and has been used as a target of several nucleoside inhibitors (NIs) and non-nucleoside inhibitors (NNIs). NIs are widely used to treat hepatitis B virus (HBV), hepatitis C virus (HCV), HIV-1, and herpesvirus infections. They act by competing with nucleotide substrates for binding to the active site of the polymerase and induce termination of the nucleic acid chain synthesis (Finkielsztein et al., 2010). In turn, NNIs are highly specific and act allosterically by inhibiting the enzymatic activity of the polymerase. This group includes a series of very diverse chemical structures, such as *N*-propyl-*N*-[2-(2*H*-1,2,4-triazino[5,6-b]indol-3-ylthio)ethyl]-1-propanamine (VP32947) (Baginski et al., 2000), bromophenyl-imidazo-pyridines (BPIP) (Paeshuyse et al., 2007), ethyl 2-methylimidazo[1,2-a]pyrrolo[2,3-c]pyridin-8-carboxylate (AG110) (Paeshuyse et al., 2007), pyrazolo-triazolo-pyrimidinamine (LZ37) (Paeshuyse et al., 2009), 2-(2-benzimidazolyl)-5-[4-(2-imidazolino)phenyl]furan (DB772) (Newcomer et al., 2012), 5,6-dimethoxy-1-indanone [thiosemicarbazone (TSC)] (Castro et al., 2011; Soraires Santacruz et al., 2017), 2-phenylbenzimidazole (Tonelli et al., 2010), and arylazoenamine derivatives (Giliberti et al., 2010).

**Figure 1:**
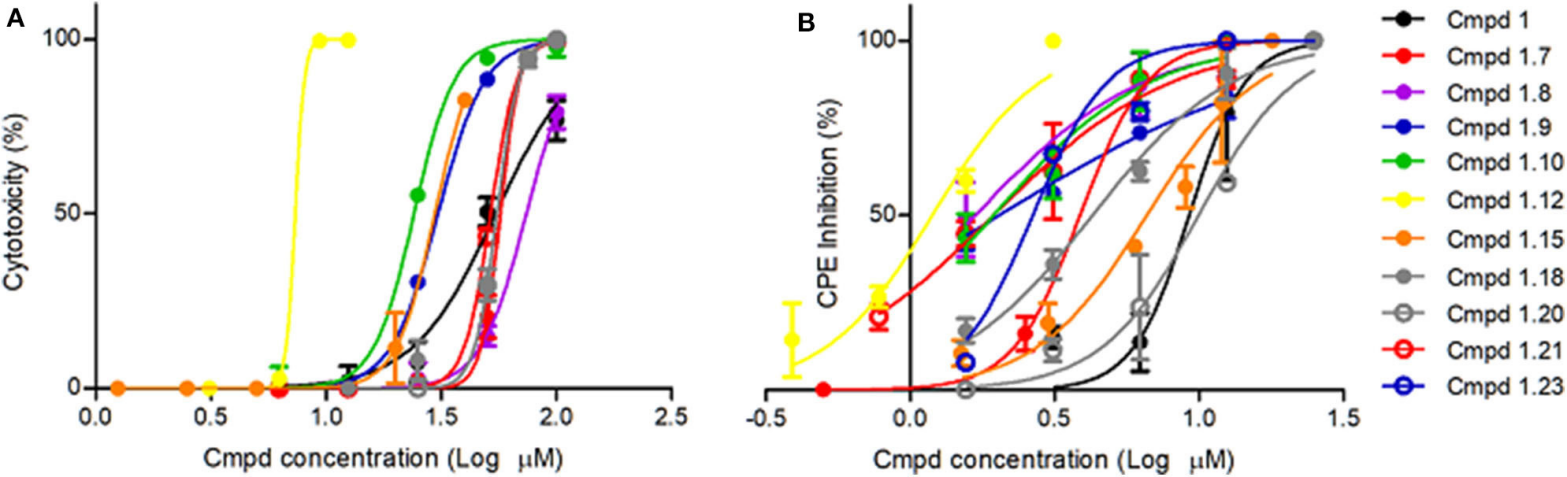
Cytotoxicity and anti-BVDV activity of active compound **1** derivatives. **(A)** Cytotoxicity. Sub-confluent MDBK cell monolayers grown in 96-well plates were treated with 2-fold serial dilutions of each compound in IM. Mock-treated cells were added as control. Cells were incubated and allowed to proliferate for 3 days at 37°C and 5% CO_2_. Cell viability was then determined by the MTS/PMS method. **(B)** Antiviral activity. Sub-confluent MDBK cell monolayers grown in 96-well plates were infected with BVDV NADL (MOI = 0.01) and treated with different concentrations of the compounds. Mock-infected cells and mock-treated infected cells were included as controls in each plate. After 3 days at 37°C and 5% CO_2_, cell viability was determined by the MTS/PMS method, and the percentage of the viral CPE inhibition was calculated.

In a previous work, we identified a series of molecules that dock into the allosteric binding pocket of BVDV RdRp, employing a structure-based virtual screening approach. Five of them were found to be active against BVDV *in vitro* and displayed 50% effective concentration (EC_50_) values in the sub- and low-micromolar range (Castro et al., 2019). From these series of structurally and functionally diverse compounds, Compound **1** (EC_50_ = 9.7 ± 0.5 μM) was chosen for further optimization as it resulted an easily synthesizable and scalable compound (Figure 1).

Compound **1** bears a quinazoline scaffold which is associated with different pharmacological activities such as analgesic, anti-inflammatory, anticonvulsant, sedative-hypnotic, antihistaminic, antihypertensive, anticancer, antimicrobial, antitubercular, and antiviral (Alagarsamy et al., 2018). Different quinazoline derivatives were reported to show antiviral activity against different human viruses such as human cytomegalovirus (Fröhlich et al., 2017; Gerna et al., 2019), influenza A virus (Wang et al., 2020), human immunodeficiency virus (Machara et al., 2016), and vaccinia virus and adenovirus (Kang et al., 2016). Interestingly, a quinazolinone derivative was reported as an NNI of dengue virus 2 RdRp, a virus which belongs to the same family as BVDV (Yao et al., 2018).

The present work reports the synthesis and anti-BVDV activity of a new family of heterocyclic compounds derived from quinazolines. With the aim of characterizing their mode of action, we combined molecular dynamics (MD) simulation and the assessment of the antiviral activity against well-characterized BVDV mutant viruses that are resistant to NNIs of the viral RdRp. In addition, the compound cytotoxicity and *in vitro* pharmacokinetic properties were determined. Together, our results suggest that these molecules are suitable antivirals for BVDV and can be used as a good starting point to optimize antiviral potency.

## Materials and Methods

### Chemistry

#### General Information

All reagents and solvents were obtained from commercial sources. Column chromatography was carried out by employing a silica gel (Kieselgel 60, 63–200 m, Merck). Pre-coated F-254 silica gel plates were used for thin-layer analytical chromatography. ^1^H and ^13^C NMR spectra were recorded on Bruker BioSpin 600 MHz AVIII-600, Bruker Avance II 500 MHz, and Bruker 300 MHz spectrometers at room temperature. Chemical shifts (δ) are reported in ppm and coupling constants (*J*) in hertz. The mass spectrometer utilized was a Xevo G2-S QTOF (Waters Corporation, Manchester, UK) with an electrospray ionization (ESI) source. The mass spectrometer was operated in positive and negative ion modes with probe capillary voltages of 2.5 and 2.3 kV, respectively. The sampling cone voltage was 30 V. The source and desolvation gas temperatures were set to 120 and 350°C, respectively. The nitrogen gas desolvation flow rate was 600 L h^−1^, and the cone desolvation flow rate was 10 L h^−1^. The mass spectrometer was calibrated across the range of *m*/*z* 50–1,200 using a 0.5 mM sodium formate solution prepared in 2-propanol/water (90:10 v/v). Data were drift-corrected during acquisition using a leucine enkephalin reference spray (LockSpray) infused at 2 μl min^−1^. Data were acquired in the range of *m*/*z* 50–1,200, and the scan time was set from 22 to 1 s. Data acquisition and processing were carried out using MassLynx, version 4.1 (Waters Corporation, Milford, MA, USA). UV spectra were measured with a Shimadzu 3,600 UV/Vis/NIR spectrophotometer.

The synthesis of intermediate **4a**–**4g** and **5a**–**5g** are described in the Supplementary Material.

#### Synthesis of 4-Substituted 2-Phenylquinazoline Derivatives 1.6–1.24

Compounds **1**–**1.5** were synthesized according to Bollini et al. (2019).

4-Chloro-2-phenylquinazoline (**5a**–**5f**) and 4-chloro-quinazoline (**5g**) compounds (0.21 mmol) and *n*-butanol (1 ml) were placed in a sealed vial. *N,N*-Di-isopropylethylamine (DIPEA) (5 eq.), and the corresponding amine (2.8 eq.) was added. The mixture was stirred at room temperature, and the reaction progress was monitored by thin-layer chromatography (TLC). The mixture was extracted with AcOEt and washed with brine, and the organic phase was dried over anhydrous Na_2_SO_4_. The products were obtained after purification by column chromatography with dichloromethane:methanol (99:1 to 90:10) mixtures (Storz et al., 2011; Duan et al., 2016; Bollini et al., 2019).

##### N-Hexyl-2-phenylquinazolin-4-amine (1.6)

Yellow solid (41 mg, 0.16 mmol, 78%). ^1^H NMR (600 MHz, CDCl_3_) δ 8.60–8.54 (m, 2H), 7.97 (d, *J* = 8.4 Hz, 1H), 7.74 (d, *J* = 8.2 Hz, 1H), 7.70 (ddd, *J* = 8.3, 7.0, 1.3 Hz, 1H), 7.52–7.47 (m, 3H), 7.40 (ddd, *J* = 8.2, 6.9, 1.2 Hz, 1H), 6.02 (br s, 1H), 3.80 (td, *J* = 7.3, 5.5 Hz, 2H), 1.79 (p, *J* = 7.6 Hz, 2H), 1.48 (p, *J* = 7.1 Hz, 2H), 1.41–1.33 (m, 4H), 0.92 (t, *J* = 7.1 Hz, 3H). ^13^C NMR (151 MHz, CDCl_3_) δ 160.6, 159.7, 138.8, 132.6, 130.3, 128.7, 128.6, 128.4, 125.5, 120.6, 113.7, 41.6, 31.8, 29.5, 27.0, 22.8, 14.2.

##### N^1^,N^1^-Dimethyl-N^3^-(2-phenylquinazolin-4-yl)propane-1,3-diamine (1.7)

Brown solid (42 mg, 0.15 mmol, 71%). ^1^H NMR (600 MHz, CDCl_3_) δ 8.56–8.53 (m, 2H), 8.43 (s, 1H), 7.88 (d, *J* = 8.3 Hz, 1H), 7.77 (d, *J* = 8.2 Hz, 1H), 7.69 (t, *J* = 7.7 Hz, 1H), 7.49–7.44 (m, 3H), 7.40 (t, *J* = 7.7 Hz, 1H), 7.40 (t, *J* = 7.5 Hz, 1H), 3.91 (q, *J* = 5.3 Hz, 2H), 2.73 (t, *J* = 5.9 Hz, 2H), 2.47 (s, 6H), 2.02 (p, *J* = 5.9 Hz, 2H). ^13^C NMR (151 MHz, CDCl_3_) δ 160.8, 160.0, 150.6, 139.4, 132.4, 130.0, 128.7, 128.5, 128.3, 125.4, 121.5, 114.4, 58.9, 45.0, 41.3, 29.8, 24.7. HRMS (ESI+) *m*/*z* calc. for C_19_H_22_N_4_ [M+H]^+^: 307.1923; found: 307.1924, [M+Na]^+^: 329.1742; found: 329.1735.

##### 4-(4-Methylpiperazin-1-yl)-2-phenylquinazoline (1.8)

Yellow solid (41 mg, 0.13 mmol, 64%). ^1^H NMR (600 MHz, CDCl_3_) δ 8.57–8.54 (m, 2H), 7.97 (dd, *J* = 8.4, 1.3 Hz, 1H), 7.90 (dd, *J* = 8.3, 1.5 Hz, 1H), 7.72 (ddd, *J* = 8.3, 6.8, 1.4 Hz, 1H), 7.51–7.41 (m, 3H), 7.41 (ddd, *J* = 8.2, 6.9, 1.3 Hz, 1H), 3.91 (t, *J* = 5.0 Hz, 4H), 2.68 (t, *J* = 4.9 Hz, 4H), 2.40 (s, 3H). ^13^C NMR (126 MHz, CDCl_3_) δ 164.9, 159.6, 153.0, 138.8, 132.5, 130.3, 129.2, 128.6, 128.4, 125.0, 124.9, 115.5, 55.1, 49.8, 46.3. HRMS (ESI+) *m*/*z* calc. for C_19_H_20_N_4_ [M+H]^+^: 305.1766; found: 305.1771.

##### 2-[4-(2-Phenylquinazolin-4-yl)piperazin-1-yl]ethanol (1.9)

Yellow solid (58 mg, 0.17 mmol, 83%). ^1^H NMR (600 MHz, CDCl_3_) δ 8.57–8.53 (m, 2H), 7.98 (d, *J* = 8.4 Hz, 1H), 7.89 (d, *J* = 8.2 Hz, 1H), 7.73 (ddd, *J* = 8.3, 6.9, 1.3 Hz, 1H), 7.51–7.46 (m, 3H), 7.42 (ddd, *J* = 8.2, 6.9, 1.2 Hz, 1H), 3.92 (t, *J* = 4.9 Hz, 4H), 3.71 (t, *J* = 5.3 Hz, 2H), 2.80 (t, *J* = 4.9 Hz, 4H), 2.68 (t, *J* = 5.3 Hz, 2H). ^13^C NMR (151 MHz, CDCl_3_) δ 165.0, 159.6, 153.0, 138.7, 132.6, 130.3, 129.3, 128.6, 128.5, 125.1, 124.9, 115.6, 59.7, 57.9, 53.0, 49.9. HRMS (ESI+) *m*/*z* calc. for C_20_H_22_N_4_O [M+H]^+^: 335.1872; found: 335.1869.

##### 2-Phenyl-N-[2-(Piperidin-1-yl)ethyl]quinazolin-4-amine (1.10)

Yellow solid (59 mg, 0.18 mmol, 84%). ^1^H NMR (500 MHz, CDCl_3_) δ 8.56 (d, *J* = 6.9 Hz, 2H), 7.91 (d, *J* = 8.3 Hz, 1H), 7.78 (d, *J* = 8.1 Hz, 1H), 7.72 (ddd, *J* = 8.3, 6.9, 1.3 Hz, 1H), 7.50–7.41 (m, 4H), 6.86 (br s, 1H), 3.87 (q, *J* = 5.5 Hz, 2H), 2.75 (t, *J* = 6.0 Hz, 2H), 2.62–2.47 (m, 4H), 1.68 (p, *J* = 5.6 Hz, 4H), 1.55–1.47 (m 2H). ^13^C NMR (151 MHz, CDCl_3_) δ 160.8, 159.8, 150.7, 139.4, 132.5, 130.1, 129.0, 128.6, 128.3, 125.5, 121.0, 114.2, 57.0, 54.5, 37.6, 26.2, 24.5. HRMS (ESI+) *m*/*z* calc. for C_21_H_24_N_4_ [M+H]^+^: 333.2079; found: 333.2071.

##### N-(1-Benzylpiperidin-4-yl)-2-phenylquinazolin-4-amine (1.11)

Yellow solid (43 mg, 0.11 mmol, 52%). ^1^H NMR (600 MHz, CDCl_3_) δ 8.56–8.52 (m, 2H), 7.92 (dd, *J* = 8.4, 1.1 Hz, 1H), 7.72 (ddd, *J* = 8.3, 6.9, 1.3 Hz, 1H), 7.68 (dd, *J* = 8.3, 1.3 Hz, 1H), 7.53–7.45 (m, 3H), 7.42 (ddd, *J* = 8.2, 6.9, 1.2 Hz, 1H), 7.40–7.32 (m, 4H), 7.31–7.27 (m, 1H), 5.56 (d, *J* = 7.3 Hz, 1H), 4.49–4.43 (m, 1H), 3.00–2.92 (m, 2H), 2.33 (td, *J* = 11.5, 2.5 Hz, 2H), 2.27–2.23 (m, 2H), 1.75–1.68 (m, 2H). ^13^C NMR (151 MHz, CDCl_3_) δ 160.6, 159.0, 150.8, 139.2, 138.4, 132.6, 130.2, 129.3, 129.1, 128.5, 128.4, 128.4, 127.3, 125.5, 120.5, 113.8, 63.3, 52.6, 48.3, 32.2. HRMS (ESI+) *m*/*z* calc. for C_26_H_26_N_4_ [M+H]^+^: 395.2239; found: 395.2229.

##### 2-Phenyl-N-(2,2,6,6-tetramethylpiperidin-4-yl)quinazolin-4-amine (1.12)

White solid (26 mg, 0.07 mmol, 34%). ^1^H NMR (600 MHz, CDCl_3_) δ 8.60–8.56 (m, 2H), 7.92 (d, *J* = 8.3 Hz, 1H), 7.73 (t, *J* = 7.7 Hz, 1H), 7.67 (d, *J* = 8.2 Hz, 1H), 7.49–7.46 (m, 3H), 7.42 (t, *J* = 7.6 Hz, 1H), 5.43 (d, *J* = 7.1 Hz, 1H), 4.96–4.91 (m, 1H), 2.26 (dd, *J* = 12.2, 3.6 Hz, 2H), 1.56 (s, 1H), 1.45 (s, 6H), 1.21 (s, 6H), 1.15 (t, *J* = 12.0 Hz, 2H). ^13^C NMR (151 MHz, CDCl_3_) δ 160.5, 158.9, 150.8, 139.1, 132.6, 130.2, 129.2, 128.4, 128.4, 125.4, 120.4, 113.8, 51.6, 45.2, 44.4, 35.1, 28.8. HRMS (ESI+) *m*/*z* calc. for C_23_H_28_N_4_ [M+H]^+^: 361.2392; found: 361.2383.

##### 4-{[(2-Phenylquinazolin-4-yl)amino]methyl}pyridine 1-oxide (1.13)

White solid (50 mg, 0.15 mmol, 73%). ^1^H NMR (600 MHz, DMSO-*d*_6_) δ 8.97 (t, *J* = 5.9 Hz, 1H), 8.72 (d, *J* = 2.3 Hz, 1H), 8.47–8.41 (m, 3H), 8.32–8.26 (m, 1H), 7.85 (dt, *J* = 7.9, 2.0 Hz, 1H), 7.81–7.77 (m, 2H), 7.52 (ddd, *J* = 8.2, 5.2, 2.9 Hz, 1H), 7.47 (dd, *J* = 5.2, 2.0 Hz, 3H), 7.35 (ddd, *J* = 7.9, 4.8, 0.9 Hz, 1H), 4.92 (d, *J* = 5.7 Hz, 2H). ^13^C NMR (151 MHz, DMSO-*d*_6_) δ 159.5, 159.1, 149.9, 149.0, 148.1, 138.5, 135.3, 135.2, 132.9, 130.1, 128.2, 127.9, 127.8, 125.5, 123.5, 122.7, 113.8, 79.2, 41.6, 39.9, 39.8, 39.7, 39.5, 39.4, 39.2, 39.1. HRMS (ESI+) *m*/*z* calc. for C_20_H_16_N_4_O [M+H]^+^: 329.1402; found: 329.1407.

##### 4-(4-Methylpiperazin-1-yl)-2-(4-nitrophenyl)quinazoline (1.14)

Yellow solid (39 mg, 0.11 mmol, 53%). ^1^H NMR (600 MHz, CDCl_3_) δ 8.74–8.69 (m, 2H), 8.34–8.30 (m, 2H), 7.98 (dd, *J* = 8.4, 1.1 Hz, 1H), 7.92 (dd, *J* = 8.4, 1.3 Hz, 1H), 7.77 (ddd, *J* = 8.4, 6.9, 1.4 Hz, 1H), 7.48 (ddd, *J* = 8.3, 6.9, 1.2 Hz, 1H), 3.94 (t, *J* = 5.0 Hz, 4H), 2.68 (t, *J* = 5.0 Hz, 4H), 2.41 (s, 3H). ^13^C NMR (151 MHz, CDCl_3_) δ 164.8, 157.3, 152.8, 149.0, 144.8, 132.9, 129.4, 129.3, 125.9, 125.1, 123.6, 115.6, 55.0, 49.8, 46.3. HRMS (ESI+) *m*/*z* calc. for C_19_H_19_N_5_O_2_ [M+H]^+^: 350.1617; found: 350.1626.

##### 2-(4-Nitrophenyl)-N-[2-(piperidin-1-yl)ethyl]quinazolin-4-amine (1.15)

Yellow solid (40 mg, 0.10 mmol, 50%). ^1^H NMR (600 MHz, CDCl_3_) δ 8.79–8.72 (m, 2H), 8.35–8.30 (m, 2H), 7.97–7.92 (m, 1H), 7.82–7.77 (m, 2H), 7.53 (td, *J* = 7.4, 1.2 Hz, 1H), 6.97 (br s, 1H), 3.86 (q, *J* = 5.7 Hz, 2H), 2.77 (t, *J* = 6.0 Hz, 2H), 2.56 (br s, 4H), 1.69 (p, *J* = 5.6 Hz, 4H), 1.57–1.53 (m, 2H). ^13^C NMR (151 MHz, CDCl_3_) δ 159.8, 158.5, 150.2, 148.9, 145.3, 132.9, 129.3, 129.1, 126.4, 123.5, 121.1, 114.3, 56.7, 54.4, 37.6, 26.2, 24.4. HRMS (ESI+) *m*/*z* calc. for C_21_H_23_N_5_O_2_ [M+H]^+^: 378.1930; found: 378.1934.

##### 2-{4-[2-(4-Nitrophenyl)quinazolin-4-yl]piperazin-1-yl}ethanol (1.16)

Yellow solid (31 mg, 0.08 mmol, 39%). ^1^H NMR (600 MHz, CDCl_3_) δ 8.75–8.69 (m, 2H), 8.35–8.29 (m, 2H), 8.00 (dd, *J* = 8.4, 1.2 Hz, 1H), 7.92 (dd, *J* = 8.3, 1.0 Hz, 1H), 7.78 (ddd, *J* = 8.4, 6.9, 1.4 Hz, 1H), 7.49 (ddd, *J* = 8.2, 6.9, 1.2 Hz, 1H), 3.95 (t, *J* = 4.6 Hz, 4H), 3.75–3.70 (m, 2H), 2.81 (t, *J* = 5.0 Hz, 4H), 2.69 (t, *J* = 5.3 Hz, 2H). ^13^C NMR (151 MHz, CDCl_3_) δ 164.9, 157.4, 152.8, 149.1, 144.7, 133.1, 129.5, 129.3, 126.1, 125.0, 123.7, 115.6, 59.8, 57.8, 53.0, 49.6. HRMS (ESI+) *m*/*z* calc. for C_20_H_21_N_5_O_3_ [M+H]^+^: 380.1723; found: 380.1719.

##### 2-{4-[2-(2,4-Dichlorophenyl)quinazolin-4-yl]piperazin-1-yl}ethanol (1.17)

Yellow solid (34 mg, 0.08 mmol, 40%). ^1^H NMR (600 MHz, CDCl_3_) δ 7.91 (d, *J* = 8.2 Hz, 1H), 7.80–7.74 (m, 3H), 7.54–7.48 (m, 2H), 7.34 (dd, *J* = 8.3, 2.1 Hz, 1H), 6.62 (t, *J* = 4.5 Hz, 1H), 3.79–3.76 (m, 6H), 2.73 (t, *J* = 6.0 Hz, 2H), 2.56 (t, *J* = 4.6 Hz, 4H). ^13^C NMR (151 MHz, CDCl_3_) δ 161.1, 159.5, 150.0, 137.9, 135.0, 133.9, 132.9, 132.6, 130.4, 129.0, 127.1, 126.4, 120.7, 113.6, 67.2, 56.8, 53.5, 37.3. HRMS (ESI+) *m*/*z* calc. for C_20_H_20_Cl_2_N_4_O [M+H]^+^: 403.1092; found: 403.1097.

##### 2-(4-{2-[4-(N,N-Dimethylamino)phenyl]quinazolin-4-yl}piperazin-1-yl)ethanol (1.18)

Yellow solid (32 mg, 0.09 mmol, 41%). ^1^H NMR (500 MHz, DMSO-*d*_6_) δ 8.34–8.30 (m, 1H), 7.93 (dd, *J* = 8.3, 1.3 Hz, 1H), 7.80–7.72 (m, 2H), 7.41 (ddd, *J* = 8.3, 6.6, 1.6 Hz, 1H), 6.83–6.77 (m, 2H), 4.47 (br s, 1H), 3.76 (t, *J* = 4.8 Hz, 4H), 3.59–3.53 (m, 2H), 3.00 (s, 6H), 2.66 (t, *J* = 4.8 Hz, 4H), 2.48 (t, *J* = 6.2 Hz, 1H, overlapping with DMSO). ^13^C NMR (151 MHz, DMSO-*d*_6_) δ 163.9, 158.7, 152.4, 151.8, 132.6, 129.4, 129.2, 127.9, 125.2, 124.2, 114.3, 111.3, 69.8, 60.3, 58.5, 53.0, 49.3, 39.5.

##### 2-(4-Methoxyphenyl)-4-(4-Methylpiperazin-1-yl)quinazoline (1.19)

Yellow solid (24 mg, 0.07 mmol, 35%). ^1^H NMR (600 MHz, DMSO-*d*_6_) δ 8.45–8.41 (m, 2H), 7.97 (dd, *J* = 8.3, 1.3 Hz, 1H), 7.84 (dd, *J* = 8.4, 1.3 Hz, 1H), 7.79 (ddd, *J* = 8.3, 6.8, 1.3 Hz, 1H), 7.47 (ddd, *J* = 8.3, 6.8, 1.4 Hz, 1H), 7.08–7.04 (m, 2H), 3.83 (s, 3H), 3.80 (t, *J* = 5.0 Hz, 4H), 2.56 (t, *J* = 4.9 Hz, 4H), 2.26 (s, 3H). ^13^C NMR (151 MHz, DMSO-*d*_6_) δ 164.0, 161.2, 158.0, 152.2, 132.8, 130.6, 129.6, 128.2, 125.3, 124.9, 114.5, 113.7, 55.3, 54.5, 49.2, 45.8.

##### 2-{4-[2-(p-Tolyl)quinazolin-4-yl]piperazin-1-yl}ethanol (1.20)

White solid (41 mg, 0.12 mmol, 56%). ^1^H NMR (600 MHz, DMSO-*d*_6_) δ 8.38 (d, *J* = 8.0 Hz, 2H), 7.99 (d, *J* = 8.3 Hz, 1H), 7.86 (dd, *J* = 8.5, 1.2 Hz, 1H), 7.80 (dd, *J* = 8.4, 6.8 Hz, 1H), 7.49 (t, *J* = 7.6 Hz, 1H), 7.32 (d, *J* = 8.0 Hz, 2H), 4.48 (br s, 1H), 3.81 (t, *J* = 4.7 Hz, 4H), 3.56 (t, *J* = 6.2 Hz, 2H), 2.67 (t, *J* = 4.8 Hz, 4H), 2.48 (t, *J* = 6.2 Hz, 2H, overlapping with DMSO), 2.38 (s, 3H). ^13^C NMR (151 MHz, DMSO-*d*_6_) δ 164.0, 158.1, 152.1, 140.0, 135.4, 132.8, 129.0, 128.2, 127.9, 125.3, 125.0, 114.6, 60.2, 58.5, 53.0, 49.2, 21.0.

##### 7-Chloro-N-(2-Morpholinoethyl)quinazolin-4-amine (1.21)

White solid (36 mg, 0.12 mmol, 59%). ^1^H NMR (600 MHz, CDCl_3_) δ 8.66 (s, 1H), 7.85 (d, *J* = 1.9 Hz, 1H), 7.67 (d, *J* = 8.7 Hz, 1H), 7.45 (dd, *J* = 8.7, 2.0 Hz, 1H), 6.64 (s, 1H), 3.77 (dt, *J* = 42.5, 5.0 Hz, 7H), 2.77 (t, *J* = 5.9 Hz, 2H), 2.60 (s, 4H). ^13^C NMR (151 MHz, CDCl_3_) δ 159.1, 156.4, 150.3, 127.7, 127.4, 126.8, 122.2, 113.4, 67.0, 56.4, 53.2, 37.0. HRMS (ESI+) *m*/*z* calc. for C_14_H_17_ClN_4_O [M+H]^+^: 293.1091; found: 293.1122.

##### 2-[4-(7-Chloroquinazolin-4-yl)piperazin-1-yl]ethan-1-ol (1.22)

Yellowish solid (15 mg, 0.05 mmol, 24%). ^1^H NMR (500 MHz, DMSO-*d*_6_) δ 8.60 (s, 1H), 8.01 (d, *J* = 8.9 Hz, 1H), 7.83 (d, *J* = 2.2 Hz, 1H), 7.53 (dd, *J* = 8.9, 2.2 Hz, 1H), 4.46 (t, *J* = 5.3 Hz, 1H), 3.77–3.72 (m, 4H), 3.54 (q, *J* = 5.8 Hz, 2H), 2.61–2.58 (m, 4H), 2.46 (t, *J* = 6.2 Hz, 2H). ^13^C NMR (151 MHz, DMSO-*d*_6_) δ 163.2, 154.7, 152.3, 137.2, 127.6, 126.6, 125.8, 114.2, 60.1, 58.5, 53.0, 49.1.

##### 7-Chloro-N-[2-(Piperidin-1-yl)ethyl]quinazolin-4-amine (1.23)

Yellow solid (17 mg, 0.06 mmol, 28%). ^1^H NMR (600 MHz, CDCl_3_) δ 8.61 (s, 1H), 7.79 (d, *J* = 2.1 Hz, 1H), 7.67 (d, *J* = 8.7 Hz, 1H), 7.40 (dd, *J* = 8.8, 2.1 Hz, 1H), 6.97 (s, 1H), 3.67–3.62 (m, 2H), 2.68 (t, *J* = 5.9 Hz, 2H), 2.54–2.41 (m, 4H), 1.63 (p, *J* = 5.6 Hz, 4H), 1.53–1.47 (m, 2H). ^13^C NMR (151 MHz, CDCl_3_) δ 159.3, 156.6, 150.4, 138.6, 127.7, 126.8, 122.6, 113.7, 56.5, 54.2, 37.4, 26.2, 24.4. HRMS (ESI+) *m*/*z* calc. for C_15_H_19_ClN_4_ [M+H]^+^: 291.1376; found: 291.1366.

##### 7-Chloro-4-(4-Methylpiperazin-1-yl)quinazoline (1.24)

Yellowish solid (14 mg, 0.05 mmol, 25%). ^1^H NMR (600 MHz, CDCl_3_) δ 8.69 (s, 1H), 7.86 (d, *J* = 1.9 Hz, 2H), 7.79 (d, *J* = 8.9 Hz, 1H), 7.38 (dd, *J* = 8.9, 2.1 Hz, 1H), 3.81 (t, *J* = 4.9 Hz, 4H), 2.60 (t, *J* = 4.9 Hz, 4H), 2.37 (s, 3H). ^13^C NMR (151 MHz, CDCl_3_) δ 164.4, 155.2, 152.9, 138.6, 127.8, 126.5, 126.2, 115.0, 55.0, 49.7, 46.2. HRMS (ESI+) *m*/*z* calc. for C_13_H_15_ClN_4_ [M+H]^+^: 263.1063; found: 263.1068.

#### Molecular Modeling

For the molecular docking study, the RdRp protein structure was obtained from the RCSB Protein Data Bank (PDB: 1S48) (Choi et al., 2004). UCSF Chimera was used to mutate the MSE-modified amino acid to MET. Ligands and protein were then saved in a PDBQT file format for input into AutoDock Vina to carry out docking calculations (Trott and Olson, 2010). ADT was used to remove crystal waters, add polar hydrogens, and assign Gasteiger charges to the protein structure (Maier et al., 2015). For RdRp, the search space was a box with 30 × 30 × 30 Å XYZ dimensions, respectively, and was centered on −17, 177, and 19 XYZ coordinates, respectively.

MD simulations were performed using the Amber16 and AmberTools16 package (Case et al., 2016) with the Amber14SB force field (Maier et al., 2015). The docked poses of the compounds were employed as the initial configuration for the system. The TIP3P water model (Jorgensen et al., 1983) was used in a truncated octahedral box, extending 10 Å from the protein. A physiological salt concentration of 0.15 M was set by employing Na^+^ and Cl^−^ ions.

The simulations were performed using the PMEMD CUDA module of the Amber simulation suite and consisted of the following steps: an initial 1-ps run with a 0.01-fs timestep to eliminate bad contacts followed by an energy minimization; heating from 0 to 10 K over 10 ps with a 0.1-fs timestep with strong restraints (50 kcal mol^−1^ Å^−2^) on the protein residues and ligands; and then from 10 to 300 K over 90 ps with a 0.5-fs timestep and weaker restraints (10 kcal mol^−1^ Å^−2^). The system was then equilibrated for 400 ps at constant temperature and pressure with weak restraints on the ligands and on the CA atoms of the protein (1 kcal mol^−1^ Å^−2^). The Langevin thermostat was used with a collision frequency of 2.0 ps^−1^ (Davidchack et al., 2009); the SHAKE algorithm was used to constrain bonds, allowing a 2-fs timestep; and an 8.0-Å cutoff was used for non-bonded interactions. Finally, a 100-ns production simulation was performed under the NPT conditions described above.

### Biological Assay

#### Test Compounds

All compounds were solubilized in DMSO at a concentration of 5 and 1 mg/ml and subsequently serially diluted in a cell culture medium. The highest concentration evaluated of each compound contained DMSO at a final concentration <1% (non-cytotoxic concentration). The thiosemicarbazone of 5,6-dimethoxy-1-indanone (TSC) was employed as a reference inhibitor of BVDV RdRp (Finkielsztein et al., 2008; Castro et al., 2011) (EC_50_ = 1.8 ± 0.6 μM). Finally, the stability of compounds in DMSO and water:DMSO (98:2) was evaluated as described in the Supplementary Material.

#### Cells and Virus

Madin-Darby bovine kidney cells (MDBK NBL-1; ATCC CCL-22) were grown in Eagle's Minimum Essential Medium (E-MEM), supplemented with 10% biotechnological fetal bovine serum (Internegocios, Argentina) (GM) at 37°C and 5% CO_2_. The BVDV type 1 NADL strain, cytopathic biotype (BVDV-1, ATCC VR 534), was provided by Dr. Laura Weber (INTA Castelar, Argentina).

#### Cytotoxicity Assay

MDBK cells were seeded in 96-well plates at a density of 1 × 10^4^ cells per well in GM. After 24 h, the culture medium was removed, monolayers were washed twice with phosphate-buffered saline (PBS), and serial dilutions of the test compounds were made in E-MEM supplemented with donor horse serum (DHS, Gibco, IM) and added. Cultures were incubated for 3 days at 37°C, and then, cell viability was determined by the MTS/PMS method according to the manufacturer's instructions (Promega). After incubation for 3 h at 37°C and 5% CO_2_, the absorbance was determined at 490 nm. The 50% cytotoxic concentration (CC_50_) was defined as the concentration of compound that inhibited the proliferation of exponentially growing cells by 50% and was calculated by interpolation in dose–response curves.

#### Anti-BVDV Assay

MDBK cells were seeded in 96-well microplates at a density of 1 × 10^4^ cells per well in GM. After incubation for 24 h at 37°C, the culture medium was removed, and the monolayers were washed twice with PBS and infected with BVDV with an inoculum that resulted in a >80% cytopathic effect (CPE) after 3 days of incubation at 37°C (MOI = ~0.01 PFU/cell). Then, serial dilutions of the test compounds in IM were added. Mock-infected cells and mock-treated infected cells were included as controls in each plate. After incubation for 3 days at 37°C and 5% CO_2_, the culture medium was removed, cell monolayers were washed twice with PBS, and IM supplemented with MTS/PMS was added to each well. The absorbance of each well was read at 490 nm. The EC_50_ was defined as the concentration of compound that prevented 50% of the virus-induced CPE and was calculated by interpolation in dose–response curves.

#### Cross-Resistance Assay

In order to determine the mechanism of antiviral action of this class of compounds, anti-BVDV assays were performed as stated above with different BVDV NADLs that are resistant to TSC: BVDV R1 (NS5B A392E) and BVDV R2 (NS5B N264D). Compound **1.9** was used at its maximum non-cytotoxic concentration (12.5 μM). As positive control, TSC was used at 34.0 μM (20 × EC_50_). Mock-infected cells and mock-treated infected cells were included as controls in each assay plate. After 3 days, the culture medium was removed, cell monolayers were washed twice with PBS, and IM supplemented with MTS/PMS solution was added to each well. The percentage of CPE inhibition was calculated for each experimental condition.

### Solubility

#### Shake-Flask Method for Drug Solubility Testing

The shake-flask method was used as a reference assay (Alsenz and Kansy, 2007; Baka et al., 2008; Bollini et al., 2013). Compound **1.9** (1.0 mg) was measured in simulated gastric fluid (SGF, pH 1.2), simulated intestinal fluid (SIF, pH 6.8), and PBS solution (pH 7.4) by UV-Vis-NIR spectrophotometry. Verapamil was used as internal control. More detailed information is available in the Supplementary Material.

### Stability

#### SGF, SIF, PBS, Mouse, and Bovine Plasma Stability

Stability assays were performed according to the procedure described in a previous work (Leal et al., 2019). Samples were taken at different incubation times and analyzed by high-performance liquid chromatography–mass spectrometry (HPLC–MS) using a Waters Alliance e2695 system (Waters Corporation, Milford, MA, USA) fitted with a Phenomenex Kinetex® XB-C18 column (150 × 4.6 mm, 5-μm particle size, Phenomenex Inc., Torrance, CA, USA) coupled to a Waters SQD2 single quadrupole mass spectrometer with an ESI source. A gradient elution was applied using 40% water and 0.1% acetic acid (mobile phase A) and 40% methanol (mobile phase B), with the following program: 0–3 min 40% B; 3–7 min 40–90% B; and 7–20 min 90% B. The flow rate was kept constant at 0.35 ml min^−1^. After each sample injection, the gradient was returned to its initial conditions in 16 min. The injection volume was 5 μl. The column temperature was 35°C. The mass spectrometer was operated in positive ion mode with a probe capillary voltage of 2.5 kV. The sampling cone voltage was set to 35.0 V. The source and desolvation gas temperatures were set at 150 and 350°C, respectively. The nitrogen gas desolvation flow rate was 600 L h^−1^, and the cone gas flow rate was 10 L h^−1^. The mass spectrometer was calibrated across the range of *m*/*z* 20–2,023 with a sodium and cesium iodide solution. Data were acquired in scan mode with a scan duration of 0.2 s and in SIR mode with unit resolution. Data acquisition and processing were carried out using MassLynx software, version 4.1. Enalapril and salicylaldehyde isonicotinoyl hydrazine (SIH) (Kovaríková et al., 2008) were used as positive controls for murine and bovine plasma, respectively. SIH was synthesized according to the procedures described by Gaonkar et al. (2006) and Trzesowska-Kruszynska (2013).

## Results and Discussion

### Chemistry

#### Synthesis of Quinazoline Derivatives

Compound **1** emerged as one of the most promising scaffolds from our structure-based virtual screening approach toward the development of RdRp inhibitors (Castro et al., 2019). To improve the antiviral activity, we prepared a range of quinazoline analogs introducing modification at positions 4, 2, and 7 of the quinazoline scaffold (**1.1**–**1.24**).

**Scheme 1:**
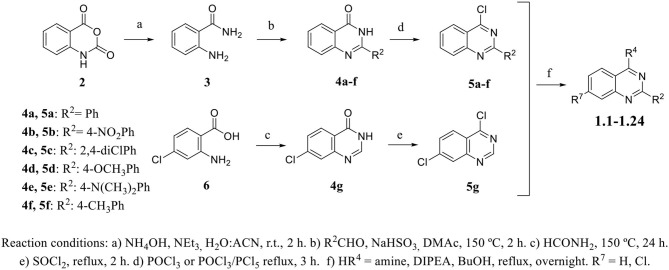
General procedure for the synthesis of quinazoline derivatives.

*Ortho*-aminobenzamide (**3**) was obtained through the reaction between isatoic anhydride (**2**) and ammonia in the presence of triethylamine in an acetonitrile/methanol (1:1) mixture (41–100%) (Hour et al., 2000; Storz et al., 2011; Bollini et al., 2019). Quinazolinone scaffolds (**4a**–**4f**) were obtained through the reaction between **3**, benzaldehyde, and sodium hydrogen sulfite in *,N*-dimethylacetamide (DMAc) (43–63%). The chlorination of 4-quinazolinones with POCl_3_ or POCl_3_/PCl_5_ afforded 4-chloro-2-phenylquinazoline derivatives (**5a**–**5f**, 48–99%). Compound **4g** was synthesized by reaction between 2-amino-4-chlorobenzoic acid (**6**) and formamide under reflux, followed by recrystallization from acetonitrile to give a product with an 80% yield. The subsequent step was performed with SOCl_2_ under reflux to yield compound **5g** (83%). The derivatives **1.1**–**1.24** were obtained through an aromatic nucleophilic substitution reaction with amines or anilines in the presence of DIPEA in *n*-butanol under reflux conditions. The compounds were purified by preparative TLC in DCM/MeOH mixtures (95:5 or 90:10) to give products with a 24–84% yield (Scheme 1). Procedure details are presented in the Supplementary Material.

#### Antiviral Activity Against wtBVDV

The synthesized compounds (**1.1**–**1.24**) were evaluated for their antiviral potency against BVDV in cultured MDBK cells employing TSC as antiviral activity positive control. The values of EC_50_, CC_50_, and selectivity index (SI, CC_50_/EC_50_ ratio) of the target compounds are summarized in Figure 1 and Table 1.

**Table 1:** Cytotoxicity and anti-BVDV activity of compound **1** derivatives.

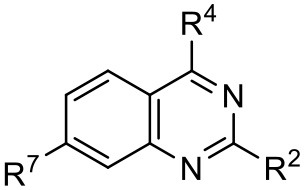							
**Compd**.	**R**^**2**^	**R**^**4**^	**R**^**7**^	**CC**_**50**_ **(μM)^a^**	**MNCC (μM)^b^**	**EC**_**50**_ **(μM)^c^**	**SI^d^**
**1**	Ph	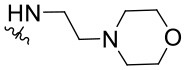	H	55.9 ± 8.2	10.9 ± 1.4	9.7 ± 0.5	5.8
**1.1**	Ph	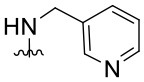	H	UD^e^	12.0 ± 0.1	Inactive^f^	-
**1.2**	Ph	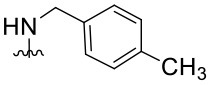	H	UD	6.0 ± 0.2	Inactive	-
**1.3**	Ph	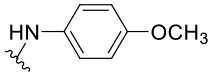	H	UD	6.0 ± 0.2	Inactive	-
**1.4**	Ph	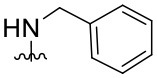	H	UD	6.0 ± 0.3	Inactive	-
**1.5**	Ph	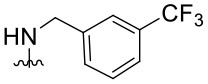	H	UD	6.0 ± 0.1	Inactive	-
**1.6**	Ph	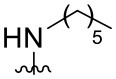	H	UD	12.0 ± 0.2	Inactive	-
**1.7**	Ph	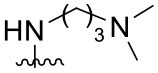	H	57.0 ± 0.01	18.6 ± 2.6	4.6 ± 1.0	12.4
**1.8**	Ph	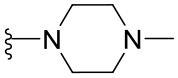	H	68.0 ± 8.4	19.8 ± 4.0	1.4 ± 0.1	48.6
**1.9**	Ph	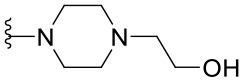	H	32.0 ± 1.9	12.5 ± 2.4	1.8 ± 0.4	17.8
**1.10**	Ph	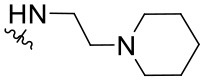	H	19.6 ± 6.3	6.4 ± 1.1	1.4 ± 0.8	14.0
**1.11**	Ph	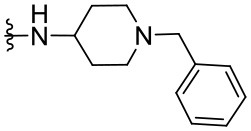	H	22.2 ± 1.2	7.5 ± 0.1	Inactive	-
**1.12**	Ph	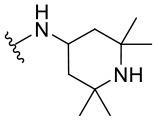	H	7.4 ± 0.03	3.3 ± 0.7	1.4 ± 0.3	5.3
**1.13**	Ph	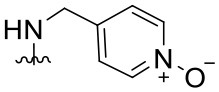	H	>100.0	-	Inactive	-
**1.14**	4-NO_2_Ph	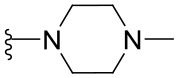	H	13.0 ± 3.4	8.7 ± 1.6	Inactive	-
**1.15**	4-NO_2_Ph	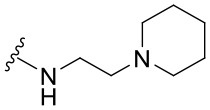	H	30.2 ± 0.5	14.6 ± 3.1	5.8 ± 0.6	5.2
**1.16**	4-NO_2_Ph	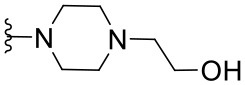	H	12.0 ± 2.1	5.9 ± 1.4	Inactive	-
**1.17**	2,4-diClPh	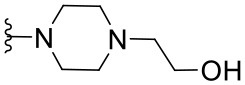	H	31.5 ± 5.7	17.4 ± 3.0	Inactive	-
**1.18**	4-(NMe_2_)Ph	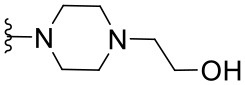	H	61.0 ± 2.5	26.1 ± 1.2	3.5 ± 0.6	17.4
**1.19**	4-OMePh	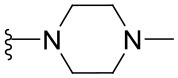	H	37.1 ± 0.2	18.3 ± 0.3	12.8 ± 0.3	2.9
**1.20**	4-MePh	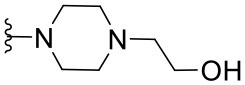	H	55.4 ± 4.0	30.5 ± 2.1	8.5 ± 3.4	6.5
**1.21**	H	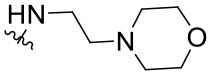	Cl	59.3 ± 10.7	17.3 ± 5.2	2.3 ± 0.5	25.8
**1.22**	H	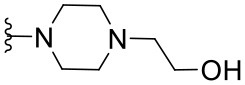	Cl	>100.0	-	25.8 ± 1.2	-
**1.23**	H	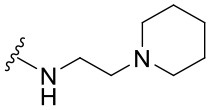	Cl	>100.0	-	4.1 ± 2.2	>24.4
**1.24**	H	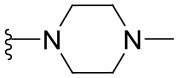	Cl	>100.0	-	9.3 ± 2.1	>10.8

a *CC_50_: Compound concentration that reduces cell viability by 50%*.

b *MNCC: Maximum non-cytotoxic concentration*.

c *EC_50_: Compound concentration that reduces viral CPE by 50%. Data are expressed as mean values ± SD from at least three independent experiments*.

d *CC_50_/EC_50_: In vitro selectivity index*.

e *UD: Undetermined*.

f *Inactive: <50% of inhibition of CPE at MNCC. 5,6-TSC EC_50_ 1.8 ± 0.6 μM*.

Results demonstrated that the antiviral activity of compounds **1.7**–**1.10**, **1.12**, **1.18**, **1.21**, and **1.23** was higher than that of the lead compound **1**, with EC_50_ values ranging from 1.3 to 4.6 μM (Figure 1). Among them, **1.8** (EC_50_ = 1.4 ± 0.1 μM) and **1.9** (EC_50_ = 1.8 ± 0.4 μM) proved to be the most potent inhibitors with good SIs. A high *in vitro* SI suggests a specific inhibition of a viral target, which would result in an *in vivo* higher therapeutic index.

**Figure 2:**
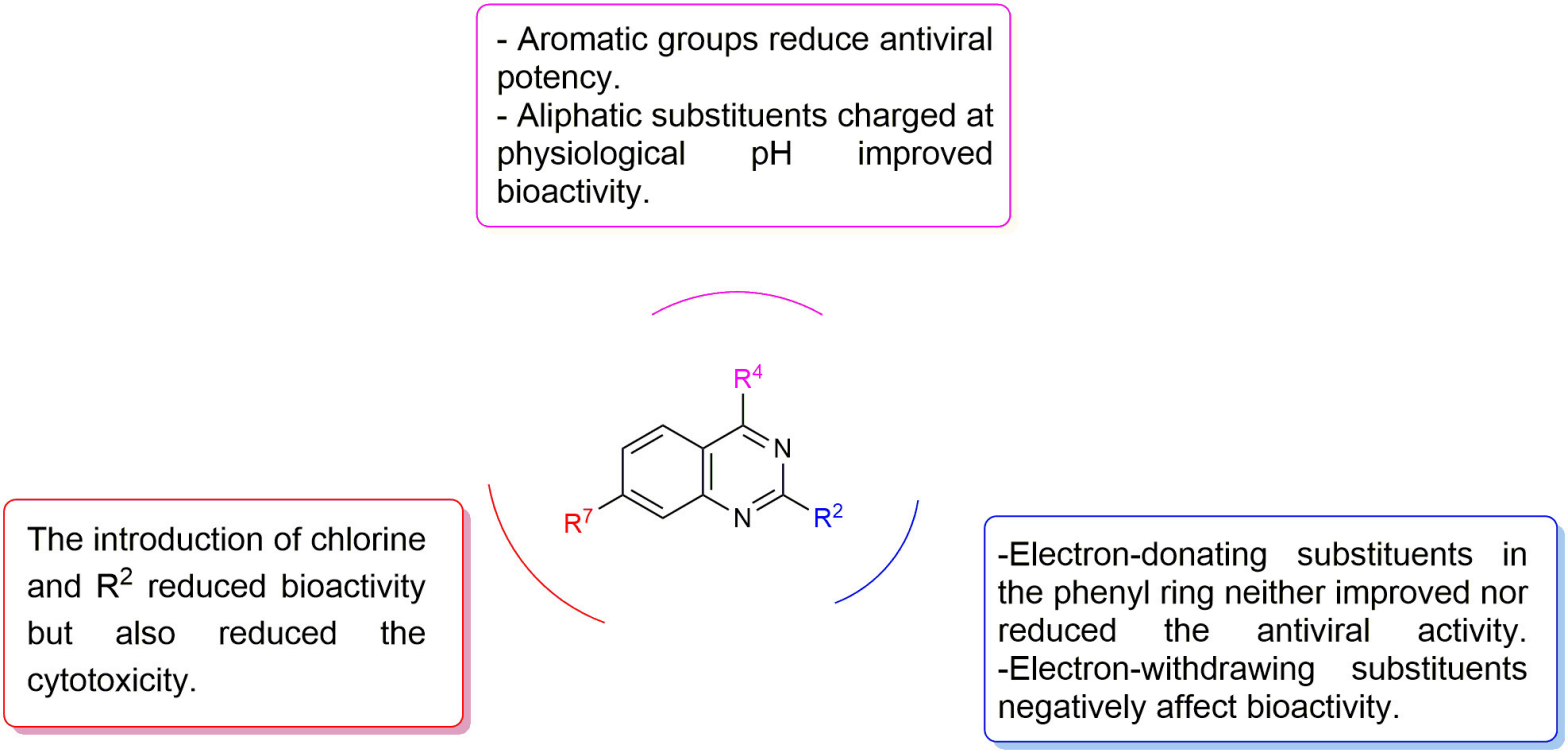
Summary of SAR analysis of quinazoline analogs.

Preliminary SAR studies have revealed that different substituents located at position 4 on the quinazoline core have significant effects on the antiviral potency (Figure 2). For instance, aromatic groups like benzyl, methyl pyridine, and methyl pyridine *N*-oxide sharply reduced the antiviral potency (**1.1**–**1.5** and **1.13**). However, the presence of charges at physiological pH on the R^4^ substituent conferred the compound higher bioactivity (**1.7**–**1.10** and **1.12**). Interestingly, the replacement of the *N*-hexyl substituent (**1.6**) for the polar *N,N*-dimethyl propane diamine (**1.7**) contributed greatly to the activity.

The impact of the substitution in the aromatic ring at R^2^ of compounds **1.8** and **1.9** on the antiviral activity was also assessed. The introduction of electron-donating substituents in the phenyl ring, such as *N,N*-dimethyl (**1.18**, EC_50_ = 3.5 ± 0.6 μM), methoxy (**1.19**, EC_50_ = 12.8 ± 0.2 μM), or methyl group (**1.20**, EC_50_ = 8.5 ± 3.4 μM) generated analogs with either similar or reduced antiviral activity, as compared to the lead compounds **1.8** and **1.9** (EC_50_ = 1.4 ± 0.1 and 1.8 ± 0.4 μM, respectively). However, the incorporation of a nitro group (**1.14**–**1.16**) as a strong electron-withdrawing substituent or two halogen atoms (**1.17**) in the phenyl group gave rise to either less active or inactive compounds.

Furthermore, four derivatives (**1.21**–**1.24**) lacking the phenyl substitution at position 2 of the quinazoline core were synthesized. Except compound **1.10**, all the derivatives showed lower potency than their corresponding analogs (**1** and **1.8**–**1.10**). In addition, this series of compounds showed considerably low cytotoxicity.

#### Molecular Modeling

To characterize the interactions between the compounds and the allosteric site of the RdRp, we performed docking and MD simulation analyses. The docked poses were used as initial system configurations, and all protein–ligand complexes remained stable along each 100-ns MD simulations (Supplementary Figure 1).

**Figure 3:**
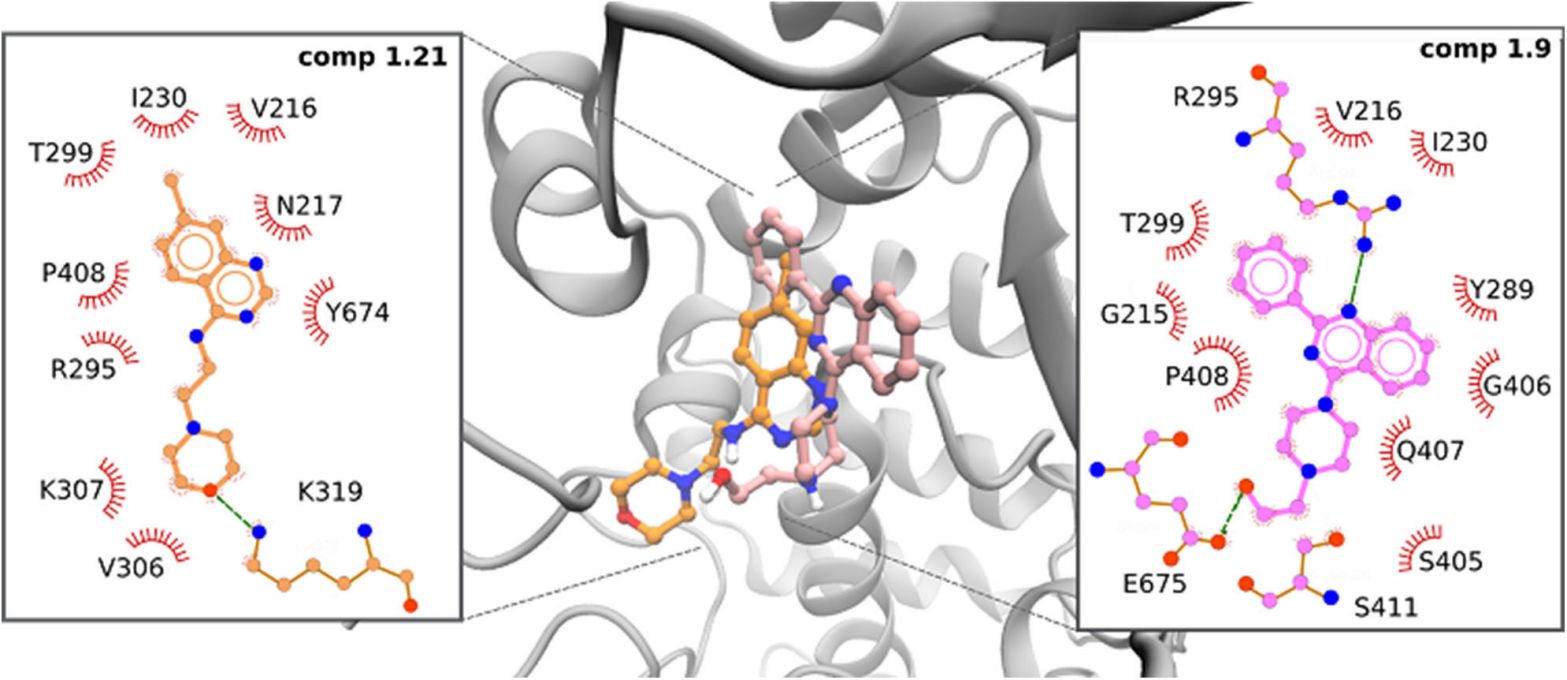
Predicted interactions of active compounds **1.9** (pink) and **1.21** (orange) within the allosteric site of BVDV RdRp protein obtained by MD simulations.

In particular, the phenyl quinazoline **1.9** presented two favorable hydrogen bond interactions between the HN atoms of the ligand and the O atom in R295 and E675. In both cases, interatomic distances and angles were very favorable, with average values of 2 Å and 150°, respectively (Figure 3 and Supplementary Figure 2). This ligand also presented a parallel-displaced π-π interaction with Y289.

The 7-chloroquinazoline series (**1.21**–**1.24**) presented different binding modes with respect to phenyl quinazoline analogs, as these compounds move slightly inside the binding pocket during the interaction. The most active compound of this series (**1.21**) established a hydrogen bond of moderate strength between the oxygen of the morpholine ring and the NH of K319. The system was further stabilized by close contacts with Y674, R295, L387, V216, P408, and V306 (Figure 3).

In a previous work (Castro et al., 2019), we reported a quinoline compound [2-(4-ethylphenyl)-*N*-isopentylquinolin-4-amine] with the potential to inhibit essential processes during viral RNA synthesis. A computational model showed the importance of the interaction of this compound with Y674, R295, and N217 located in the fingers and thumb domains of BVDV RdRp. Interestingly, these new compounds (**1.9** and **1.21**) accommodate their quinazoline moiety into the deep hydrophobic pocket, similarly to quinoline derivatives.

The BVDV RdRp structure has the shape of a right hand, comprising the fingers, palm, and thumb domains. In the RdRp structure, R295, Y289, and V306 are located in the fingers domain and are part of the motif II of the polymerase, which is conserved between different RNA polymerases of positive-strand RNA viruses. This motif was shown to establish contact with the phosphate backbone and bases; and it was reported that the substitution at the residue R295 completely abolished RNA synthesis. On the other hand, Y674 and E675 are located within the C-terminal loop in the thumb domain. The β-thumb region interacts with the fingers and palm domains through this long C-terminal loop (residues 670–679). Together with a loop between α-20 and α-21 in the thumb domain, the β-thumb reduces the volume of the template channel, and the C-terminal loop, in combination with the fingertip region, facilitates the translocation of the template and product RNA. Interestingly, similar to compounds **1.9** and **1.21**, docking analysis indicates that active compounds interact with R295 in the fingertip domain of the polymerase, providing a possible explanation to their mode of action (Supplementary Figure 3).

Molecular modeling studies performed with most previously reported BVDV NNI inhibitors, such as TSC (Castro et al., 2011), BPIP (Paeshuyse et al., 2007), VP32947 (Baginski et al., 2000), LZ37 (Paeshuyse et al., 2009), benzimidazole (Asthana et al., 2013), and CSFCII (Musiu et al., 2020), have shown that these compounds bind to a hydrophobic cavity limited by amino acids F224, I390, A392, and L225. This allosteric site was defined as a “hot spot” for the inhibition of virus replication.

In the present work, a new series of potential BVDV NNIs were identified. According to our observations, these new quinazoline derivatives would interact with different hydrophobic pockets. Our results are in line with previously reported studies demonstrating that the quinoxaline derivatives that are active against BVDV would act as NNIs of viral RdRp and showing their main interactions with residues R295 and Y674 (Carta et al., 2016). In that work, the authors identified two hydrophobic cavities HC1 (A221, I261, I287, and Y289) and HC2 (V216, Y303, V306, K307, P408, and A412) as pharmacophoric requirements. Interestingly, our compounds showed similar requirements, and while compound **1.9** interacts with residues from HC1 and HC2, the 7-chloroquinazoline derivative **1.21** moved slightly inside the binding pocket during the interaction with the RdRp structure and showed interaction with residues from HC2.

#### Antiviral Activity Against BVDV Resistant to NNI TSC

**Figure 4:**
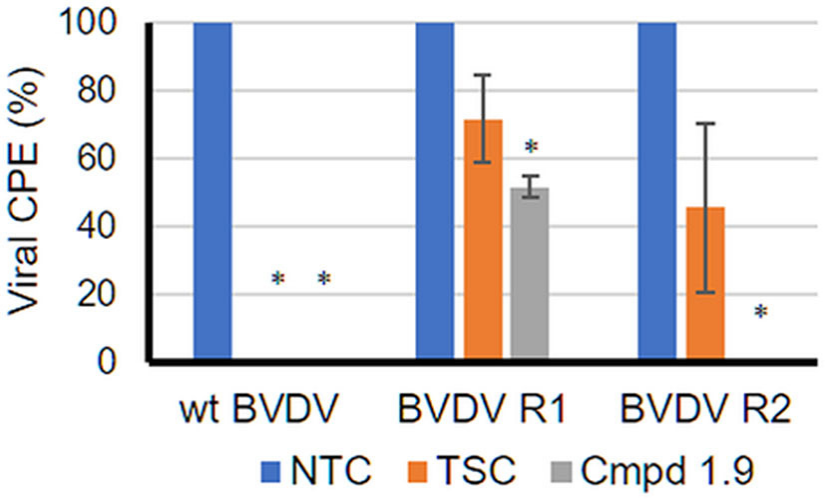
Antiviral activity of **1.9** against TSC BVDV-resistant mutants. The anti-BVDV assay was performed as stated in Materials and Methods. For infection, wtBVDV NADL and two different TSC-resistant BVDV NADLs, BVDV R1 (NS5B A392E) and BVDV R2 (NS5B N264D), were used. Compound **1.9** was used at its maximum non-cytotoxic concentration (12.5 μM). As positive control, TSC was used at 34 μM. Mock-infected cells and non-treated infected cells (NTC) were included as controls in each plate. After 3 days, cell viability was determined by the MTS/PMS method, and the percentage of the viral CPE relative to the corresponding NTC was calculated. *p < 0.05 relative to NTC.

As stated above, compound **1.9** would interact with a hydrophobic pocket other than the one identified for other BVDV RdRp NNIs such as TSC (Castro et al., 2011), suggesting that **1.9** could be active even against TSC-resistant BVDV. To test this hypothesis, we evaluated the antiviral activity of compound **1.9** against TSC-resistant BVDV (BVDV R1 and BVDV R2) (Figure 4). Interestingly, when TSC^R^-BVDV was treated with compound **1.9**, different results were observed. BVDV R1, which carries the NS5B A392E mutation, was inhibited 50% by compound **1.9**, whereas BVDV R2, which carries the N264D mutation, was completely sensitive to compound **1.9** at 12.5 μM (100% inhibition). The partial resistance observed for BVDV R1 supports RdRp as a target for compound **1.9** since the A392E mutation partially blocks its antiviral activity. On the other hand, the sensitivity observed for BVDV R2 to compound **1.9** is in agreement with our previous hypothesis and also reinforces the binding of this compound to a different hydrophobic pocket as shown by our molecular modeling studies.

**Figure 5:**
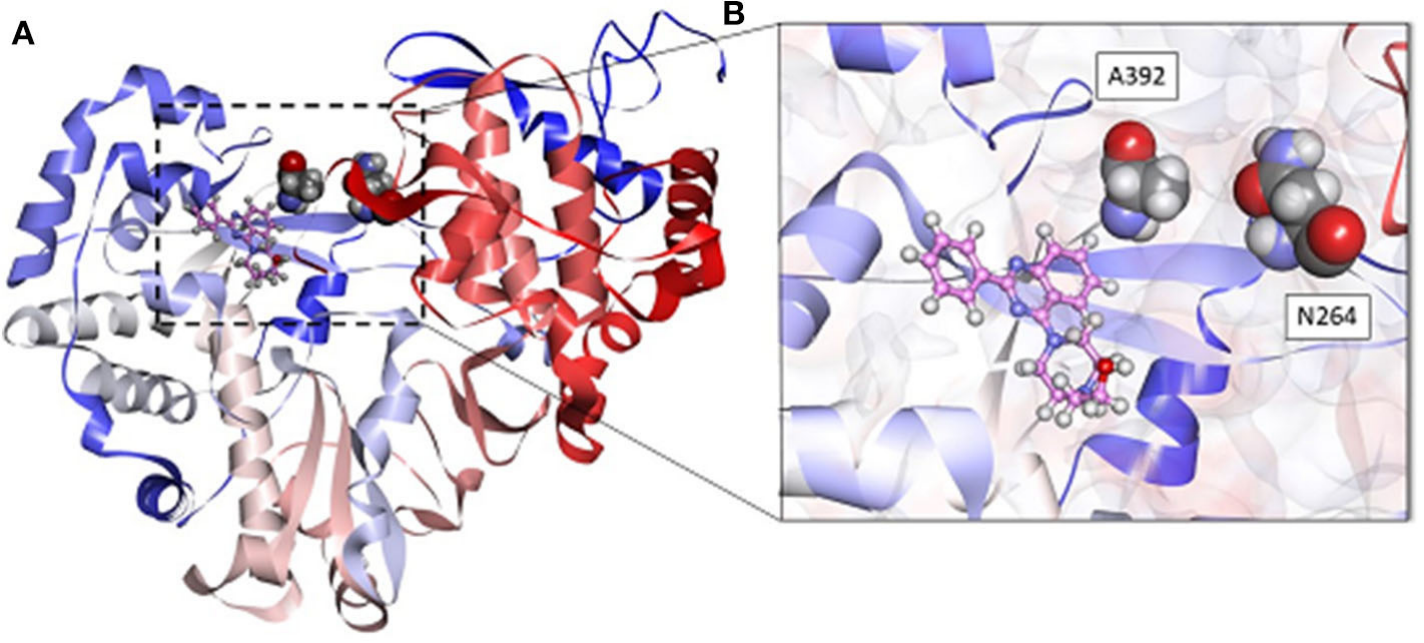
**(A)** Predicted pose of compound **1.9** (pink) within the allosteric site of the RdRp protein obtained by MD simulations. A392 and N264 are in CPK style. **(B)** Insert shows compound **1.9** located between the fingers and thumb domains of BVDV RdRp. Some amino acids were omitted for clarity.

The analysis of MD simulations demonstrated that A392 is located in the vicinity of the compound **1.9** binding site, thus explaining the partially resistant phenotype of BVDV R1 to the compound (Figure 5). In addition, this mutation was reported to disturb the local conformation of the protein. We speculate that this change would prevent the access of compounds to the predicted binding site (Tonelli et al., 2010; Castro et al., 2011).

#### Physicochemical and *in vitro* Pharmacokinetic Properties of 1.9

**Figure 6:**
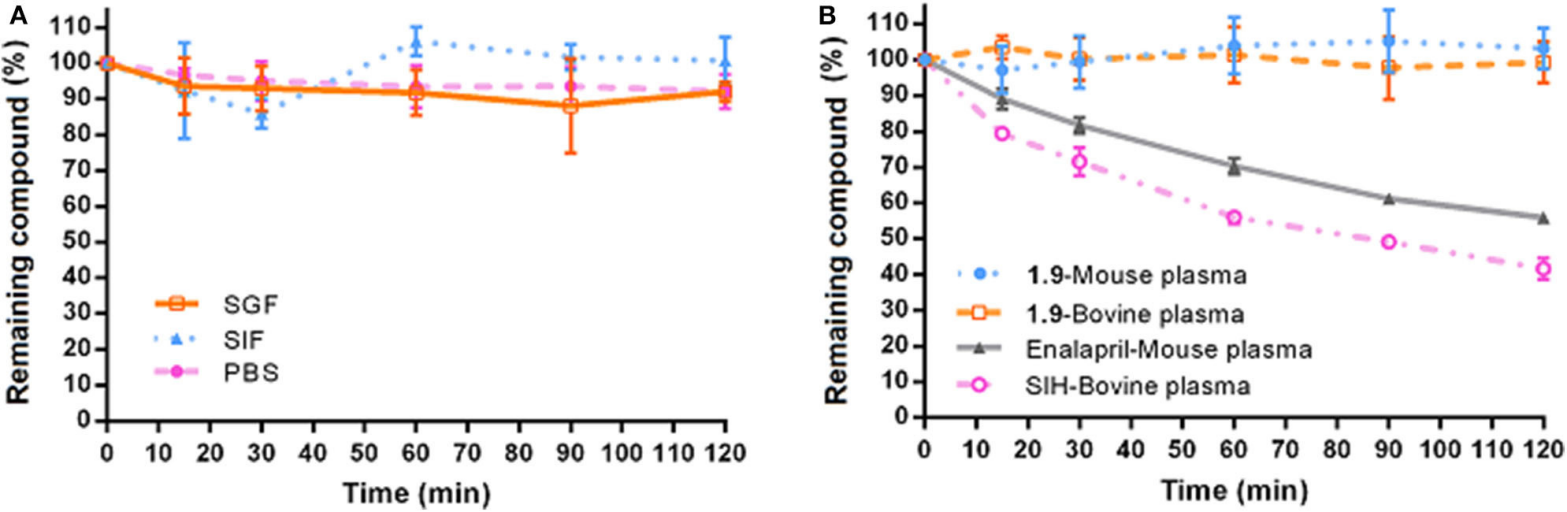
Experimental stability of compound **1.9**. **(A)** Stability profile of compound **1.9** in SGF, SIF, and PBS. **(B)** Stability profile in mouse/murine and bovine plasma samples of **1.9** and control drugs (enalapril and SIH) in mouse/murine and bovine plasma samples, respectively. Plots represent the mean percentage of compound remaining against time with the error bars representing the standard deviation of three independent experiments.

Compound **1.9** was selected to perform *in vitro* physicochemical studies (solubility and stability). The solubility (S) was evaluated at SGF (pH 1.2), SIF (pH 6.8), and PBS solution (pH 7.4), employing the shake-flask method with UV spectroscopy (Bollini et al., 2013). Compound **1.9** presented low solubility values (Table 2), which were within the range normally observed for oral drugs (http://pharmeuropa.edqm.eu/home/). The stability was evaluated by calculating the percentage of remaining drug (HPLC–MS) after contact with different incubation media (SIF, SGF, PBS, mouse, and bovine plasma) for 120 min. Figure 6 shows the high-stability profile of **1.9** under all the conditions employed. Neither compound modification nor degradation was detected after the different incubation periods. The stability profiles of enalapril and SIH, used as positive controls for mouse and bovine plasma, respectively, are also shown in Figure 5B. Additionally, no significant differences were found in the concentration of compound **1.9** between mouse and bovine plasma samples.

**Table 2:** Experimental solubility and stability data for compound **1.9**.

	**Solubility (mg/ml)^a^^,^^b^**			**Stability**				
				**(t_1/2_** **min)^b^**				
**Compd**.	**SGF**	**SIF**	**PBS**	**SGF**	**SIF**	**PBS**	**Mouse/murine plasma**	**Bovine plasma**
**1.9**	8.7 ± 0.4	0.4 ± 0.2	0.3 ± 0.1	>120	>120	>120	>120	>120

a *Verapamil HCl was used as internal control: Solubility (mg/ml)_PBS_ 0.71 ± 0.04. Literature data: 0.58 mg/ml (Timmins et al., 1997)*.

b *Values are expressed as the mean ± standard deviation of three independent experiments run in triplicate. European Pharmacopeia solubility definitions: 33–100 mg/ml (soluble), 1–10 mg/ml (slightly soluble), 0.1–1 mg/ml (very slightly soluble), <0.1 mg/ml (insoluble) (http://pharmeuropa.edqm.eu/home/). SGF, simulated gastric fluid (pH 1.2); SIF, simulated intestinal fluid (pH 6.8); PBS, phosphate-buffered saline solution (pH 7.4)*.

## Conclusions

From a series of 24 derivatives of *N*-(2-morpholinoethyl)-2-phenylquinazolin-4-amine (**1**), eight compounds showed improved anti-BVDV activity. A molecular modeling analysis of the most active compound **1.9** showed that this compound would bind to a hydrophobic pocket of the BVDV RdRp that is different from the one previously described for most BVDV RdRp NNIs. Our results indicate that compound **1.9** inhibits the replication of TSC-resistant BVDV variants *in vitro*, which carry a mutation in the viral polymerase that is selected by almost all the NNIs described for BVDV (N264D). Taking into account the main residues with which compound **1.9** would interact, its binding to BVDV RdRp could affect the template channel, through the interaction with both the fingers domain (motif II) and the β-thumb region, and prevent the synthesis of viral RNA. In addition, compound **1.9** presented adequate solubility in different media and a high-stability profile in murine and bovine plasma samples. These parameters are important for the development of oral drugs. Taken together, these results suggest that compound **1.9** is an NNI of BVDV that can be considered a promising candidate to treat BVDV infection and encourages further studies assessing the mechanism of action and potential combination with other previously reported antivirals.

## Data Availability Statement

The original contributions presented in the study are included in the article/Supplementary Materials, further inquiries can be directed to the corresponding authors.

## Author Contributions

MB and LC designed and supervised the study. NA, LB, and MB performed the docking and computational simulations. GF, AB, and MB conducted chemical synthesis. EC, RR, ME, and MF performed biological experiments. DF performed the experimental stability study of compound **1.9**, and GF performed solubility assays. All authors were involved in the preparation of the manuscript and approved the final version.

## Conflict of Interest

The authors declare that the research was conducted in the absence of any commercial or financial relationships that could be construed as a potential conflict of interest.
