# Supplementary material for: Design and Optimization of Quinazoline Derivatives: New Non-nucleoside Inhibitors of Bovine Viral Diarrhea Virus
Source: Front Chem. 2020 Dec 10;8:590235. doi: 10.3389/fchem.2020.590235 (PMC7793975; doi:10.3389/fchem.2020.590235)

Supplementary Material

Design and optimization of quinazoline derivatives: new non-nucleoside inhibitors of bovine viral diarrhea virus.

Gabriela A. Fernández[1, †], Eliana F. Castro[2,5 †], Rocío A. Rosas[3,4], Daniela M. Fidalgo[1], Natalia S. Adler[1], Leandro Battini[1], Maria J. España de Marco[5], Matias Fabiani[3,4], Ana M. Bruno[6], Mariela Bollini[1,*], Lucia V. Cavallaro[3,*]

Contents

Molecular modeling **S2**

Drug solubility testing **S6**

DMSO and water/DMSO (98:2) stability **S6**

Synthesis of 4a-g and 5a-g compounds **S7**

NMR Spectra **S10**

Molecular modeling

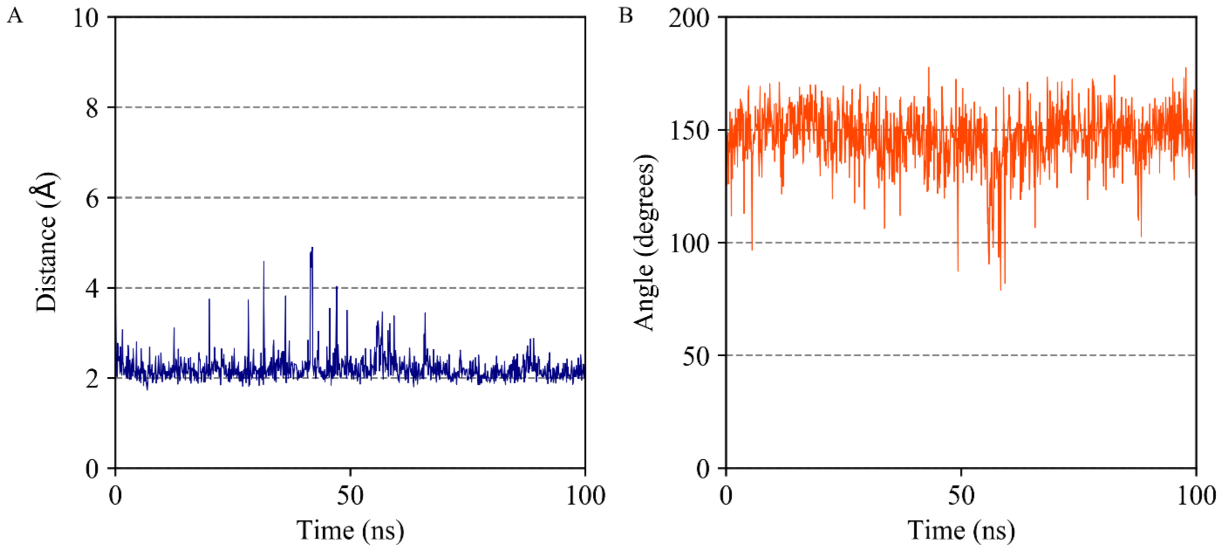

Supplementary Figure 1. Time dependence of hydrogen bond distance (A) and angle (B) between the HN atoms of compound **1.9** and the carbonyl O of Arg295.

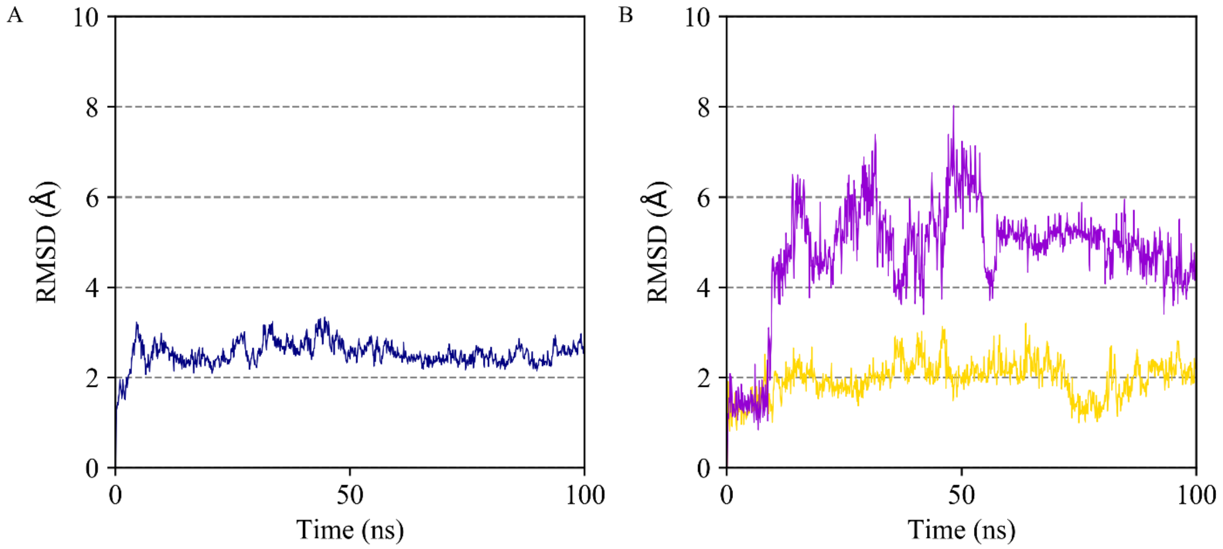

Supplementary Figure 2. RMSD as a function of time for (A) protein backbone atoms, and (B) compounds **1.9** (violet) and **1.21** (yellow).

Active compounds

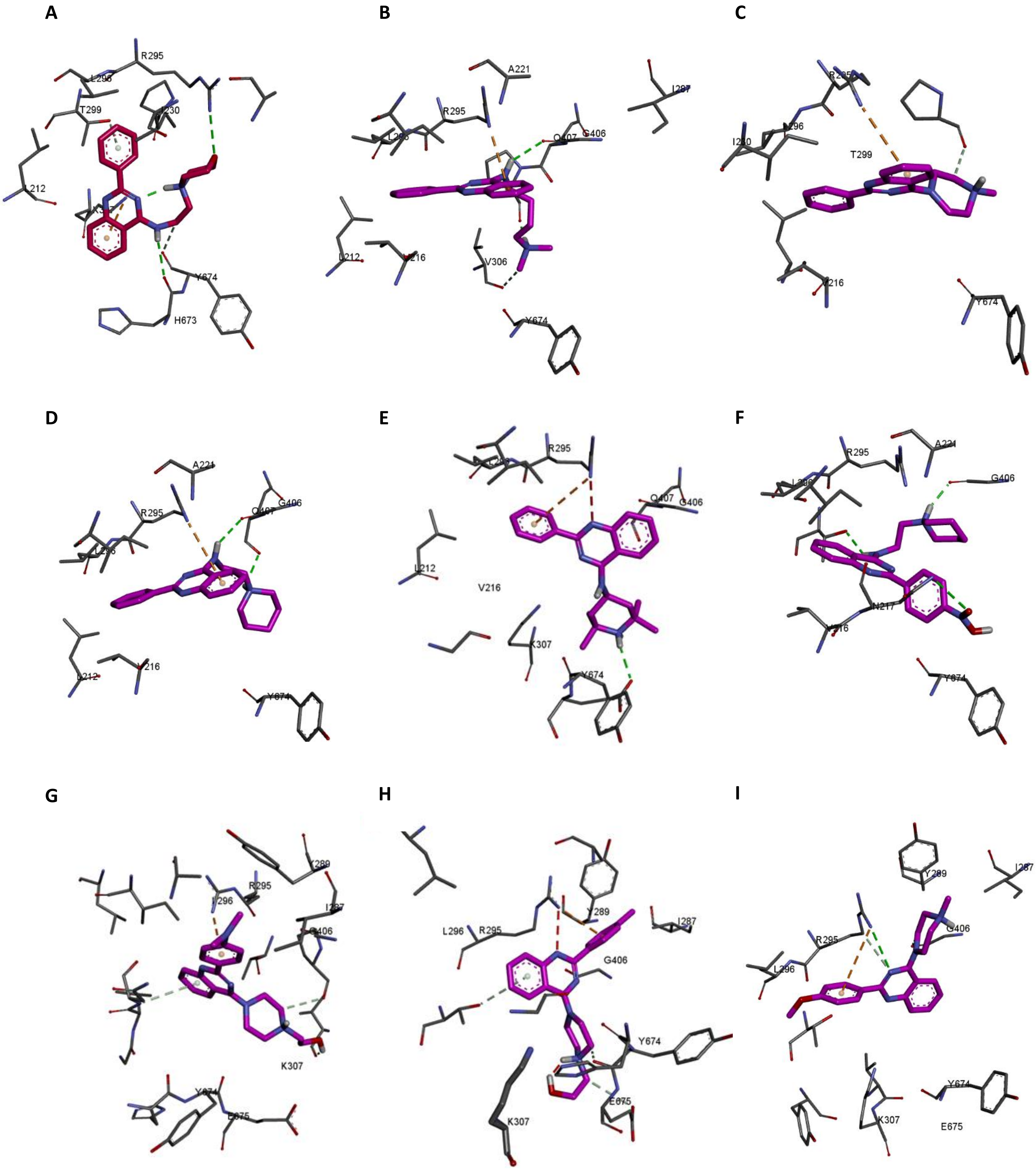

J

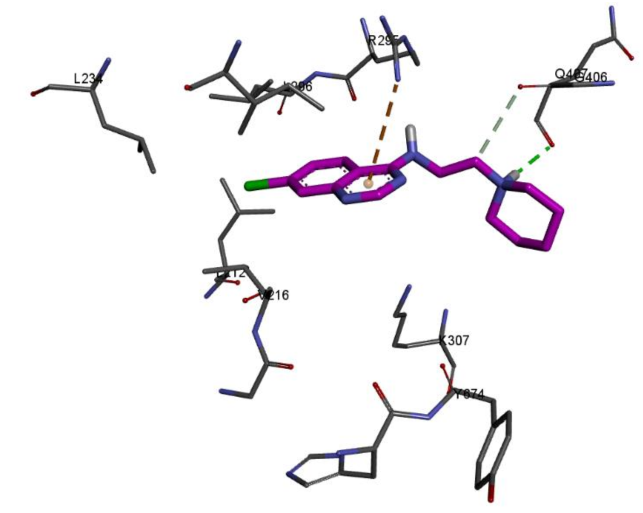

K

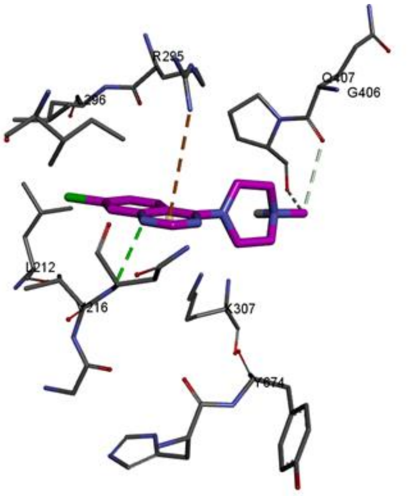

Inactive compounds

L

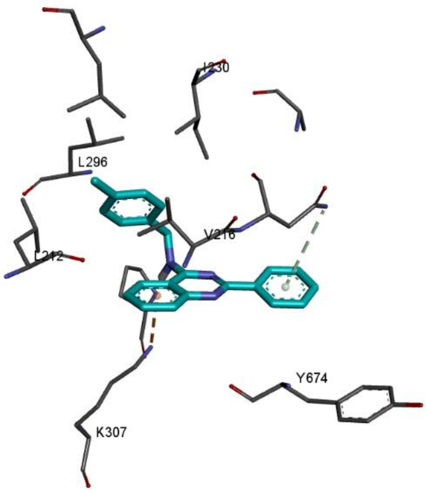

M

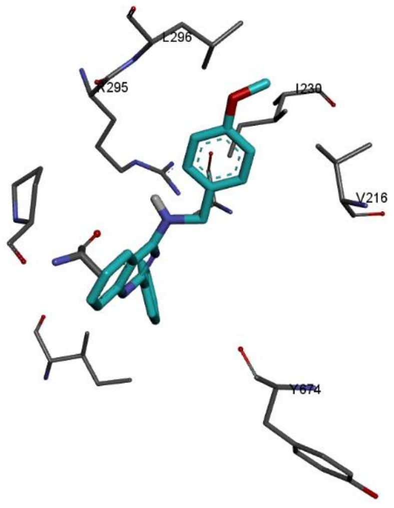

N

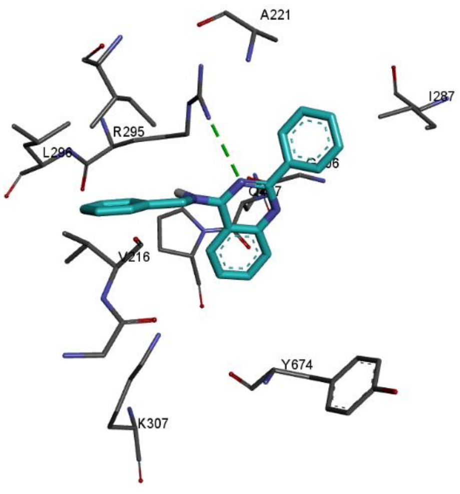

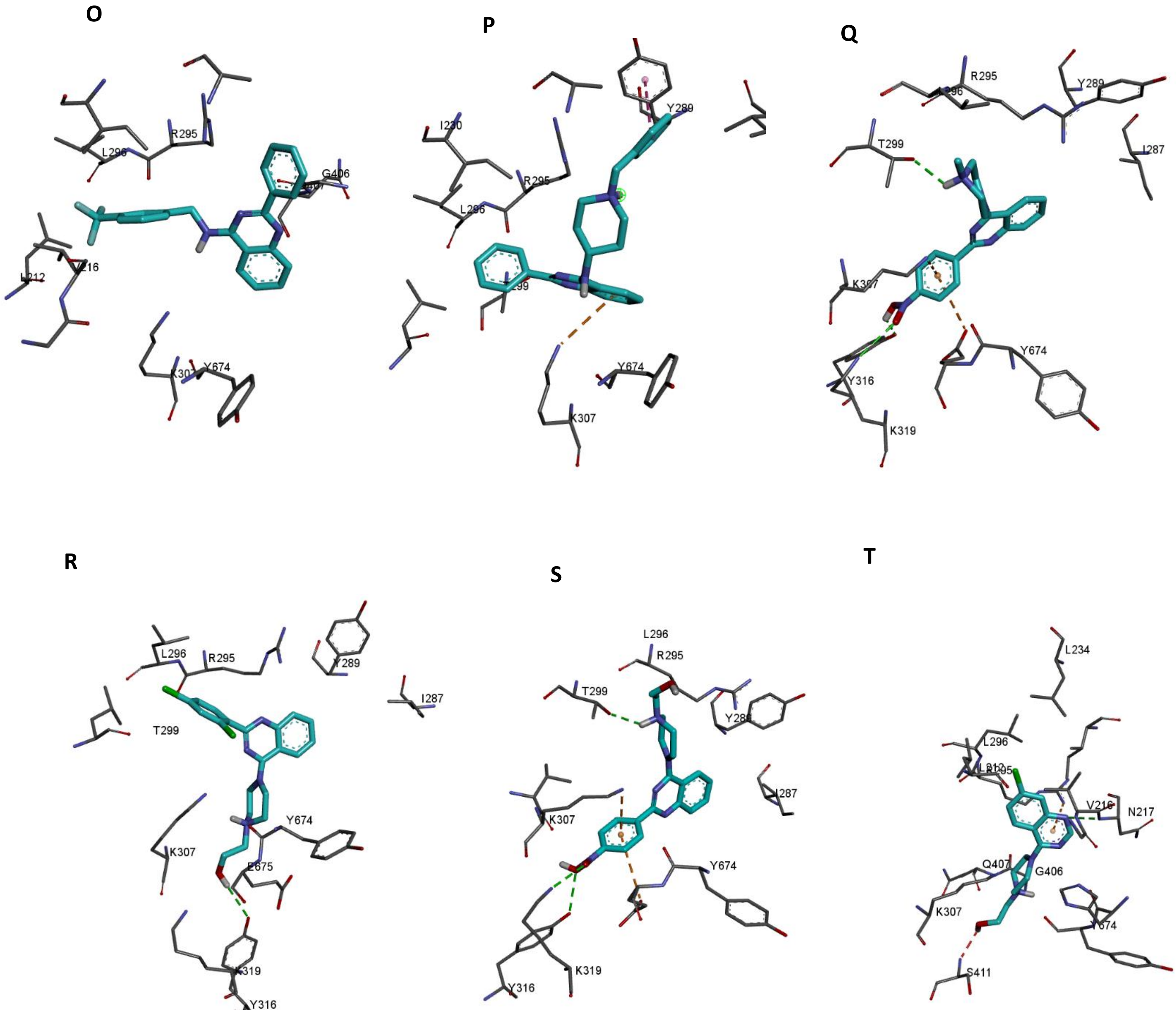

Supplementary Figure 3. Predicted interactions of active and inactive compounds within the allosteric site of RdRp protein obtained by molecular dynamics simulations. Active compounds A) 1; B) 1.7; C) 1.8; D) 1.10; E) 1.12; F) 1.15; G) 1.18; H) 1.19; I) 1.20; J) 1.23; K) 1.24. Inactive compounds: L) 1.2; M) 1.3; N) 1.4; O) 1.5; P) 1.11; Q) 1.14; R) 1.16; S) 1.17.

Drug solubility testing

UV-Visible spectrums were measured with a UV3600 UV-Vis-NIR spectrophotometer in a range of 500-200 nm using a quartz cuvette. The measurements were determined at **λ** values corresponding to the maximum of absorption. Baselines were determined with MeOH:media (1:1) mixture. Sample concentrations were determined using maximum absorption values. All compounds were tested in triplicate.

Preparation of calibration curve

Compound **1.9** (1.0 mg) was diluted in 1 mL of DMSO. From this solution, different volumes were combined with MeOH:media (1:1) mixture to obtain concentrations between 2.0 – 40.0 µg/mL.

Preparation of samples

Compound **1.9** (1.0 mg) was diluted in 1 mL of simulated gastric fluid (SGF, pH 1.2), simulated intestinal fluid (SIF, pH 6.8) or phosphate buffered saline solution (PBS, pH 7.4) was added. The vial contents were mixed at 1500 rpm at 37 °C for 24 h and then incubated at 37 °C for 24 h without stirring. Samples were filtered (0.2 µm, Sterile Acrodisc® 13, GelmanScience) before analysis. The filtered solutions were diluted in MeOH:media (1:1) mixture to achieved final concentrations of 0.01 (SGF), 0.02 (SIF) and 0.04 µg/mL (PBS). Verapamil and procaine were used as internal controls.

DMSO and water/DMSO (98:2) stability

The stability of compound **1.9** was determined by UV-Vis spectrophotometry. UV-Vis spectrums were measured in a range of 500-200 nm using a quartz cuvette and the absorption was determined at 331 nm. Samples with 1.0 mg of **1.9** in 1 mL of DMSO or water/DMSO (98:2) mixture were prepared, and they were kept at room temperature without stirring. Independent samples were measured every 1 week over 5 weeks. For the measurements, solutions were diluted at 1/100 in DMSO and 1/50 in water:DMSO (98:2) mixture. The values in the chart are the mean of three independent samples. Compound **1.9** was stable in both media at least for 5 weeks.

Synthesis of *quinazoline* intermediates (4a-g and **5a-g**)

Synthesis of 4-aminoquinazoline derivates

a) *2-aminobenzamide* (**3**). To a suspension of isatoic anhydride (**2**, 1.0 g, 6.1 mmol) and triethylamine (0.62 g, 6.1 mmol, 0.86 mL) in 20 mL in acetonitrile:water (3:1) mixture, concentrated aqueous solution of ammonia (0.21 g, 6.1 mmol, 0.7 mL) was added portionwise. The mixture was stirred over 2 h at rt and extracted with AcOEt. The combined organic layers were washed with water and brine, dried over anh. $MgSO_4$, filtered and concentrated to yield the desired amide (0.44 g, 3.2 mmol, 53 %).[1]

Synthesis of quinazolinone scaffolds (**4a-g**).

b) *2-phenylquinazolin-4(3H)-one derivates* (**4a-f**). Sodium hydrogensulfite (78 mg, 0.73 mmol) was added to a solution of 2-aminobenzamide (100 mg, 0.73 mmol) and the corresponding benzaldehyde (0.73 mmol, for **4a**: benzaldehyde; **4b**: 4-nitrobenzaldehyde; **4c**: 2,4-dichlorobenzaldehyde; **4d**: 4-(dimethylamino)benzaldehyde; **4e**: 4-methoxybenzaldehyde, **4f**: 4-methylbenzaldehyde) in *N,N*-dimethylacetamide (2 mL). The mixture was heated with stirring at 150 °C over 2 h and poured into ice water (200 mL). The precipitate was collected, washed with water, and dried in vacuo. The compounds were purified by recrystallization from EtOH to afford the products[2].

2-phenylquinazolin-4(3H)-one (**4a**). White solid (81 mg, 0.27 mmol, 76 %). ^{1}H NMR (600 MHz, $CDCl_3$) δ 10.35 (br s, 1H), 8.33 (d, J = 7.9 Hz, 1H), 8.14 (d, J = 7.6 Hz, 2H), 7.87 (d, J = 8.1 Hz, 1H), 7.81 (t, J = 7.6 Hz, 1H), 7.59 (d, J = 6.6 Hz, 3H), 7.52 (t, J = 7.5 Hz, 1H).

2-(4-nitrophenyl)quinazolin-4(3H)-one (**4b**). Yellow solid (126 mg, 0.47 mmol, 43 %, from 150 mg of **3**). ^{1}H NMR (600 MHz, DMSO-d_6) δ 12.84 (s, 1H), 8.19 (dd, J = 7.9, 1.5 Hz, 1H), 7.88 (ddd, J = 8.5, 7.1, 1.6 Hz, 1H), 7.80 (dd, J = 8.2, 1.0 Hz, 1H), 7.58 (ddd, J = 8.1, 7.1, 1.2 Hz, 1H).

2-(2,4-dichlorophenyl)quinazolin-4(3H)-one (**4c**). Yellow solid (100 mg, 0.34 mmol, 47 %). ^{1}H NMR (600 MHz, $CDCl_3$) δ 10.06 (s, 1H), 8.30 (ddd, J = 8.0, 1.5, 0.7 Hz, 1H), 7.84 – 7.81 (m, 3H), 7.57 – 7.54 (m, 2H), 7.45 (dd, J = 8.3, 2.0 Hz, 1H).

2-[(4-dimethylamino)phenyl]quinazolin-4(3H)-one (**4d**). Yellow solid (132 mg, 0.50 mmol, 68 %). ^{1}H NMR (600 MHz, $CDCl_3$) δ 10.04 (br s, 1H), 8.31 – 8.26 (m, 1H), 8.01 (d, J = 8.7 Hz, 2H), 7.78 – 7.71 (m, 2H), 7.42 (ddd, J = 8.0, 5.9, 2.2 Hz, 1H), 6.79 (d, J = 8.8 Hz, 2H), 3.08 (s, 6H).

---

[1] Cheng, R., Guo, T., Zhang-Negrerie, D., Du, Y., Zhao, K. (2013). One-pot synthesis of quinazolinones from anthranilamides and aldehydes via p-toluenesulfonic acid catalyzed cyclocondensation and phenyliodine diacetate mediated oxidative dehydrogenation. Synthesis (Germany) 45 (21):2998-3006. doi: 10.1055/s-0033-1338521.

[2] Hour, M.-J., Huang, L.-J., Kuo, S.-C., Xia, Y., Bastow, K., Nakanishi, Y., Hamel, E., Lee, K.-H. (2000). 6-Alkylamino- and 2,3-dihydro-3‘-methoxy-2-phenyl-4-quinazolinones and related compounds: their synthesis, cytotoxicity, and inhibition of tubulin polymerization. J. Med. Chem. 43:4479-4487.

2-(4-methoxyphenyl)quinazolin-4(3H)-one (**4e**). White solid (184 mg, 0.73 mmol, 100 %). ^{1}H NMR (500 MHz, $CDCl_3$) δ 10.75 (s, 1H), 8.31 (ddd, *J* = 8.0, 1.5, 0.7 Hz, 1H), 8.19 – 8.13 (m, 2H), 7.87-7.82 (m, 1H), 7.79 (ddd, *J* = 8.2, 7.0, 1.6 Hz, 1H), 7.48 (ddd, *J* = 8.1, 7.0, 1.3 Hz, 1H), 7.11 – 7.06 (m, 2H), 3.92 (s, 3H).

2-(p-tolyl)quinazolin-4(3H)-one (**4f**). White solid (184 mg, 0.73 mmol, 100 %). ^{1}H NMR (600 MHz, DMSO-d_6) δ 12.47 (s, 1H), 8.14 (dd, *J* = 7.9, 1.6 Hz, 1H), 8.12 – 8.08 (m, 2H), 7.83 (ddd, *J* = 8.4, 7.1, 1.6 Hz, 1H), 7.73 (dd, *J* = 8.3, 1.1 Hz, 1H), 7.51 (ddd, *J* = 8.1, 7.1, 1.2 Hz, 1H), 7.36 (d, *J* = 8.0 Hz, 2H), 2.39 (s, 3H).

c) *7-chloroquinazolin-4(3H)-one* (**4g**). 2-amino-4-chlorobenzoic acid (**6**, 2.0 g, 11 mmol) and formamide (3.96 g, 88 mmol) were heated at 150 ºC. The reaction progress was monitored by TLC. After reaction was complete, the mixture was allowed to cold to room temperature. Water was added and a precipitate was filtered off. The solid was recrystallized from acetonitrile to give the product. White solid (1.7 g, 8.8 mmol, 80 %). Compound was used for the next step without further characterization.

Synthesis of 4-chloroquinazoline derivates (**5a-g**).

d) *4-chloro-2-phenylquinazoline derivates* (**5a-f**). Phosphorus oxychloride (1 mL, 10.7 mmol) was added to quinazolinones **4a-f** (100 mg), and the solution was stirred for 2 h under reflux. For **5c**, phosphorous pentachloride (1.2 eq.) was added. The reaction mixture was evaporated in vacuo, washed with toluene (3 x 5 mL), and the dry residue was dissolved in chloroform. The chloroformic solution was poured on an ice bath, concentrated aqueous ammonia was added to adjust the pH to 10. The mixture was partitioned and the combined chloroform layers were washed with water. The organic layer was dried over anh. Na_2SO_4 and evaporated in vacuo. Compounds **5a**, **5d-f** were used without further purification due to they showed a single spot on TLC plate, while **5b** was purified by chromatography column in dichloromethane/methanol (99:1) and **5c** with cyclohexane/ethyl acetate (95:5 to 90:10) as mobile phase.[3]

4-chloro-2-phenylquinazoline (**5a**). White solid (0.081 mg, 0.27 mmol, 76 %). ^{1}H NMR (600 MHz, $CDCl_3$) δ 8.60 (dd, *J* = 6.4, 2.8 Hz, 2H), 8.27 (d, *J* = 8.3 Hz, 1H), 8.12 (d, *J* = 8.4 Hz, 1H), 7.96-7.92 (m, 1H), 7.68 (t, 1H), 7.55 – 7.50 (m, 3H).

4-chloro-2-(4-nitrophenyl)quinazoline (**5b**). White solid (61.2 mg, 0.21 mmol, 48 %, from 120 mg of **4b**). ^{1}H NMR (500 MHz, $CDCl_3$) δ 8.82 – 8.76 (m, 2H), 8.40 – 8.34 (m, 2H), 8.31 (ddd, *J* = 8.4, 1.4, 0.6 Hz, 1H), 8.15 (ddd, *J* = 8.5, 1.2, 0.7 Hz, 1H), 8.01 (ddd, *J* = 8.4, 7.0, 1.4 Hz, 1H), 7.76 (ddd, *J* = 8.3, 7.0, 1.2 Hz, 1H).

4-chloro-2-(2,4-dichlorophenyl)quinazoline (**5c**). Yellow solid (76.0 mg, 0.24 mmol, 70 %). ^{1}H NMR (600 MHz, $CDCl_3$) δ 8.33 (dd, *J* = 8.4, 1.4 Hz, 1H), 8.14 (d, *J* = 8.4 Hz, 1H), 8.01 (ddd, *J* =

[3] a) Nakamoto, K. *et al*. (2011). Antifungal agent containing heterocyclic compound. U.S. Patent No 7,932,272 B2. Washington, DC: U.S. Patent and Trademark Office. b) Arnott, A., Chan, L. C., Cox, B. G., Meyrick, B., Phillips, A. (2011). $POCl_3$ chlorination of 4-quinazolones. J. Org. Chem. 76:1653-1661. doi: 10.1021/jo102262k.

8.5, 7.0, 1.4 Hz, 1H), 7.86 (d, *J* = 8.3 Hz, 1H), 7.78 (ddd, *J* = 8.2, 7.0, 1.2 Hz, 1H), 7.56 (d, *J* = 2.0 Hz, 1H), 7.40 (dd, *J* = 8.3, 2.0 Hz, 1H).

4-chloro-2-[(4-dimethylamino)phenyl]quinazoline (**5d**). Reddish brown solid (106 mg, 0.37 mmol, 99 %). ^{1}H NMR (600 MHz, $CDCl_3$) δ 8.50 – 8.46 (m, 2H), 8.18 (dd, *J* = 8.3, 1.3 Hz, 1H), 8.01 (d, *J* = 8.5 Hz, 1H), 7.86 (ddd, *J* = 8.4, 6.9, 1.4 Hz, 1H), 7.55 (ddd, *J* = 8.1, 7.0, 1.0 Hz, 1H), 6.81 (d, *J* = 8.6 Hz, 2H), 3.08 (s, 6H).

4-chloro-2-(4-methoxyphenyl)quinazoline (**5e**). Yellow solid (95.0 mg, 0.35 mmol, 88 %). ^{1}H NMR (600 MHz, $CDCl_3$) δ 8.59 – 8.54 (m, 2H), 8.25 – 8.21 (m, 1H), 8.09 (d, *J* = 8.4 Hz, 1H), 7.92 (ddd, *J* = 8.4, 6.9, 1.4 Hz, 1H), 7.63 (ddd, *J* = 8.2, 6.9, 1.1 Hz, 1H), 7.05 – 7.02 (m, 2H), 3.91 (s, 3H).

4-chloro-2-(p-tolyl)quinazoline (**5f**). Brown solid (86.9 mg, 0.34 mmol, 80 %, from 0.43 mmol of **4f**). ^{1}H NMR (600 MHz, $CDCl_3$) δ 8.51 – 8.45 (m, 2H), 8.25 (ddd, *J* = 8.4, 1.4, 0.7 Hz, 1H), 8.08 (dt, *J* = 8.4, 0.9 Hz, 1H), 7.94 – 7.90 (m, 1H), 7.65 (ddd, *J* = 8.2, 6.9, 1.2 Hz, 1H), 7.35 – 7.31 (m, 2H), 2.45 (s, 3H).

e) 4,7-dichloroquinazoline (**5g**). 7-chloroquinazolin-4(3H)-one (**4g**) (0.80 g, 4.4 mmol) and an excess of thionyl chloride (15 mL) were heated at reflux over 2 h. The excess of chloride was distillated and the mixture was washed with small portions of toluene. Dichloromethane was added and the mixture was partitioned. The organic layer was washed with a solution of $NaHCO_3$ 5 %. The organic phase was dried over anh. $MgSO_4$, filtered and concentrated in vacuo. The product was obtained after purification with chromatography column in dichloromethane:methanol (90:10) (83 %, 0.72 g). ^{1}H NMR (500 MHz, DMSO-d_6) δ 8.40 (s, 1H), 8.13 (d, *J* = 8.6 Hz, 1H), 7.79 (d, *J* = 2.1 Hz, 1H), 7.60 (dd, *J* = 8.5, 2.1 Hz, 1H).

^{1}H y ^{13}C-RMN Spectra

2-phenylquinazolin-4(3H)-one (**4a**)

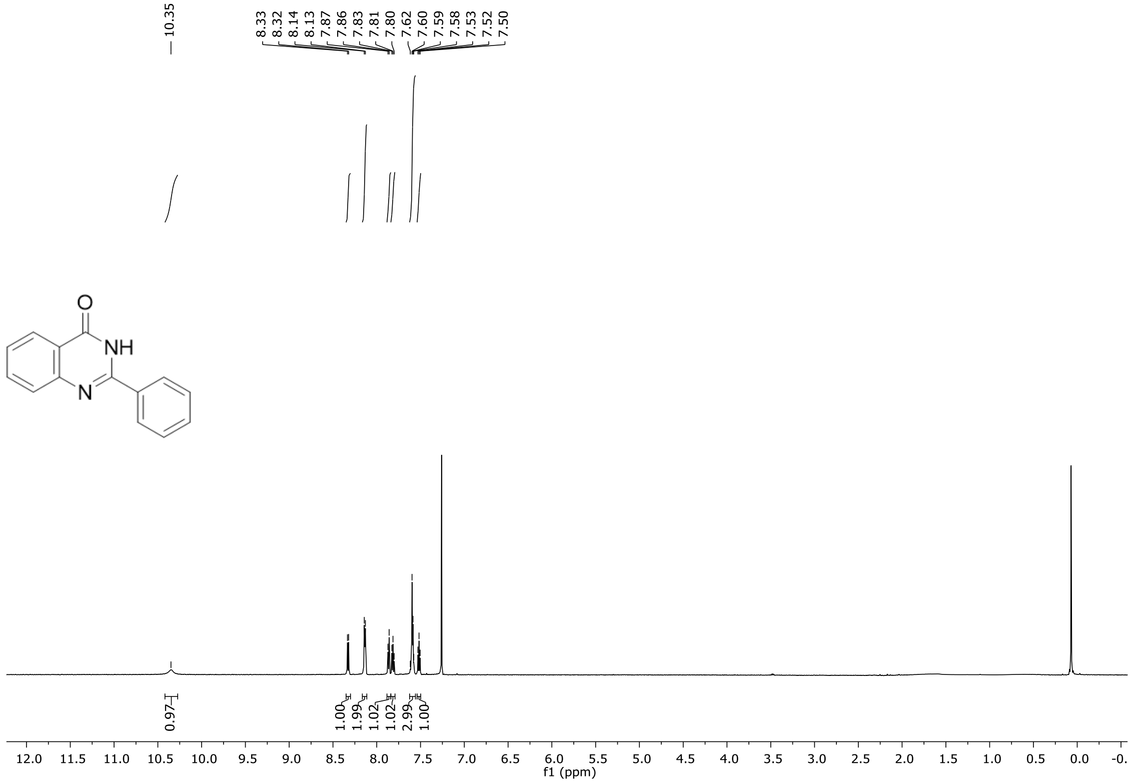

2-(4-nitrophenyl)quinazolin-4(3H)-one (**4b**)

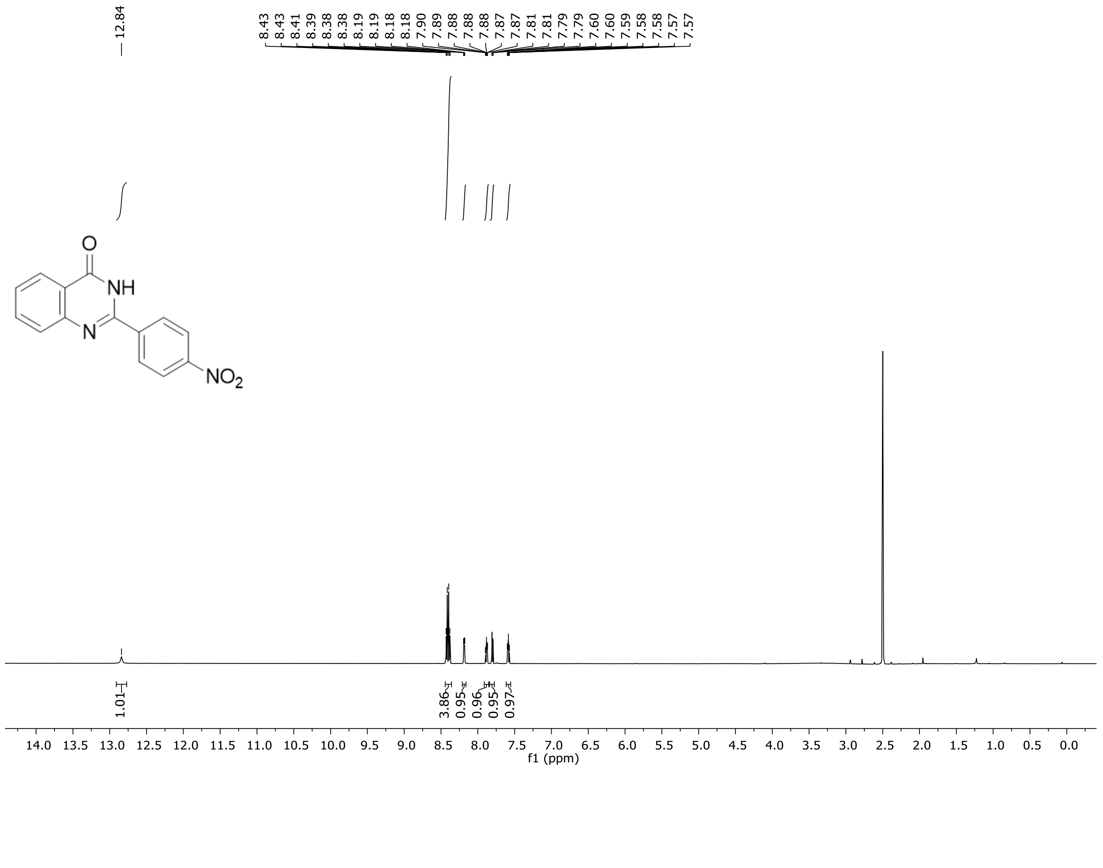

2-(2,4-dichlorophenyl)quinazolin-4(3H)-one (**4c**)

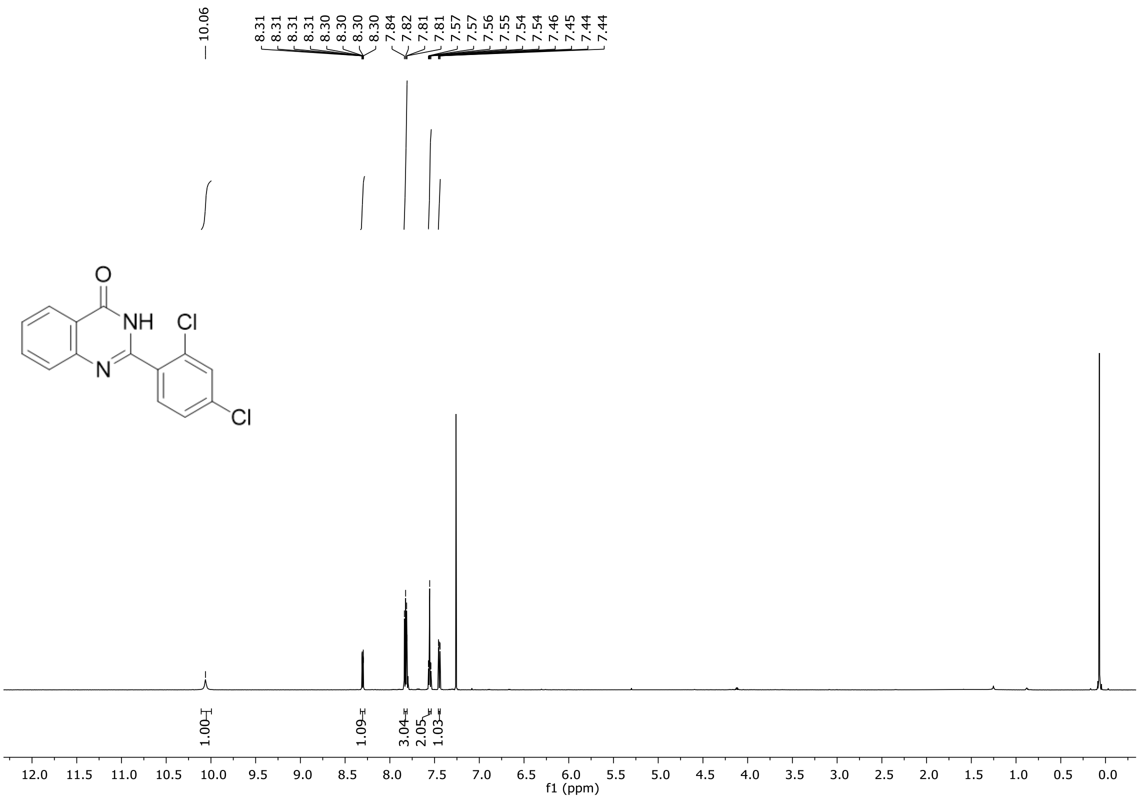

2-[(4-dimethylamino)phenyl]quinazolin-4(3H)-one (**4d**)

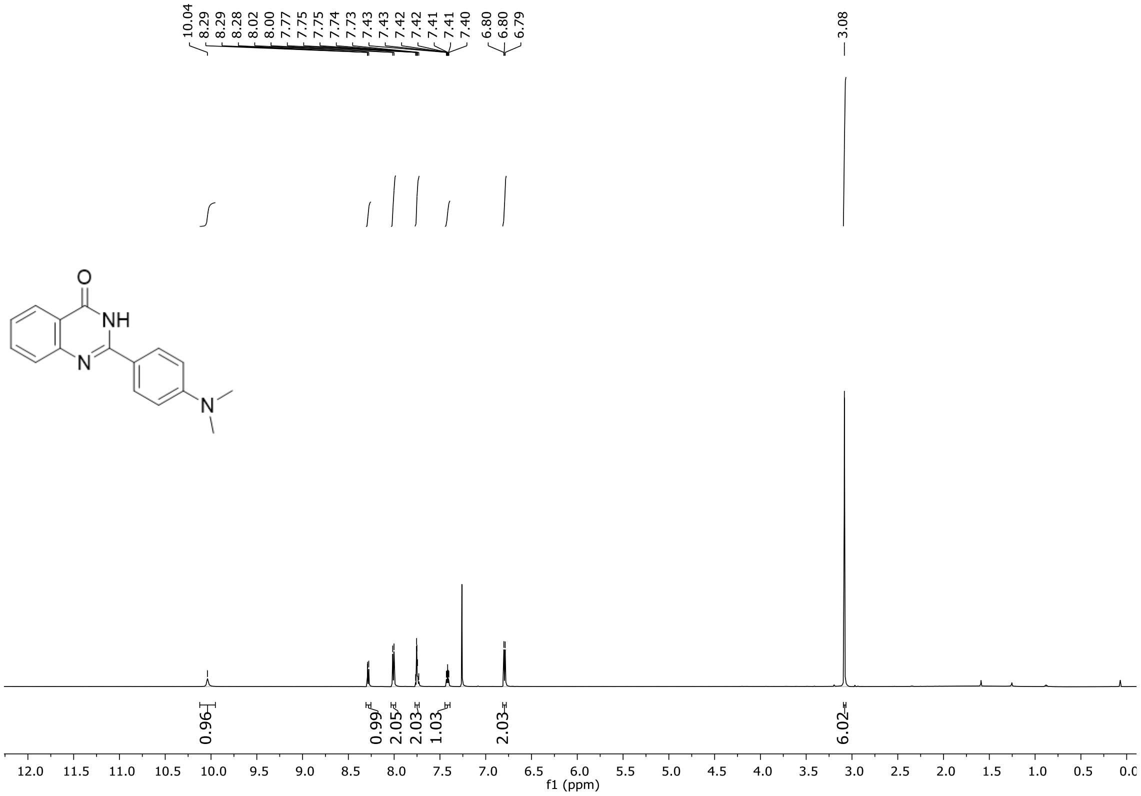

2-(4-methoxyphenyl)quinazolin-4(3H)-one (**4e**)

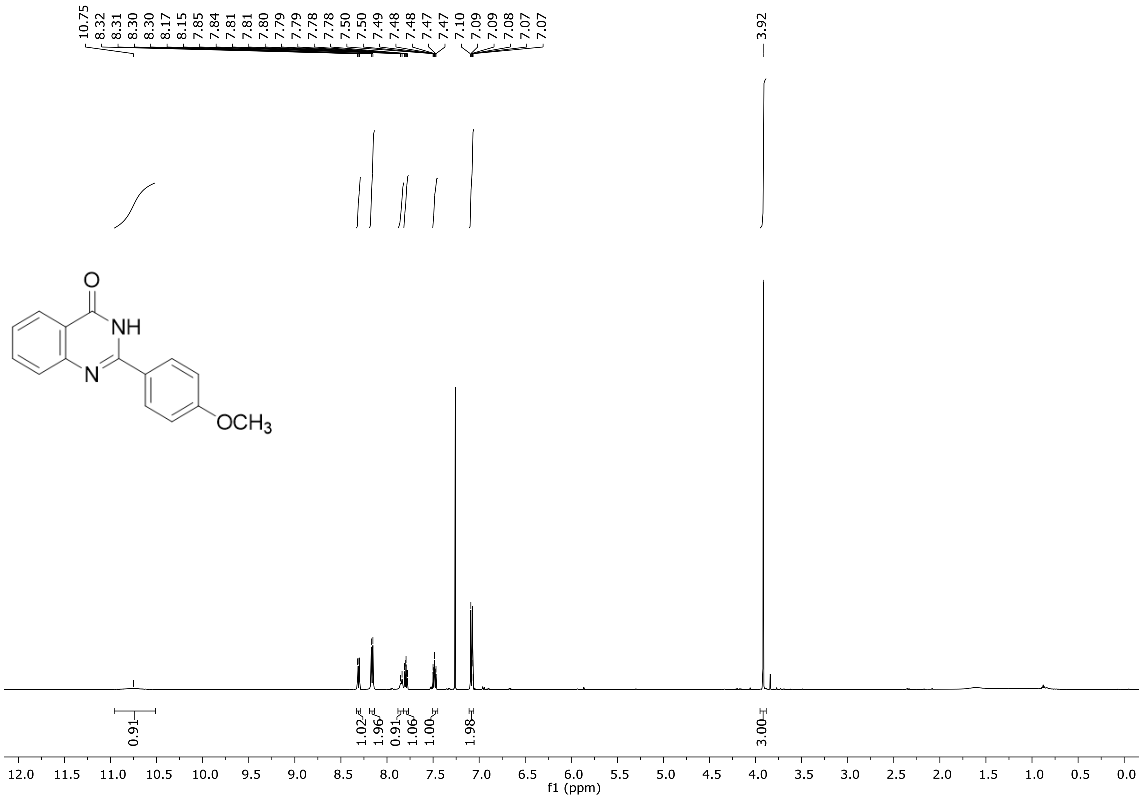

2-(p-tolyl)quinazolin-4(3H)-one (**4f**)

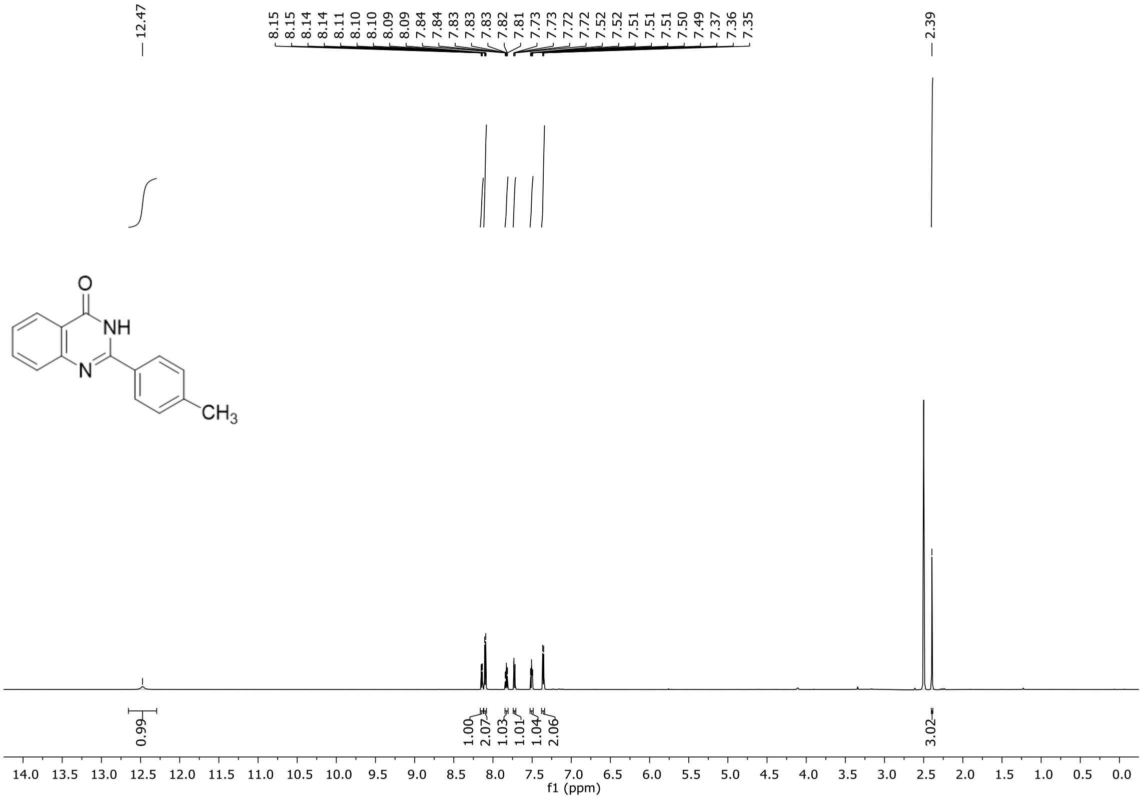

4-chloro-2-phenylquinazoline (**5a**)

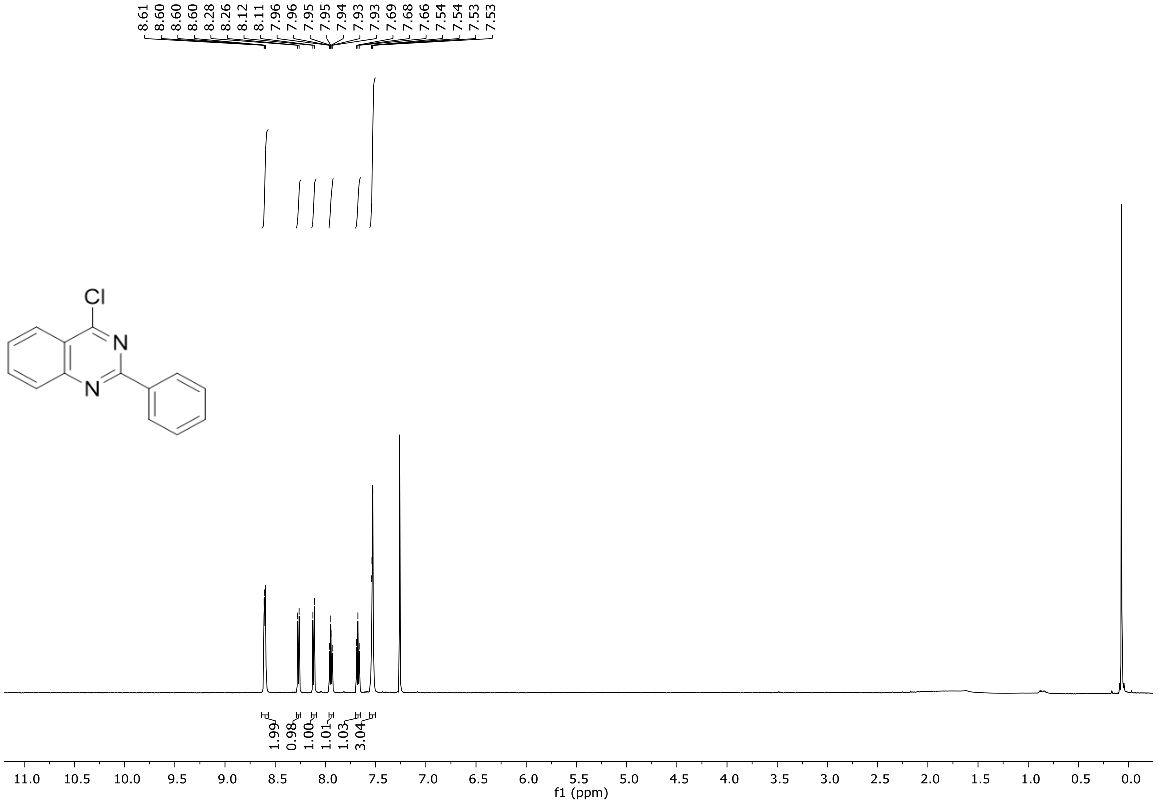

4-chloro-2-(4-nitrophenyl)quinazoline (**5b**)

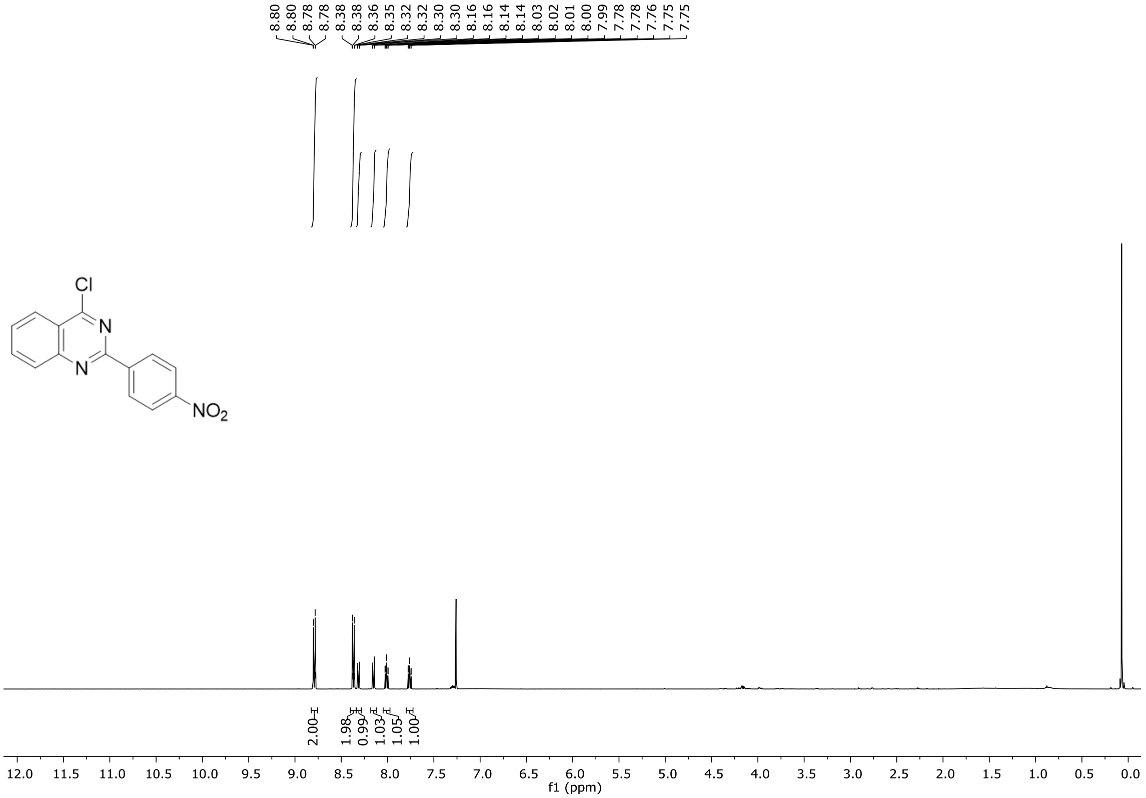

4-chloro-2-(2,4-dichlorophenyl)quinazoline (**5c**)

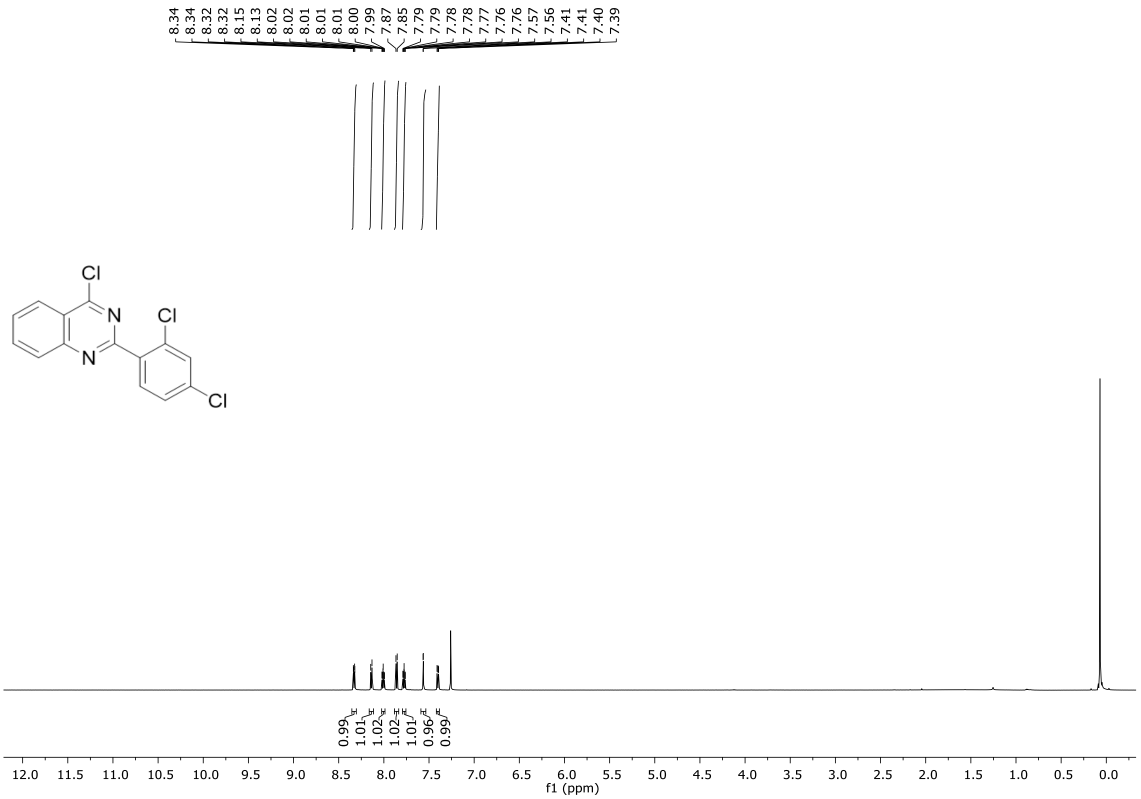

4-chloro-2-[(4-dimethylamino)phenyl]quinazoline (**5d)**

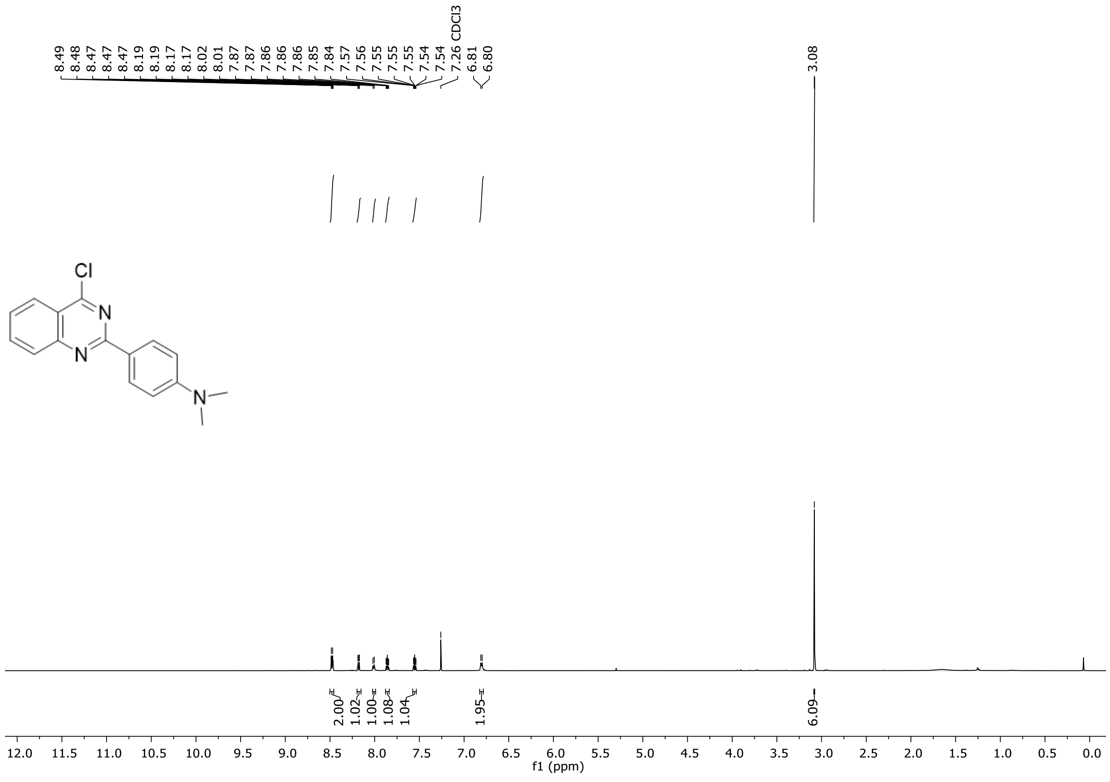

4-chloro-2-(4-methoxyphenyl)quinazoline (**5e**)

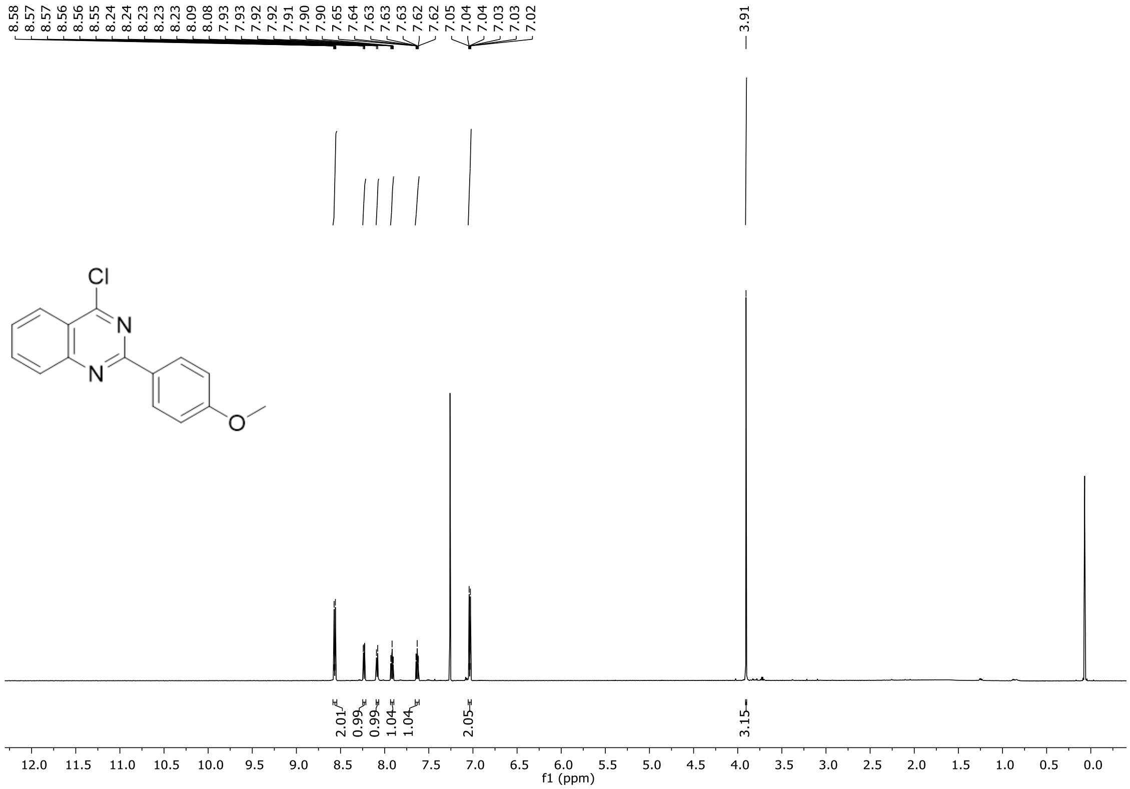

4-chloro-2-(p-tolyl)quinazoline (**5f**)

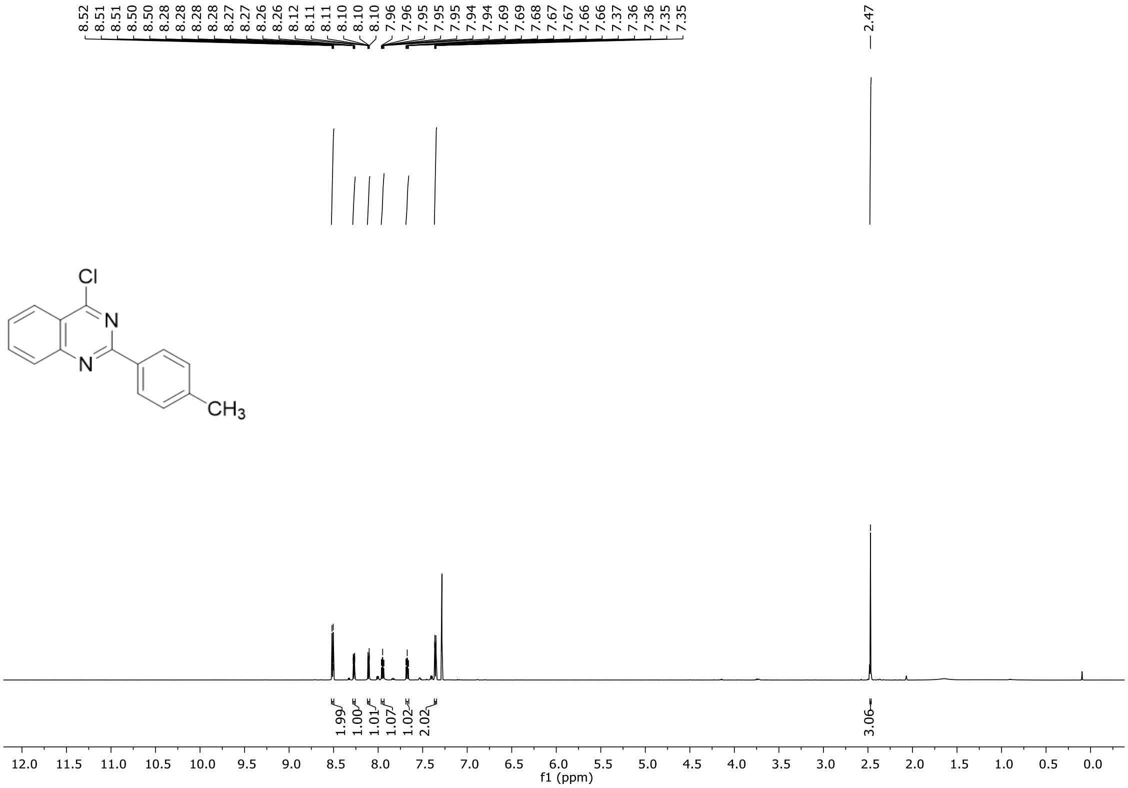

4,7-dichloroquinazoline (**5g**)

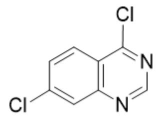

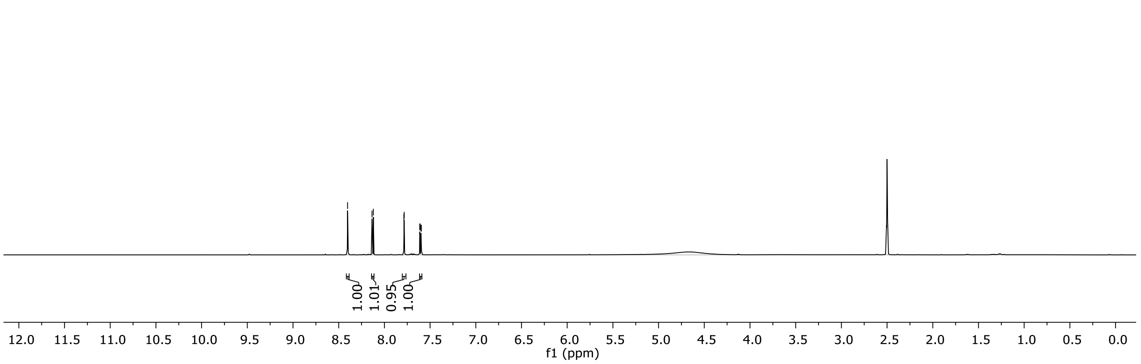

N-hexyl-2-phenylquinazolin-4-amine (**1.6**)

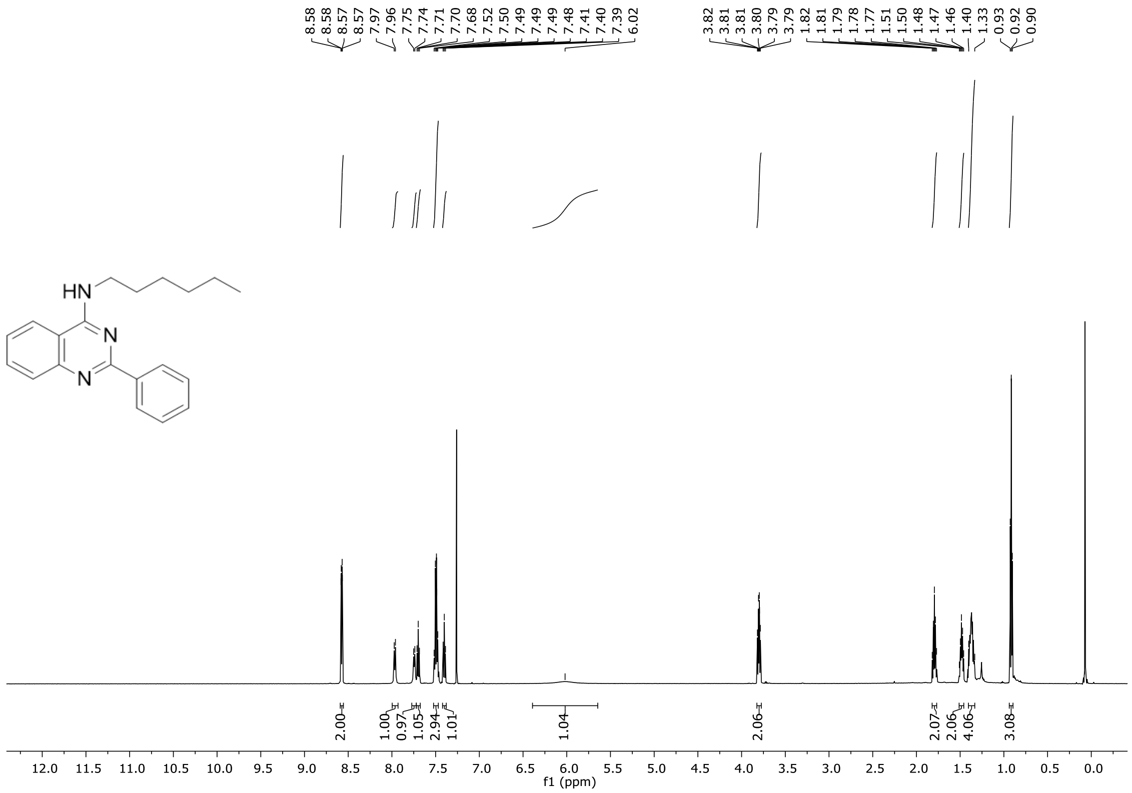

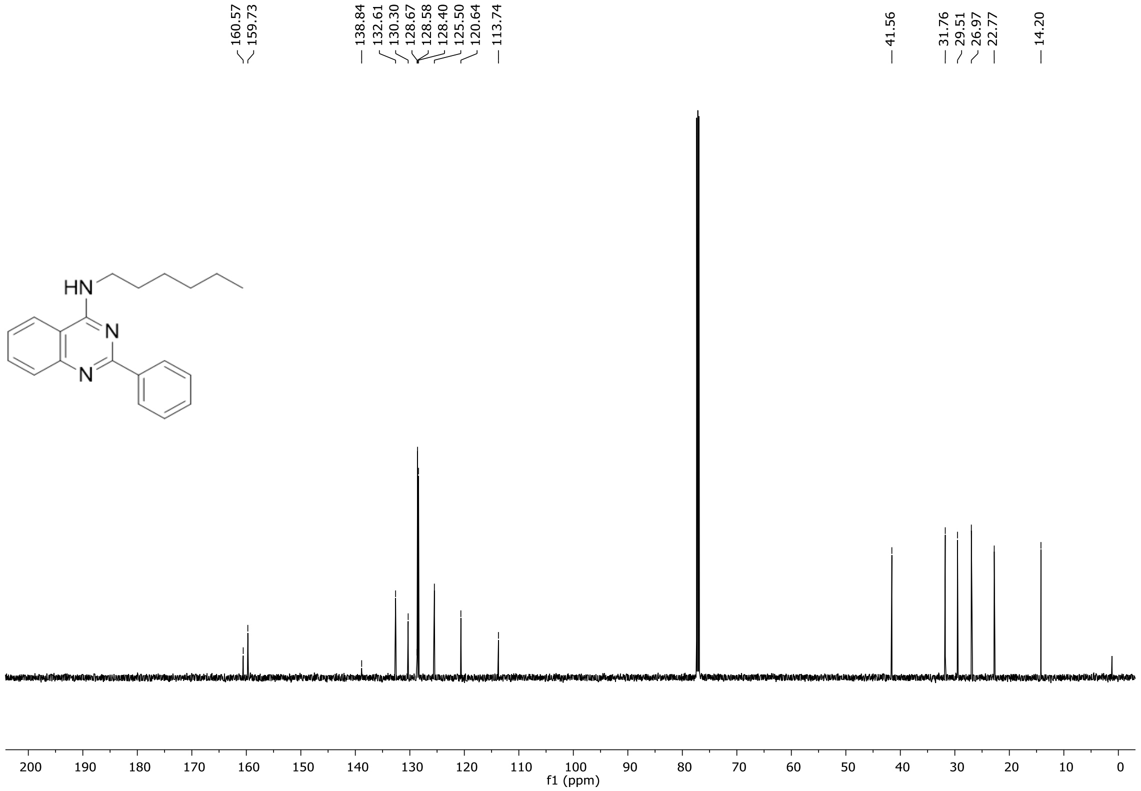

N[1],N[1]-dimethyl-N[3]-(2-phenylquinazolin-4-yl)propane-1,3-diamine (**1.7**)

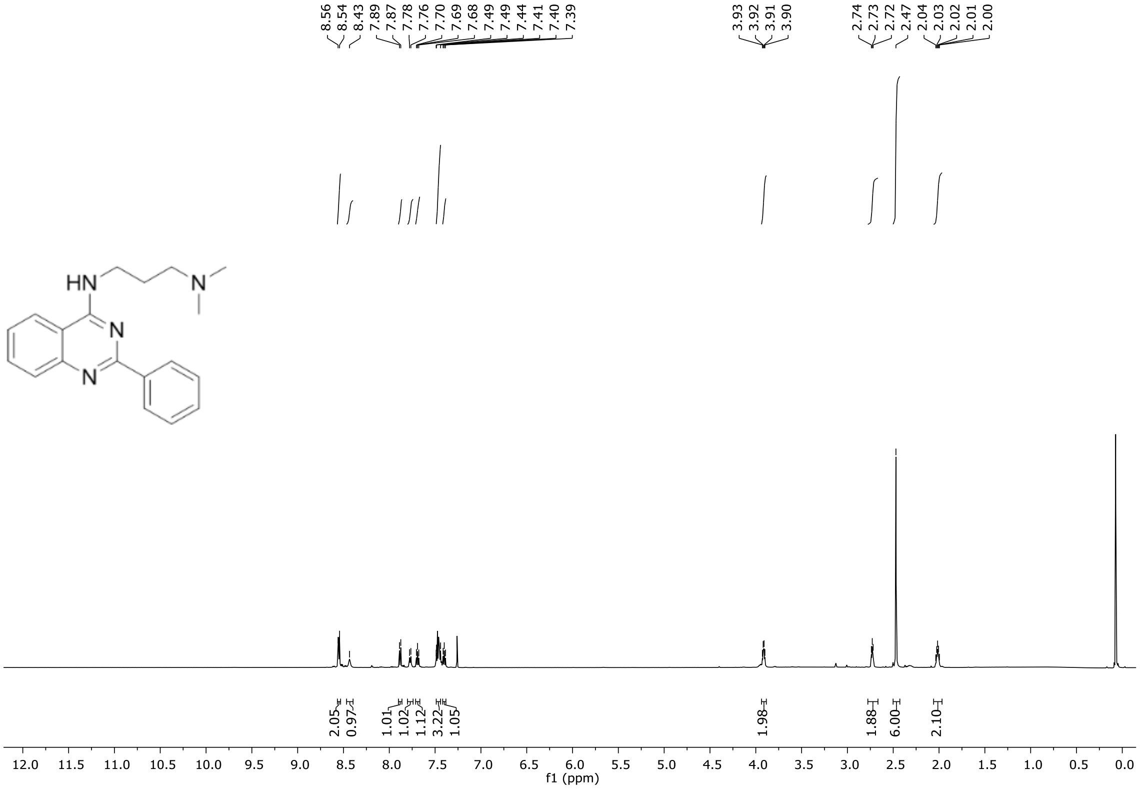

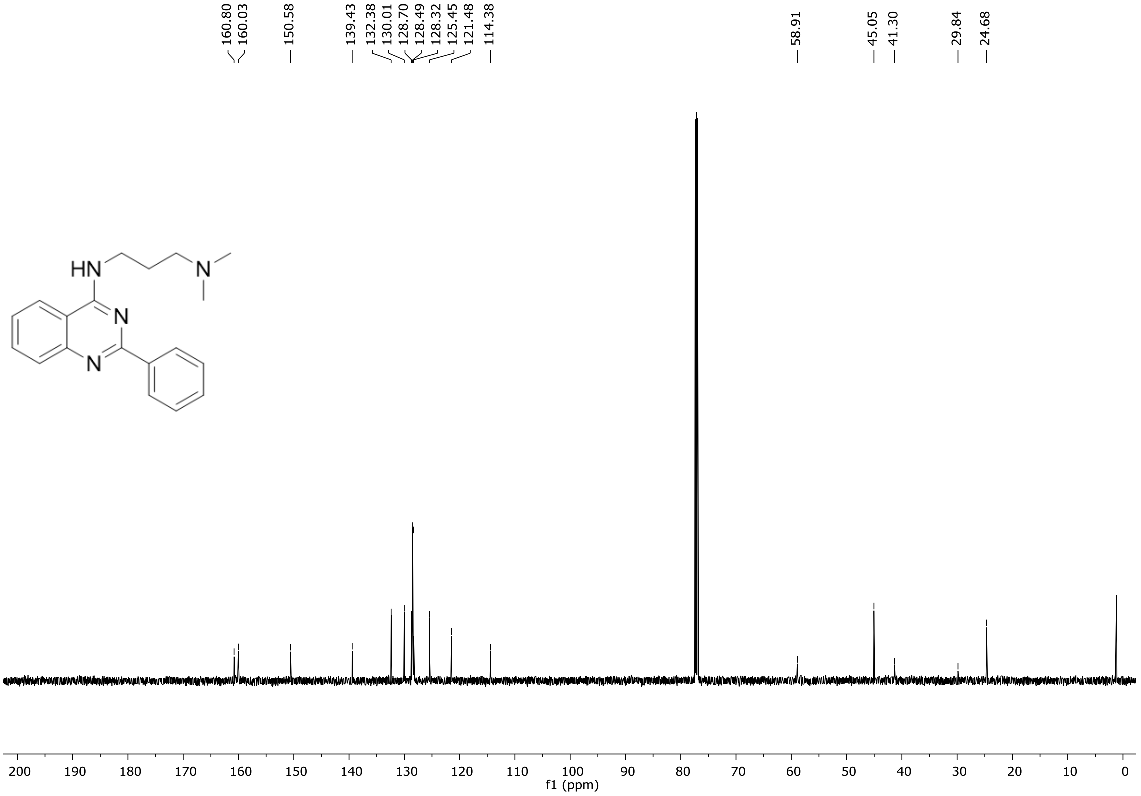

4-(4-methylpiperazin-1-yl)-2-phenylquinazoline (**1.8**)

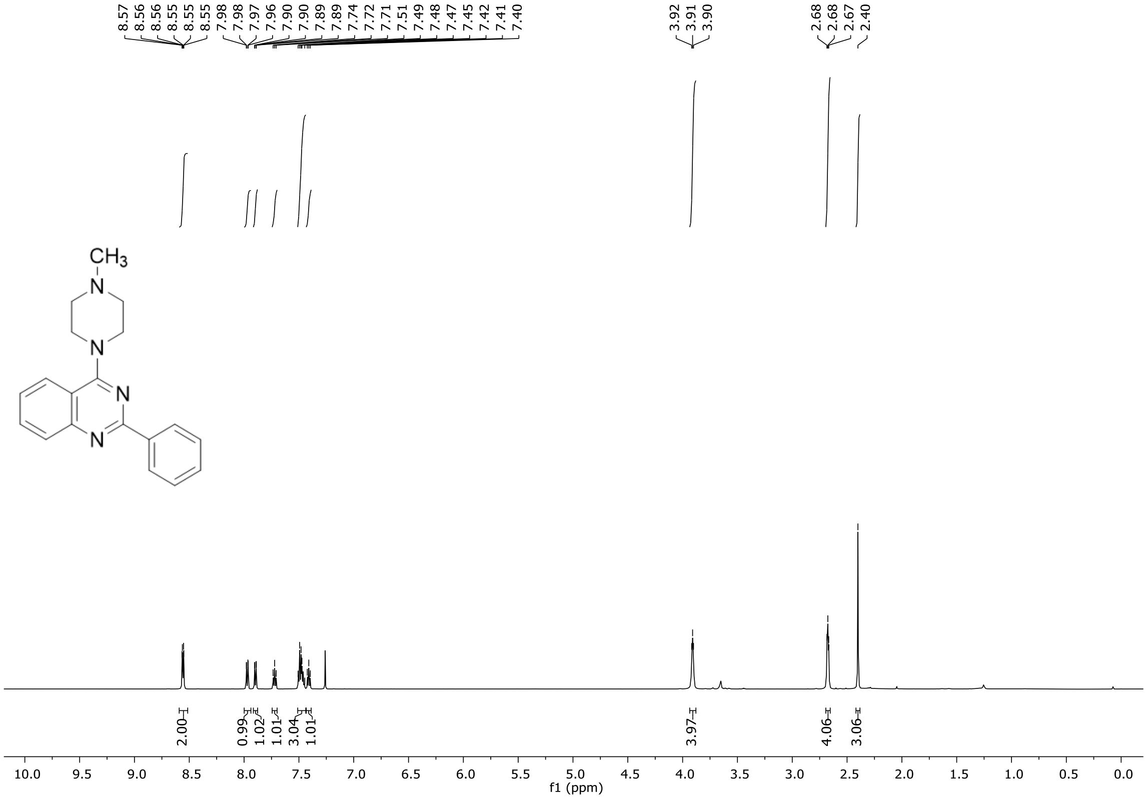

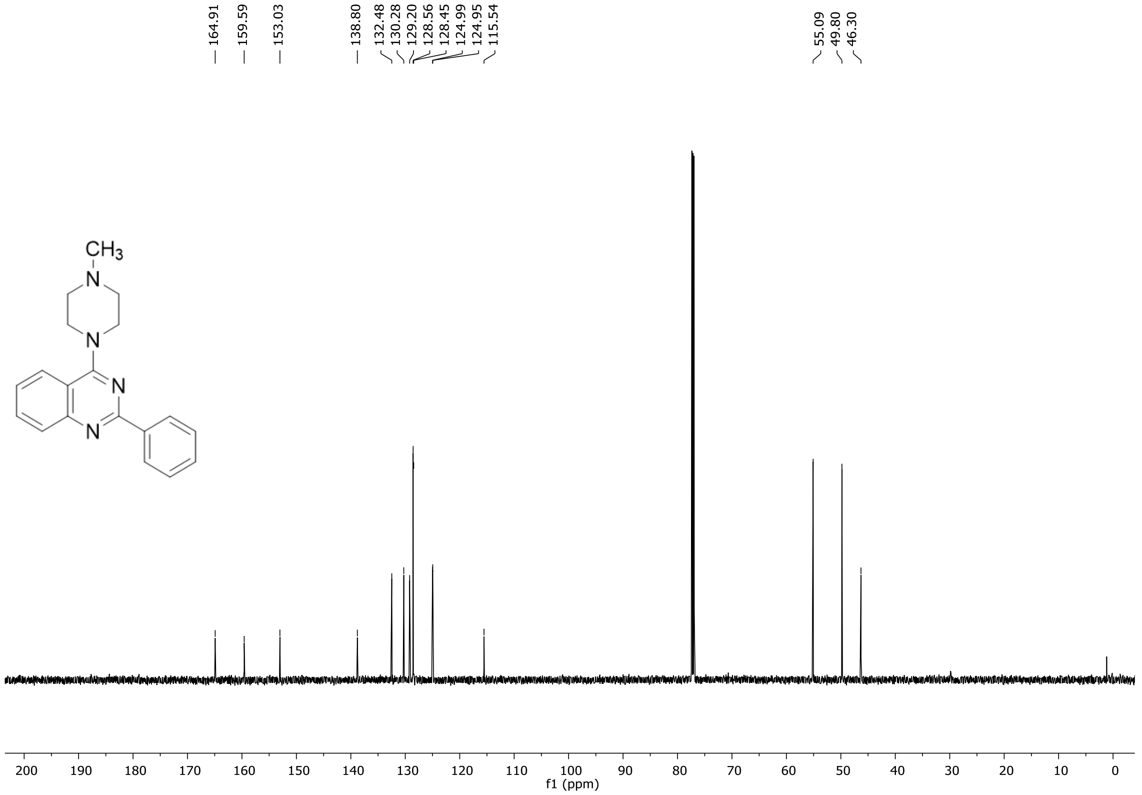

2-(4-(2-phenylquinazolin-4-yl)piperazin-1-yl)ethanol (**1.9**)

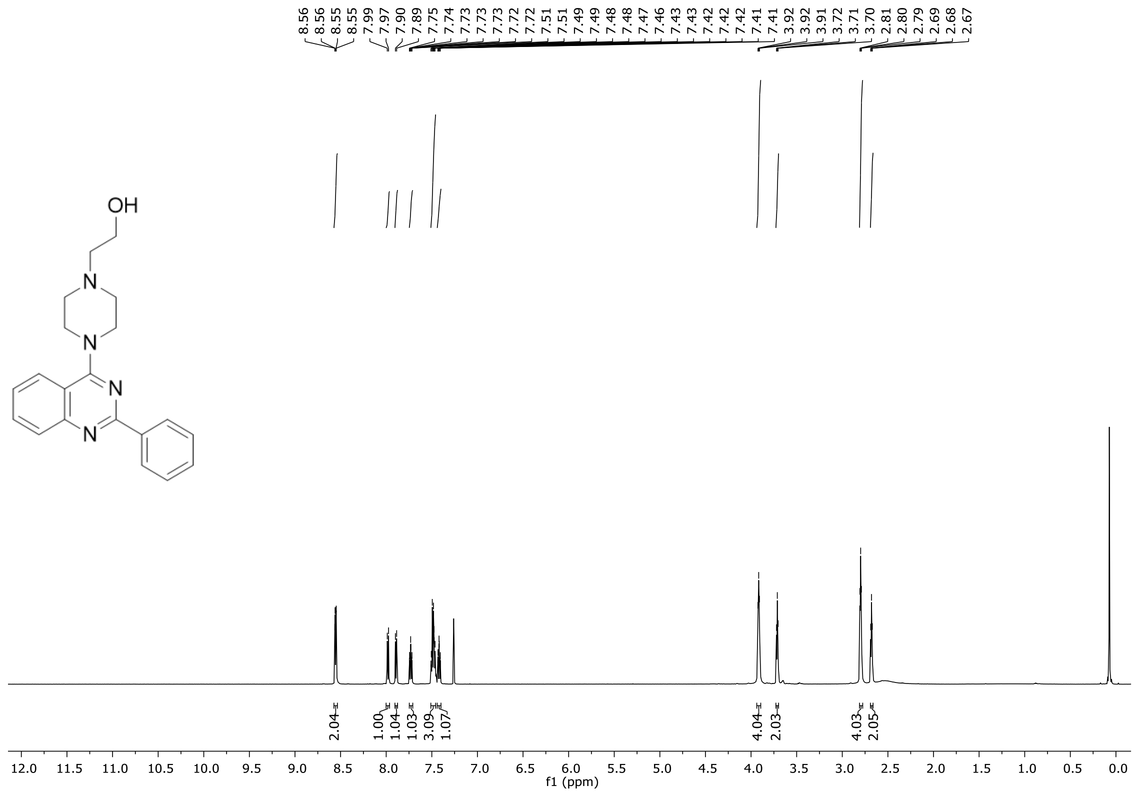

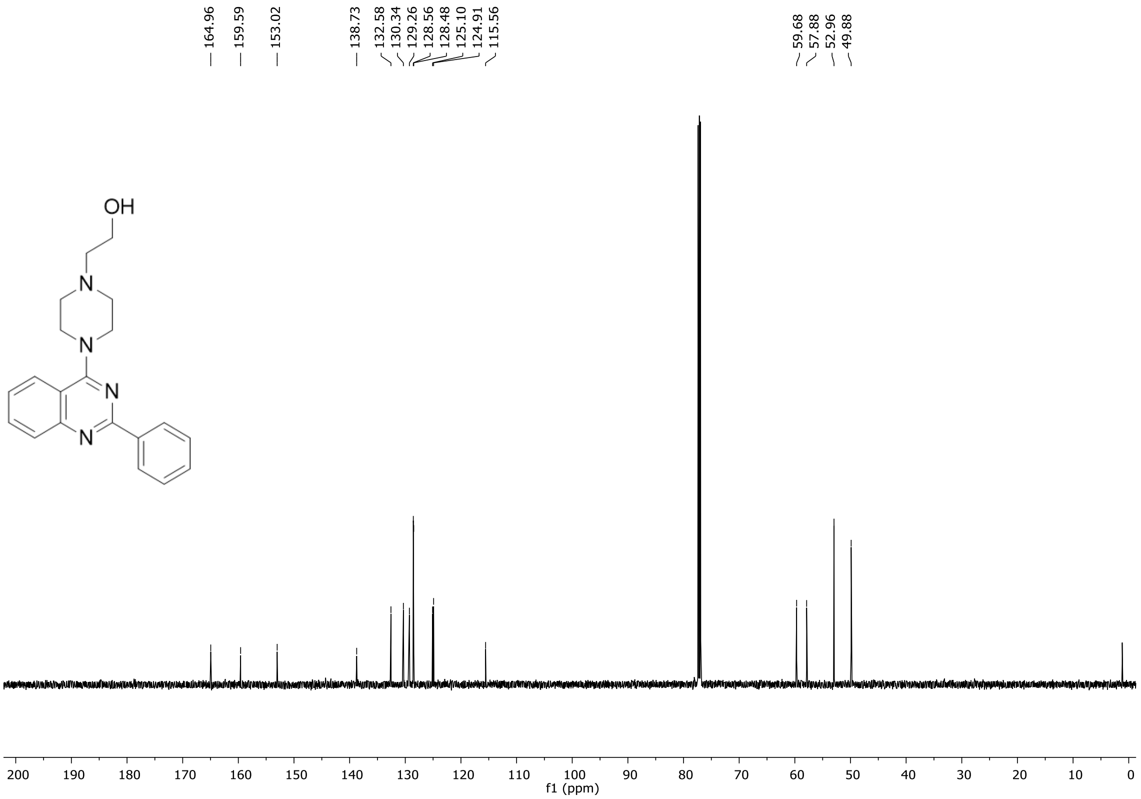

2-phenyl-N-(2-(piperidin-1-yl)ethyl)quinazolin-4-amine (**1.10**)

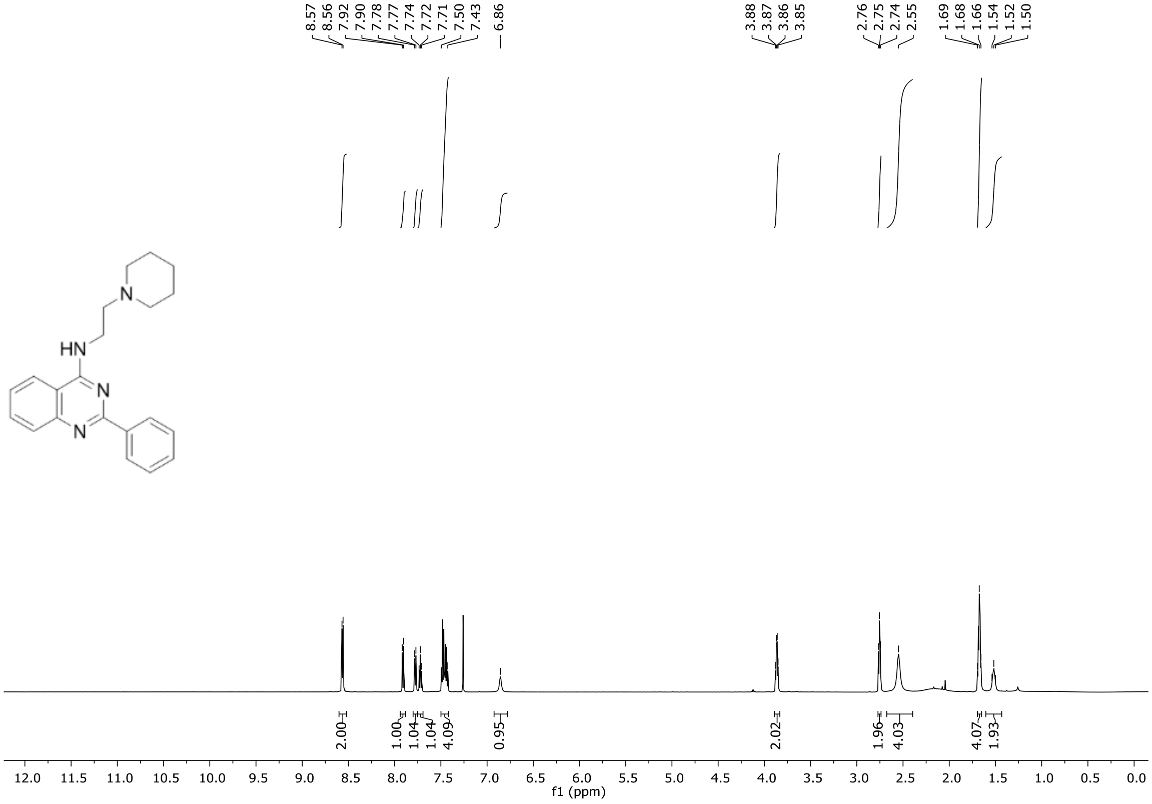

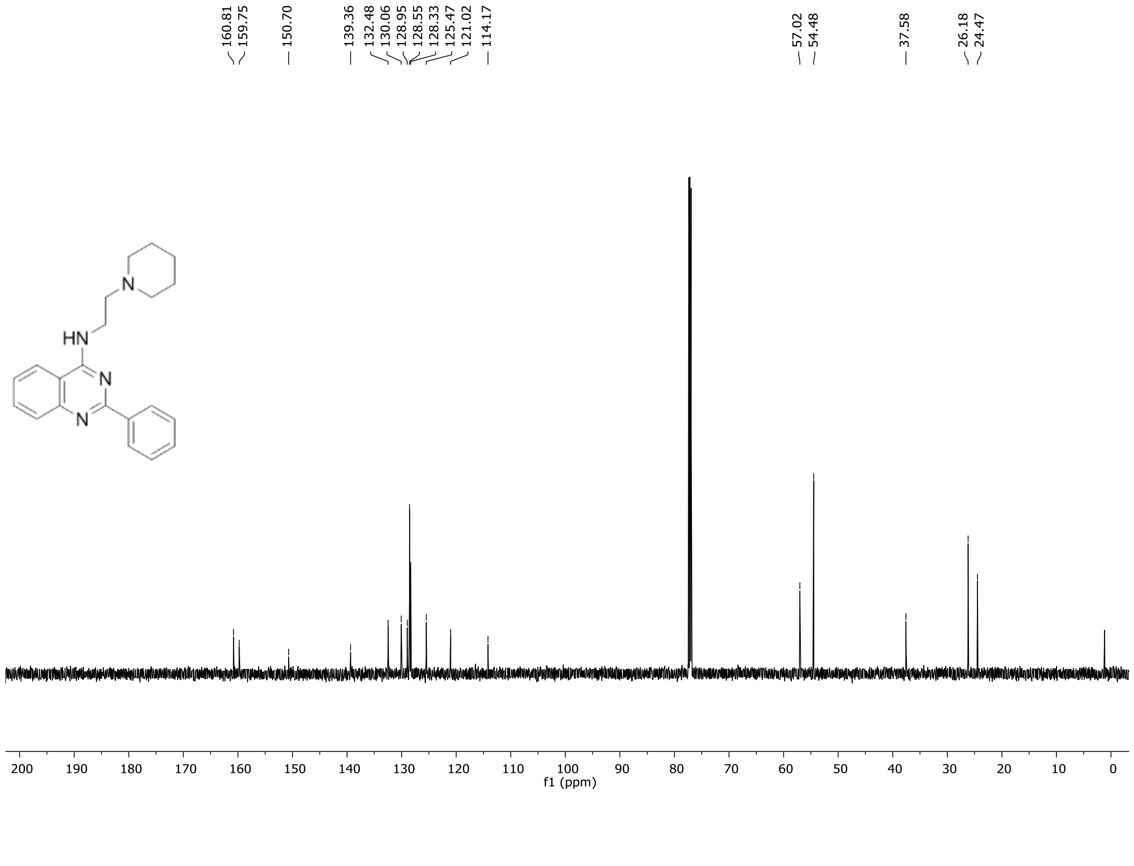

N-(1-benzylpiperidin-4-yl)-2-phenylquinazolin-4-amine (**1.11**)

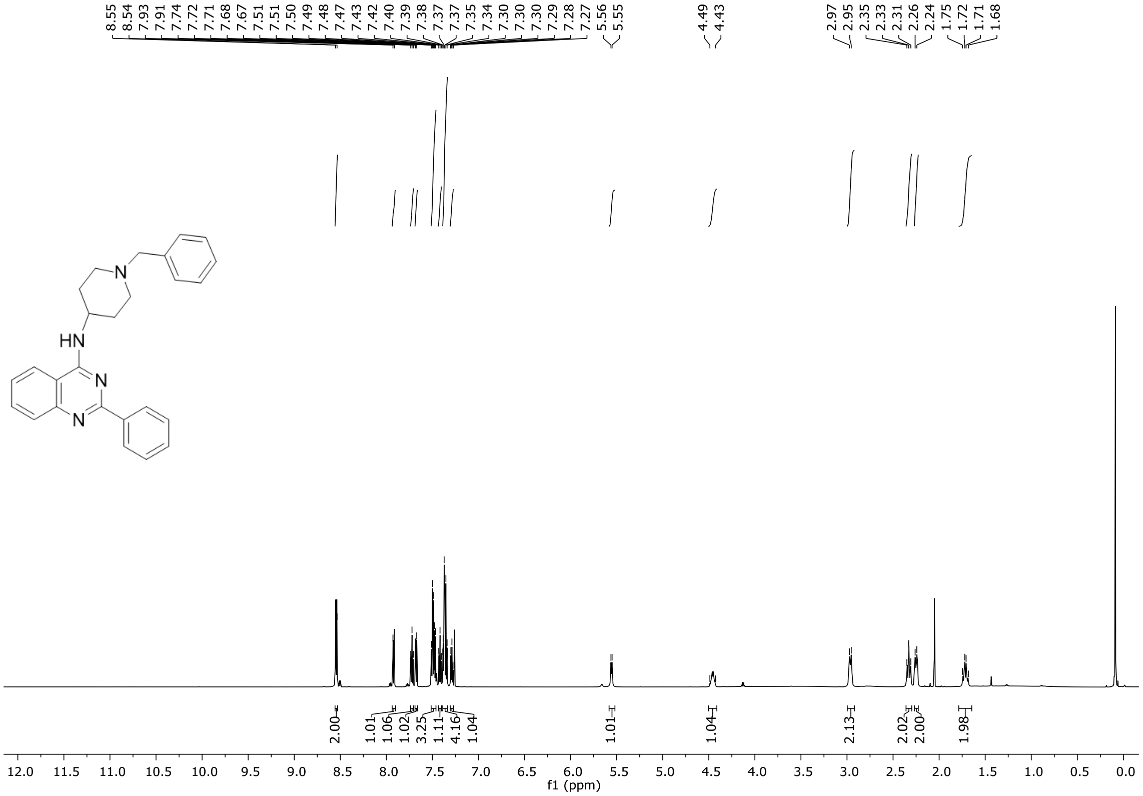

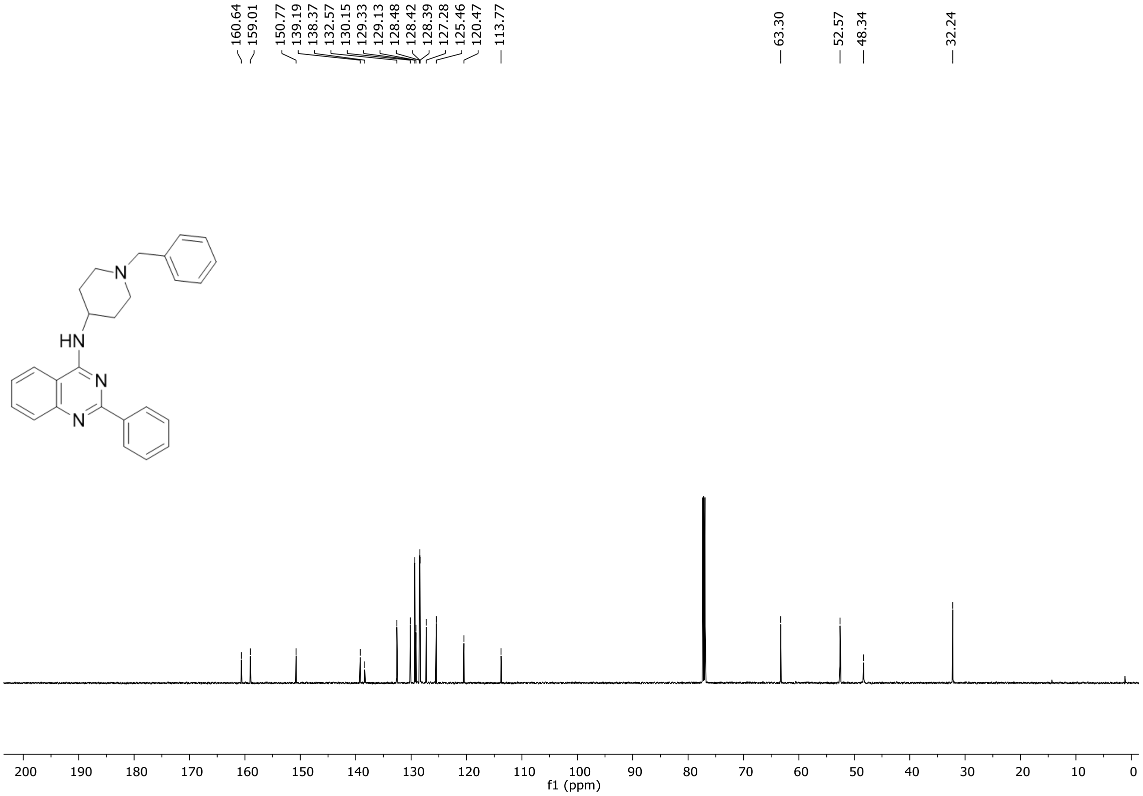

2-phenyl-N-(2,2,6,6-tetramethylpiperidin-4-yl)quinazolin-4-amine (**1.12**)

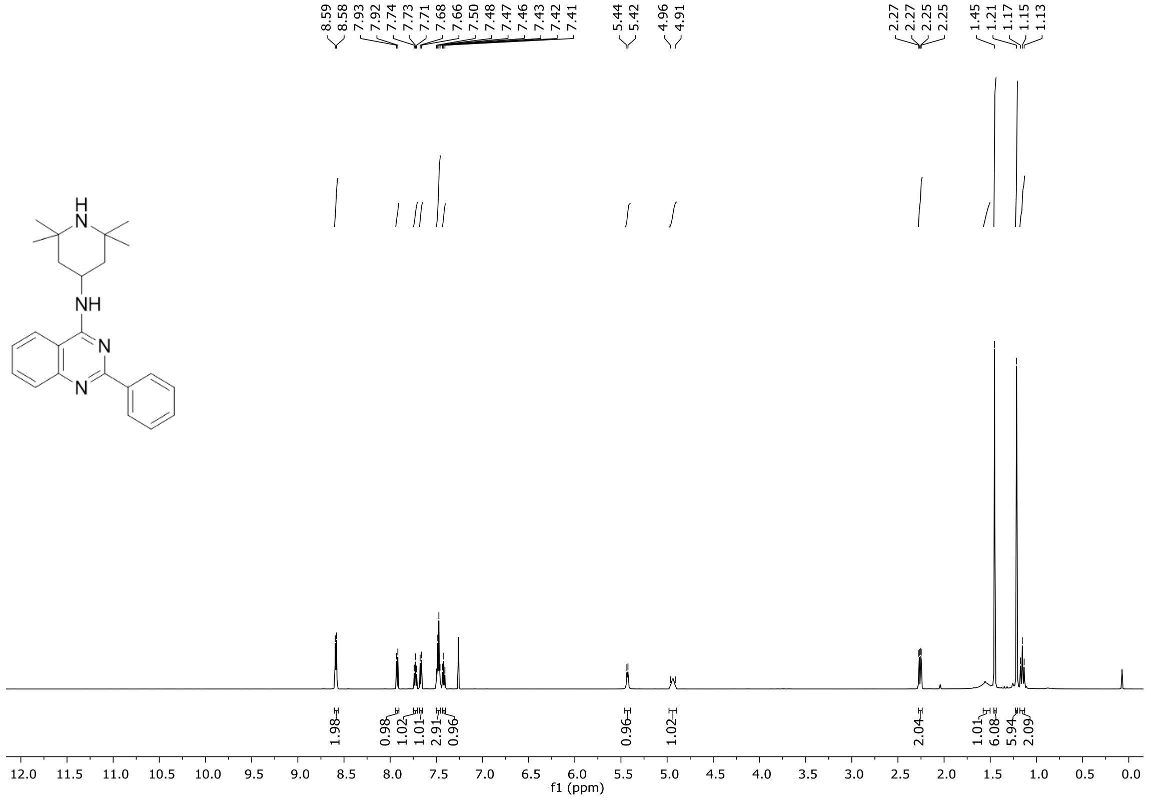

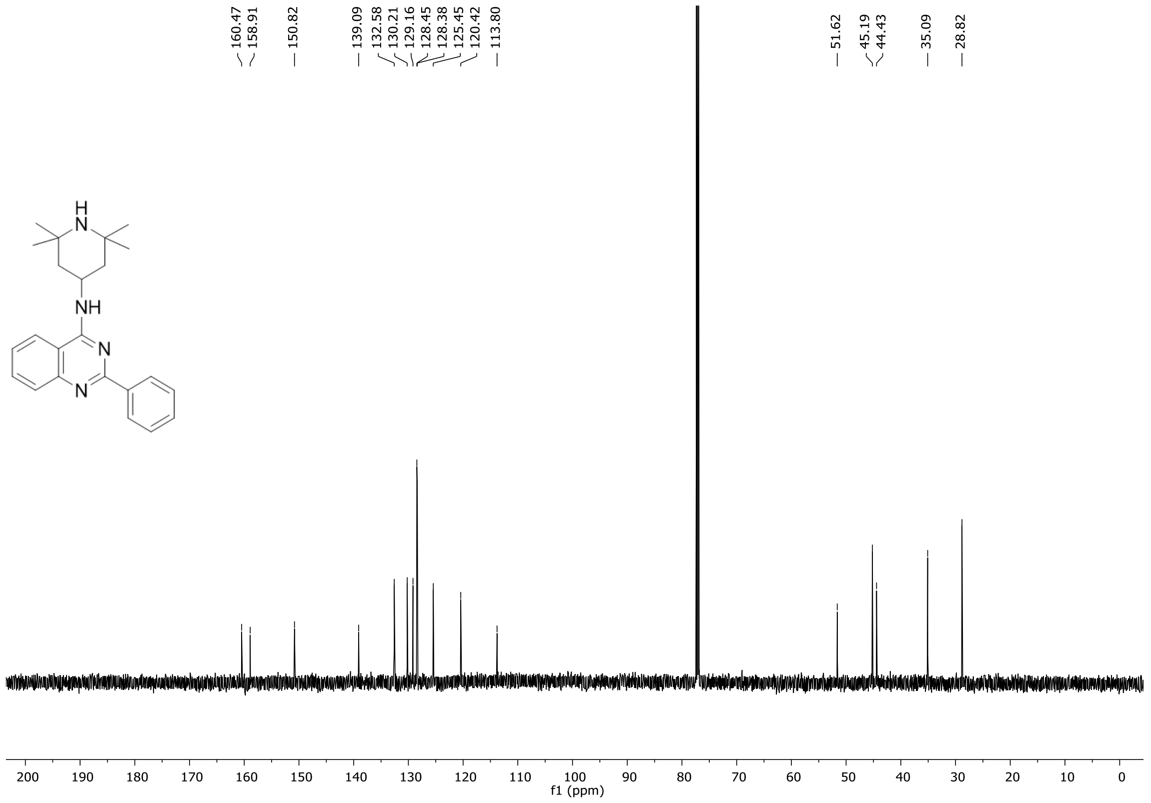

4-(((2-phenylquinazolin-4-yl)amino)methyl)pyridine 1-oxide (**1.13**)

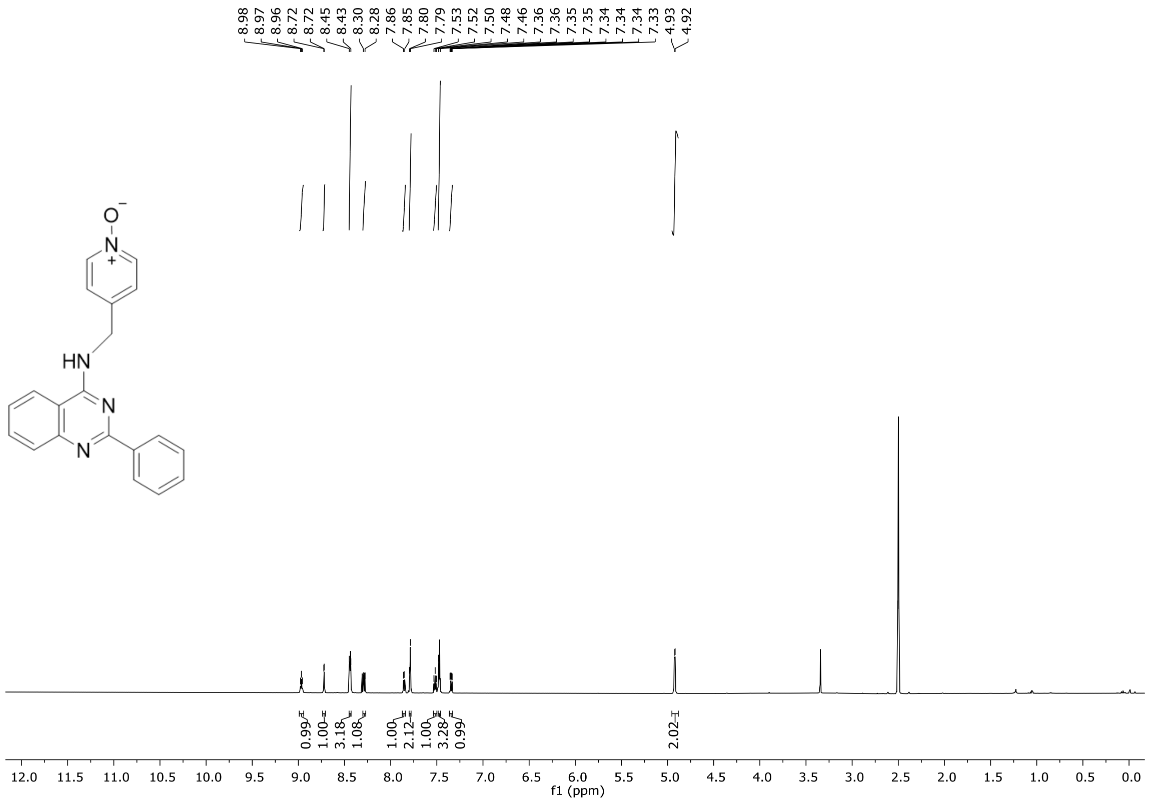

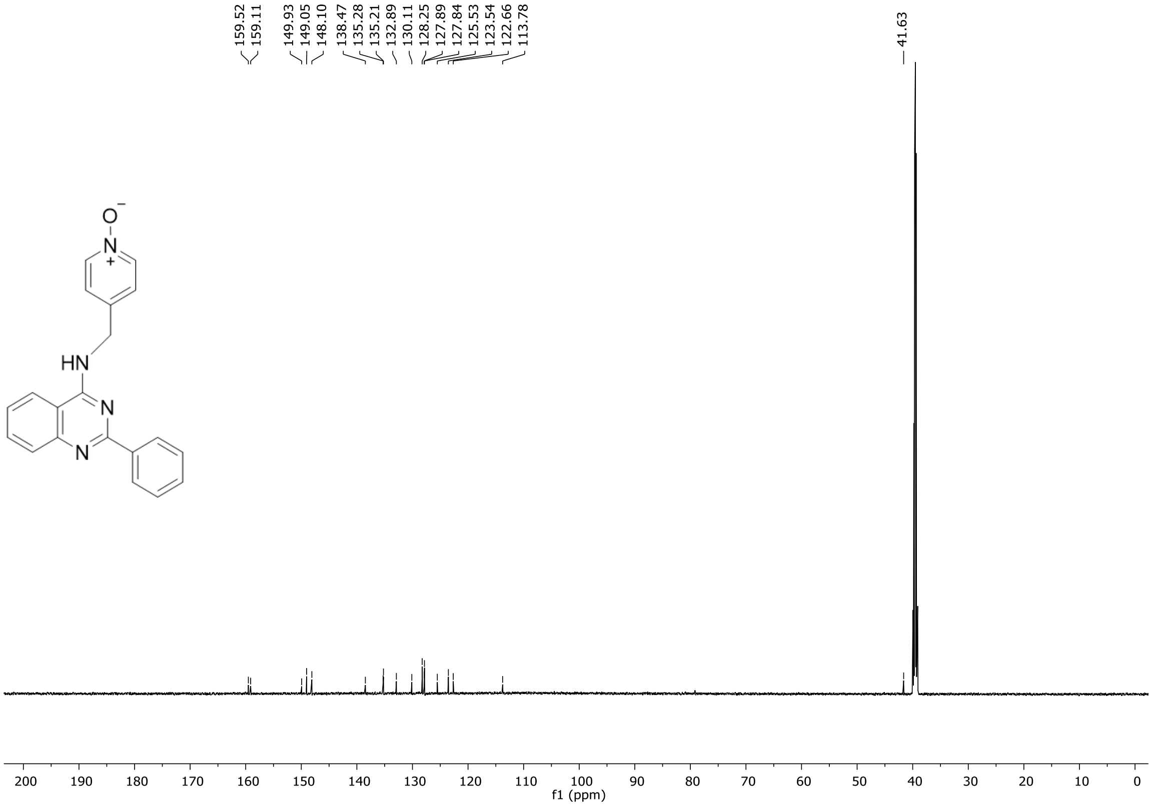

4-(4-methylpiperazin-1-yl)-2-(4-nitrophenyl)quinazoline (**1.14**)

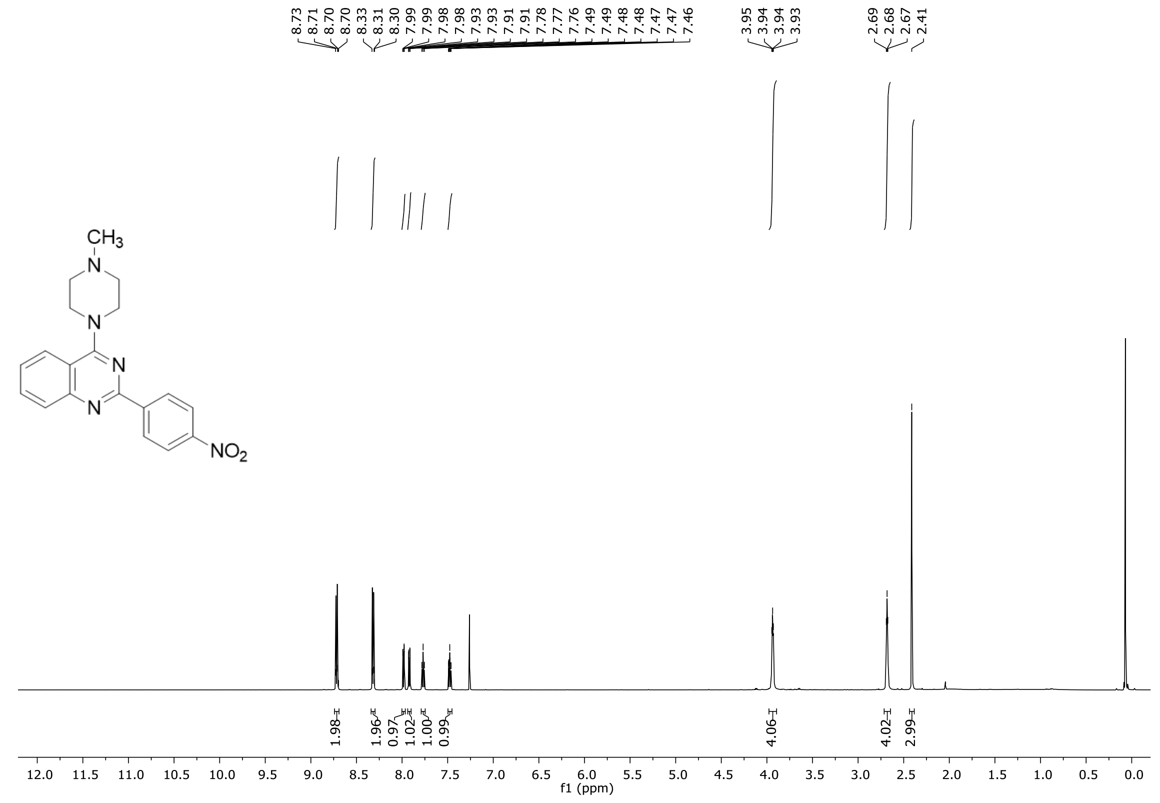

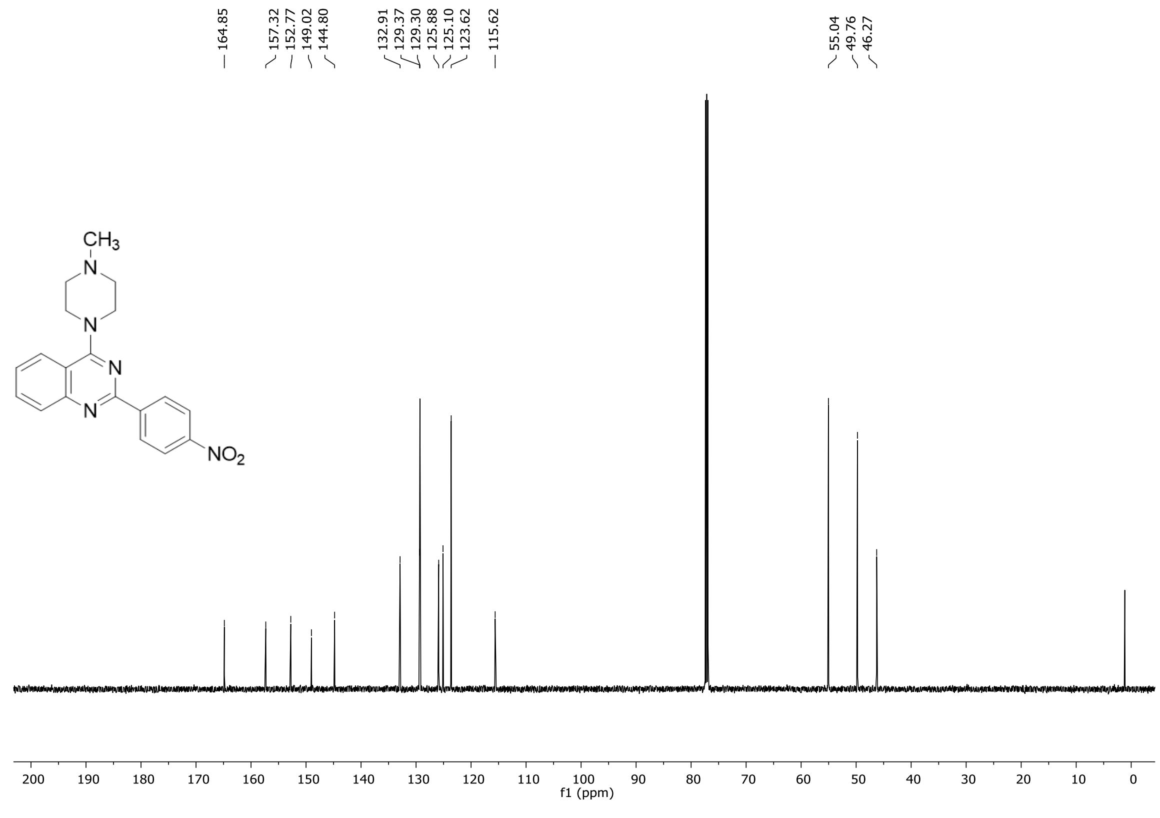

2-(4-nitrophenyl)-N-(2-(piperidin-1-yl)ethyl)quinazolin-4-amine (**1.15**)

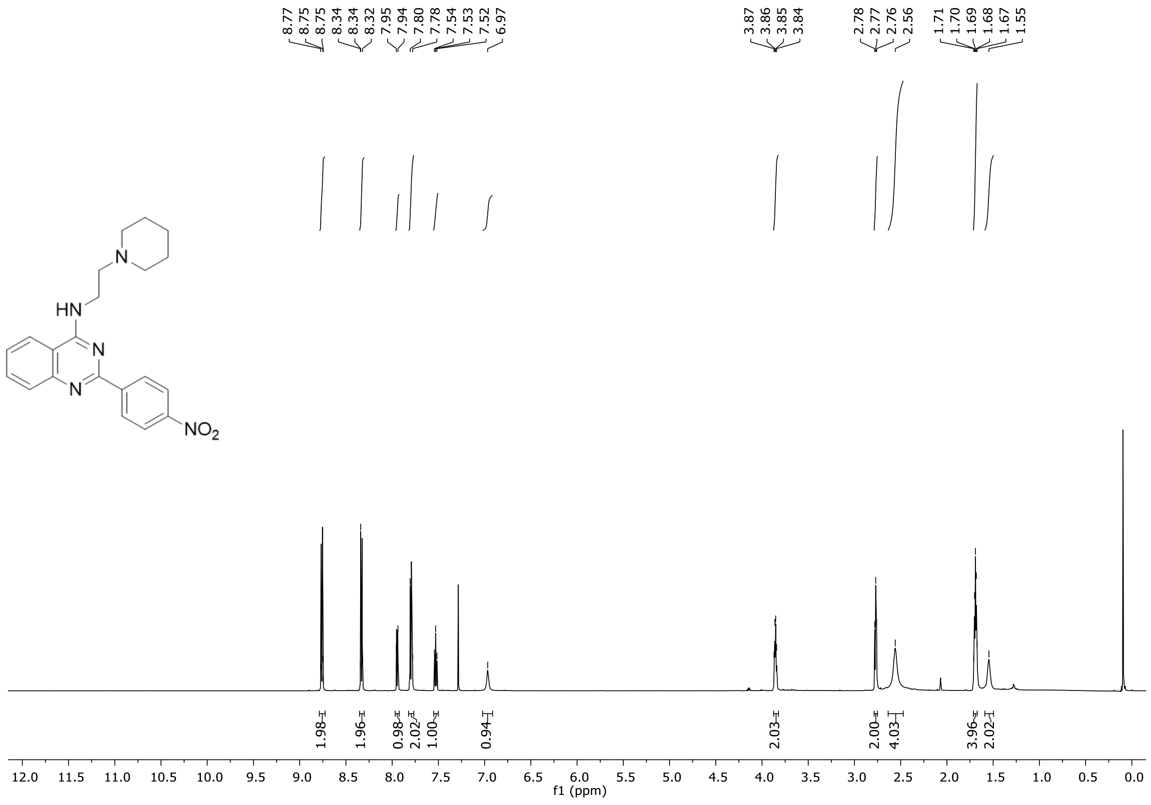

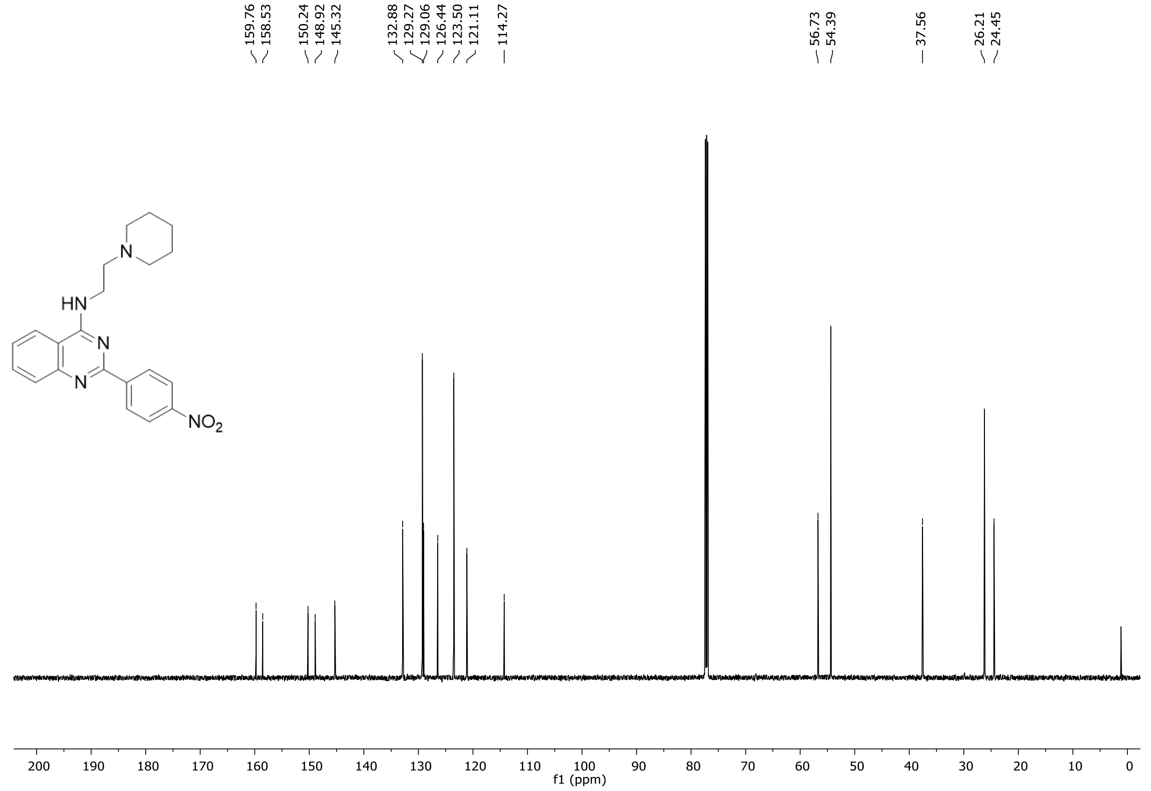

2-(4-(2-(4-nitrophenyl)quinazolin-4-yl)piperazin-1-yl)etanol (**1.16**)

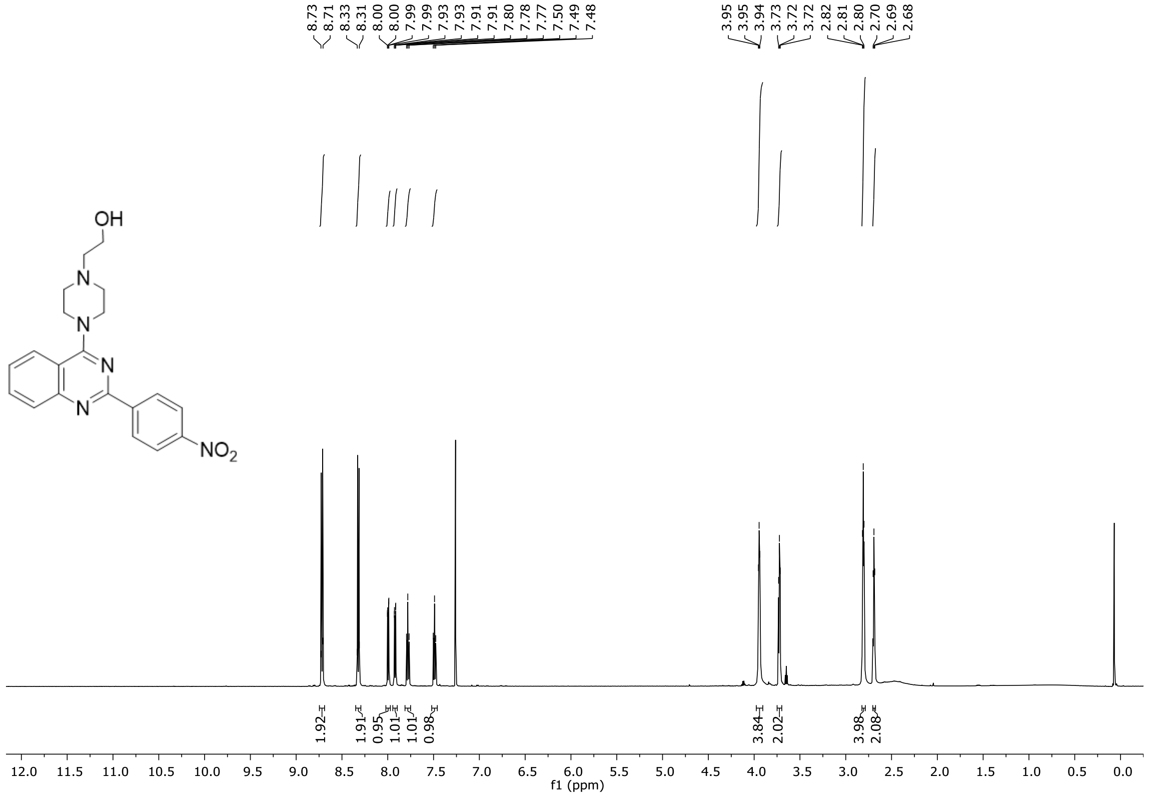

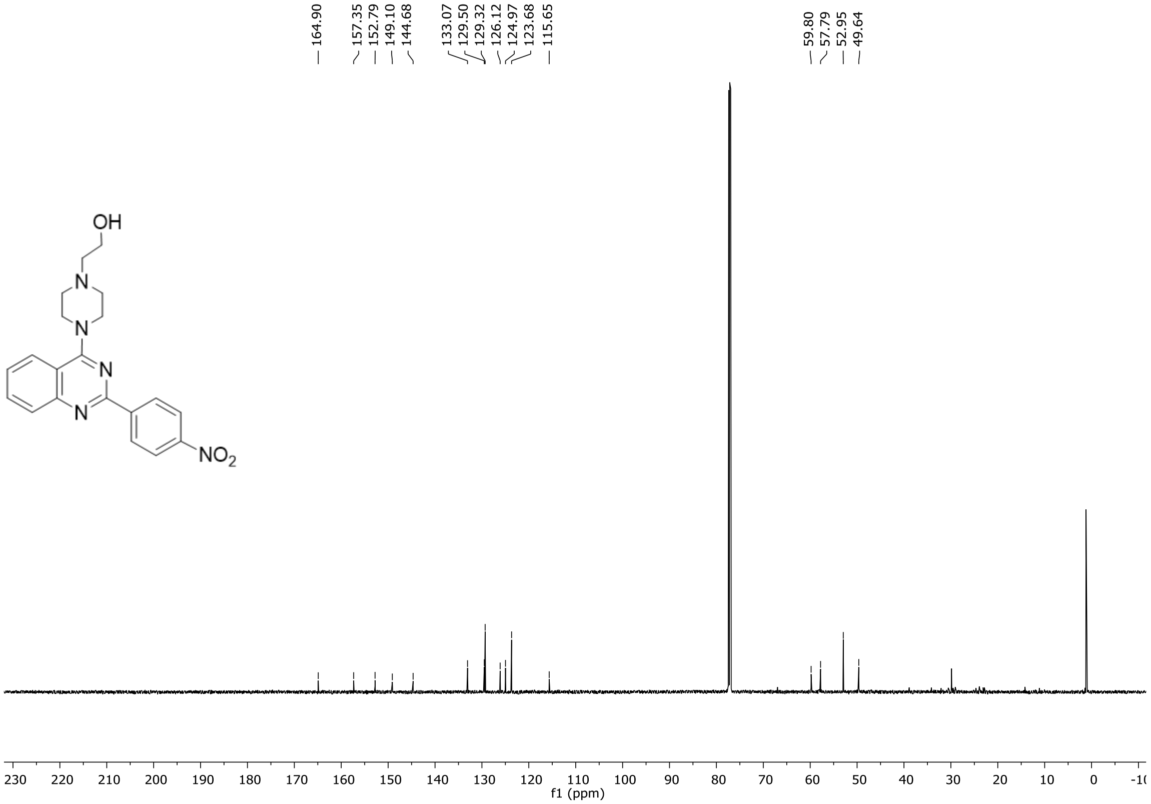

2-(4-(2-(2,4-dichlorophenyl)quinazolin-4-yl)piperazin-1-yl)etanol (**1.17**)

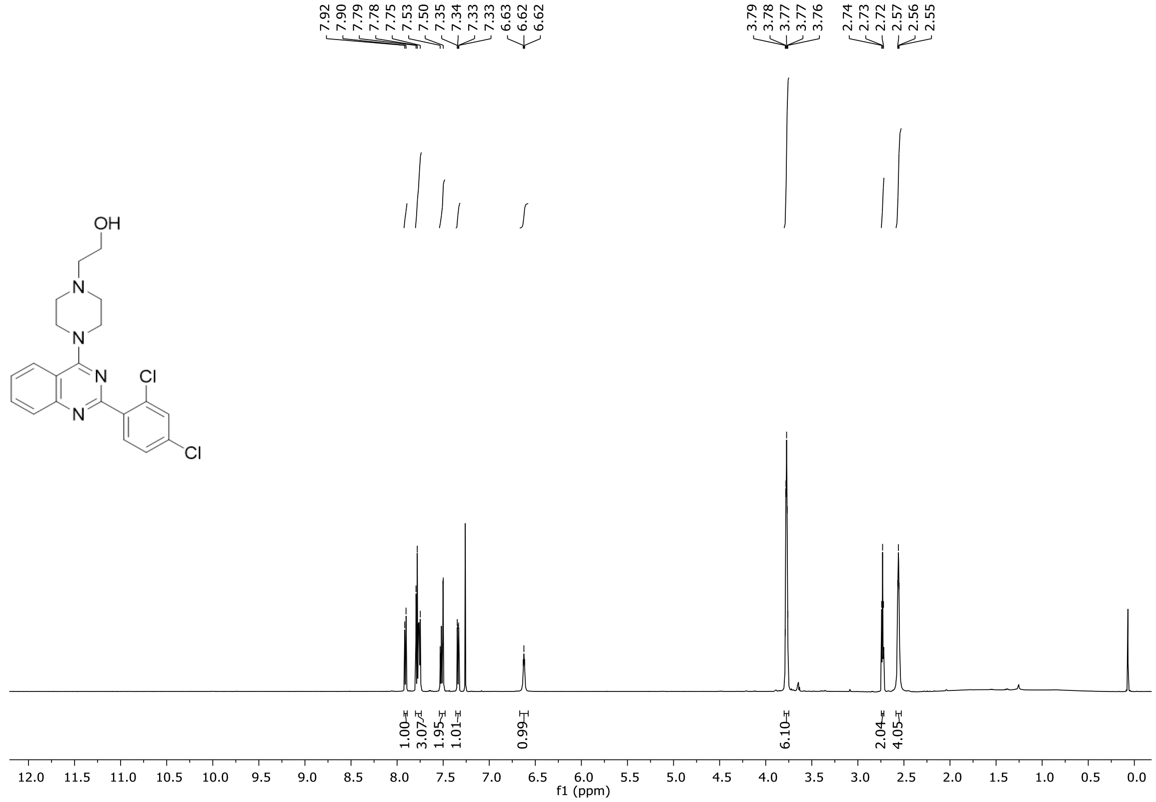

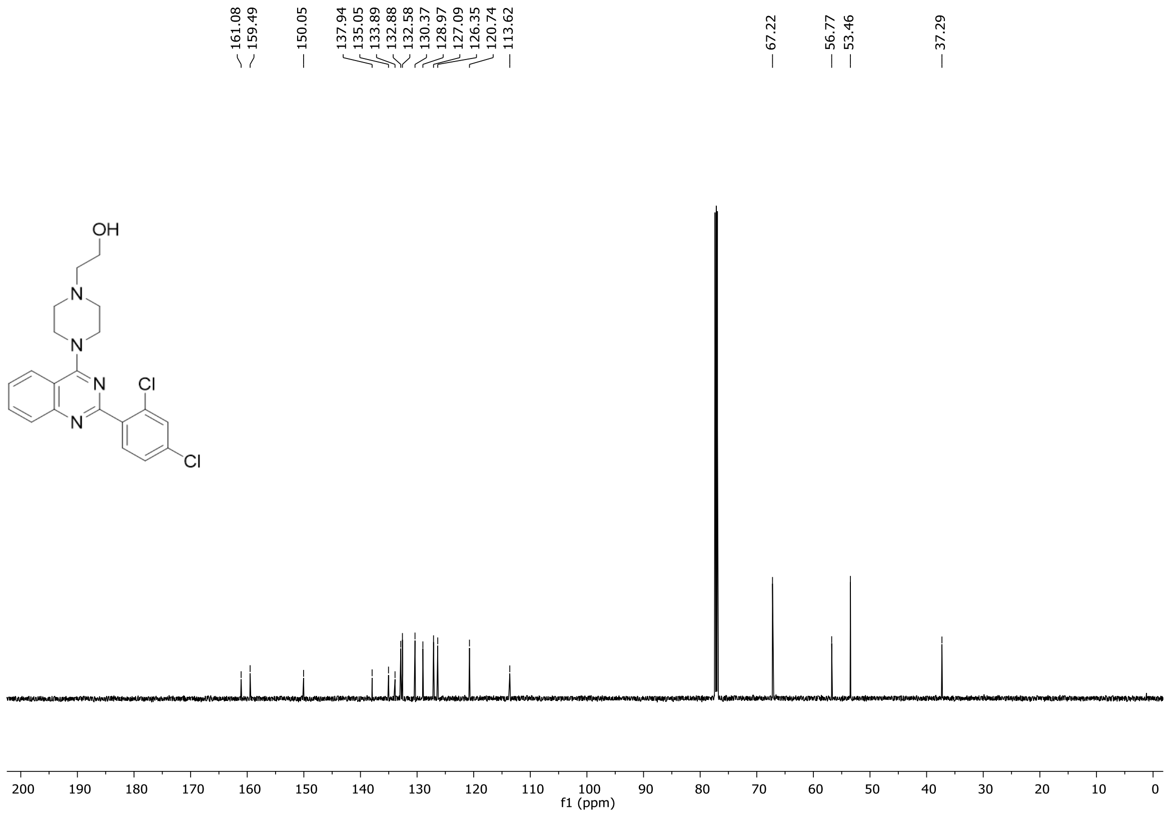

2-(4-(2-[(4-dimethylamino)phenyl]quinazolin-4-yl)piperazin-1-yl)ethanol (**1.18**)

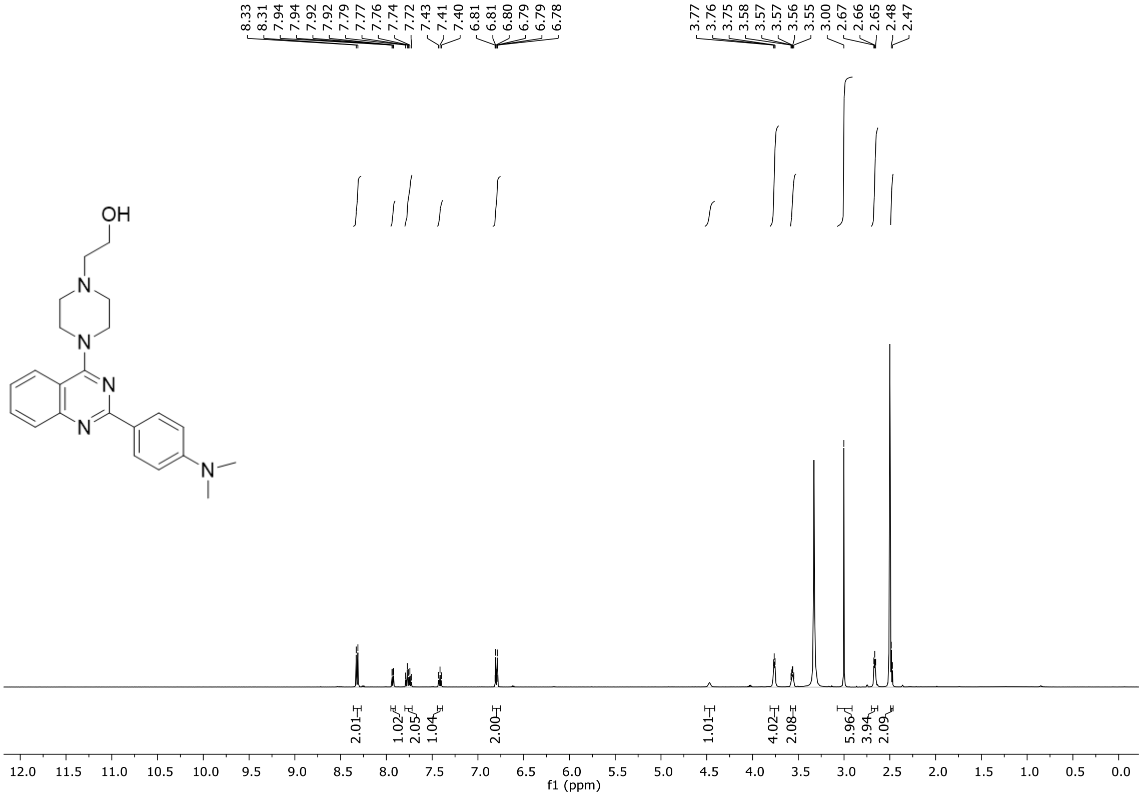

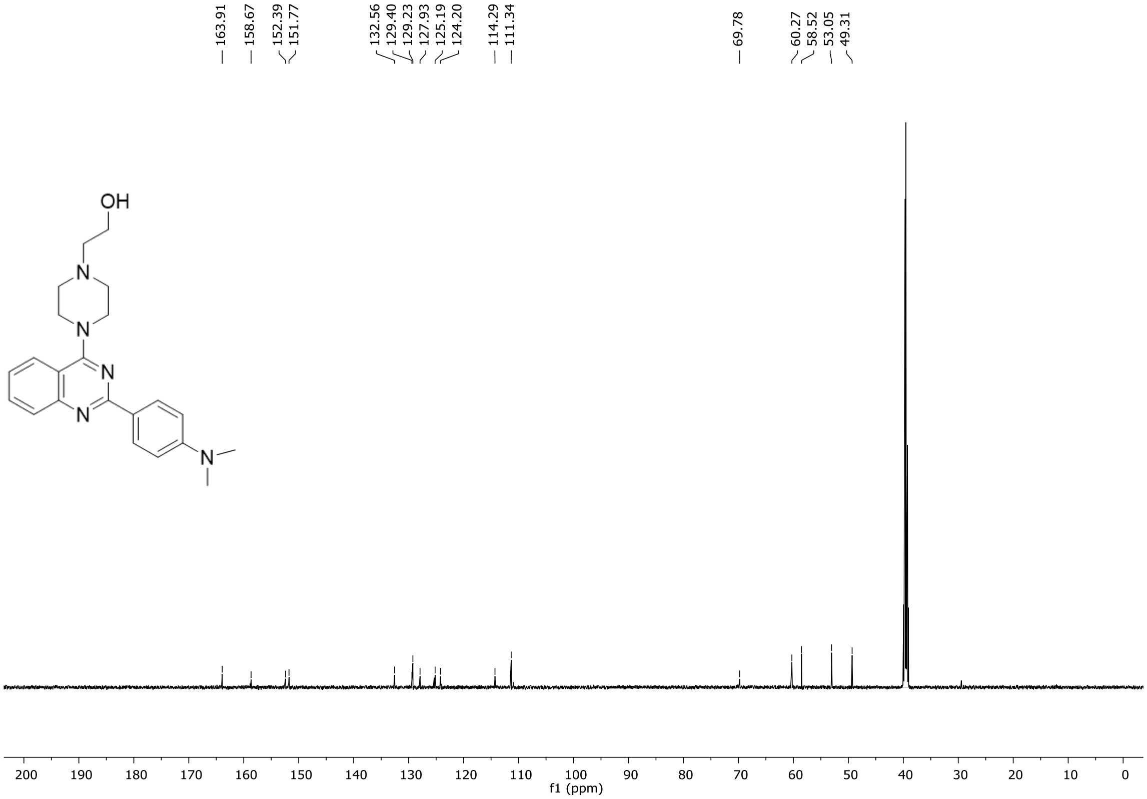

2-(4-methoxyphenyl)-4-(4-methylpiperazin-1-yl)quinazoline (**1.19**)

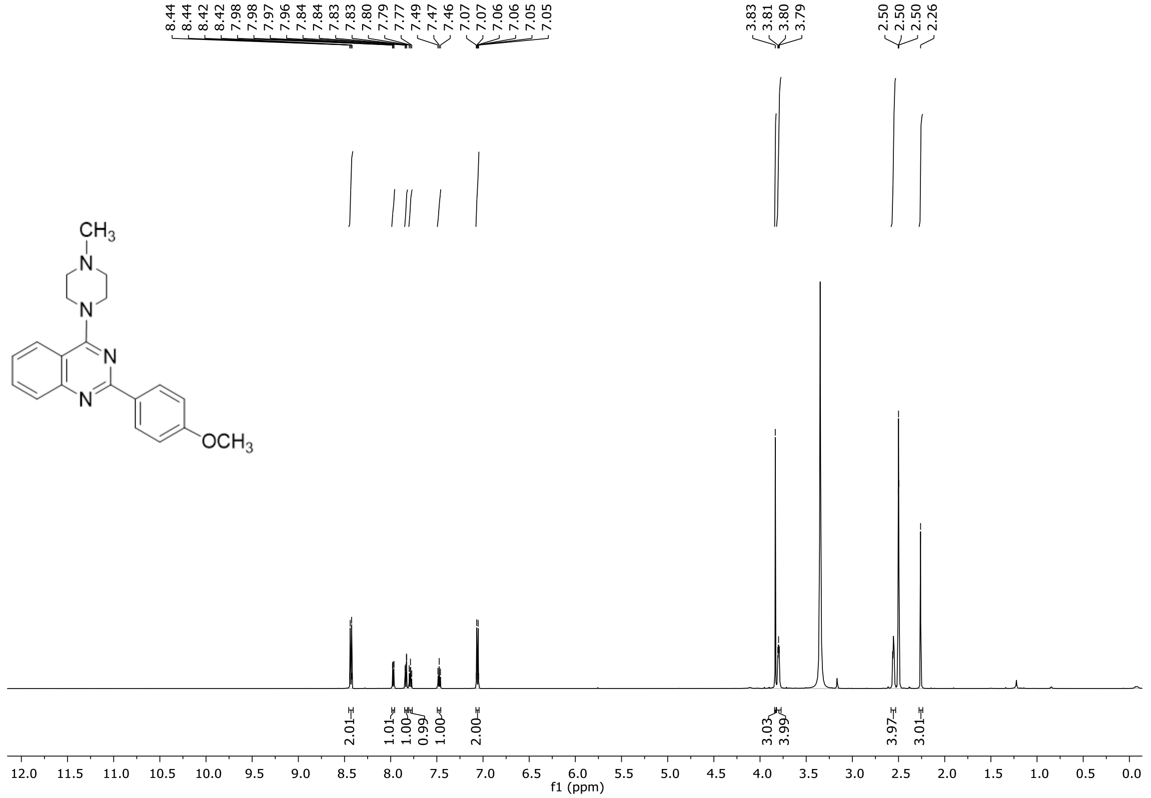

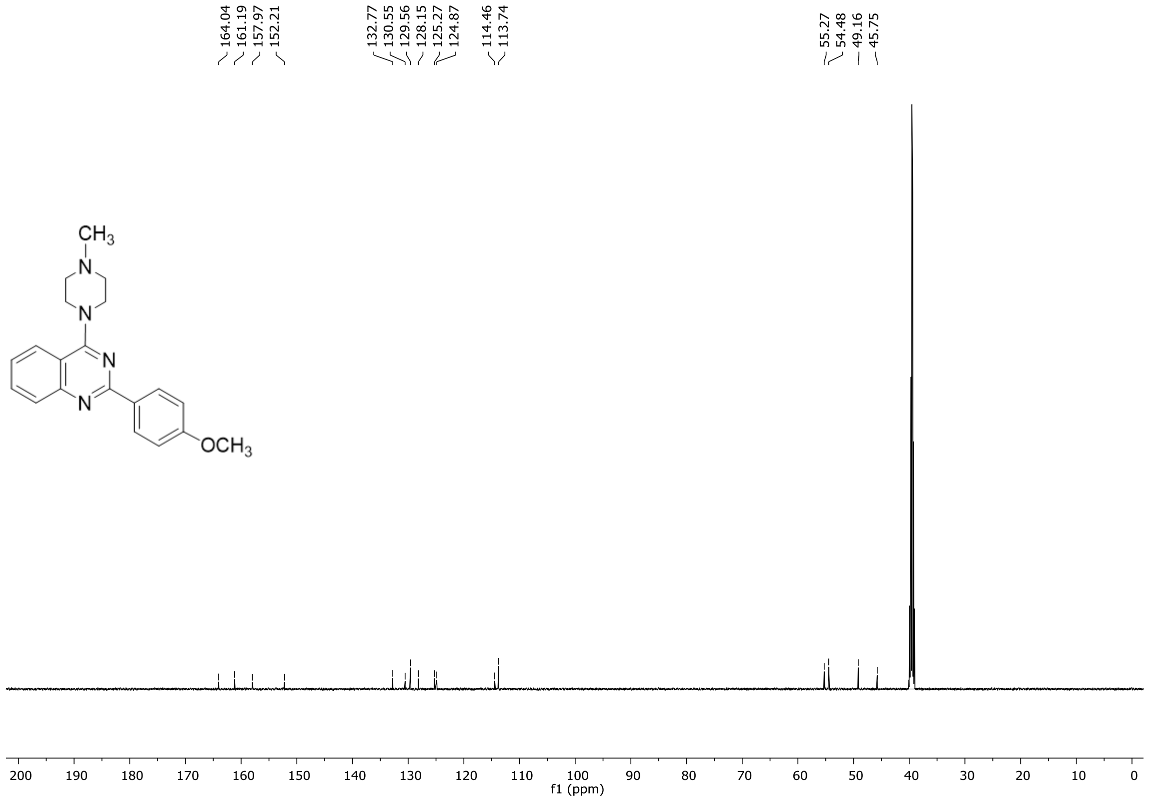

2-(4-(2-(p-tolyl)quinazolin-4-yl)piperazin-1-yl)etanol (**1.20**)

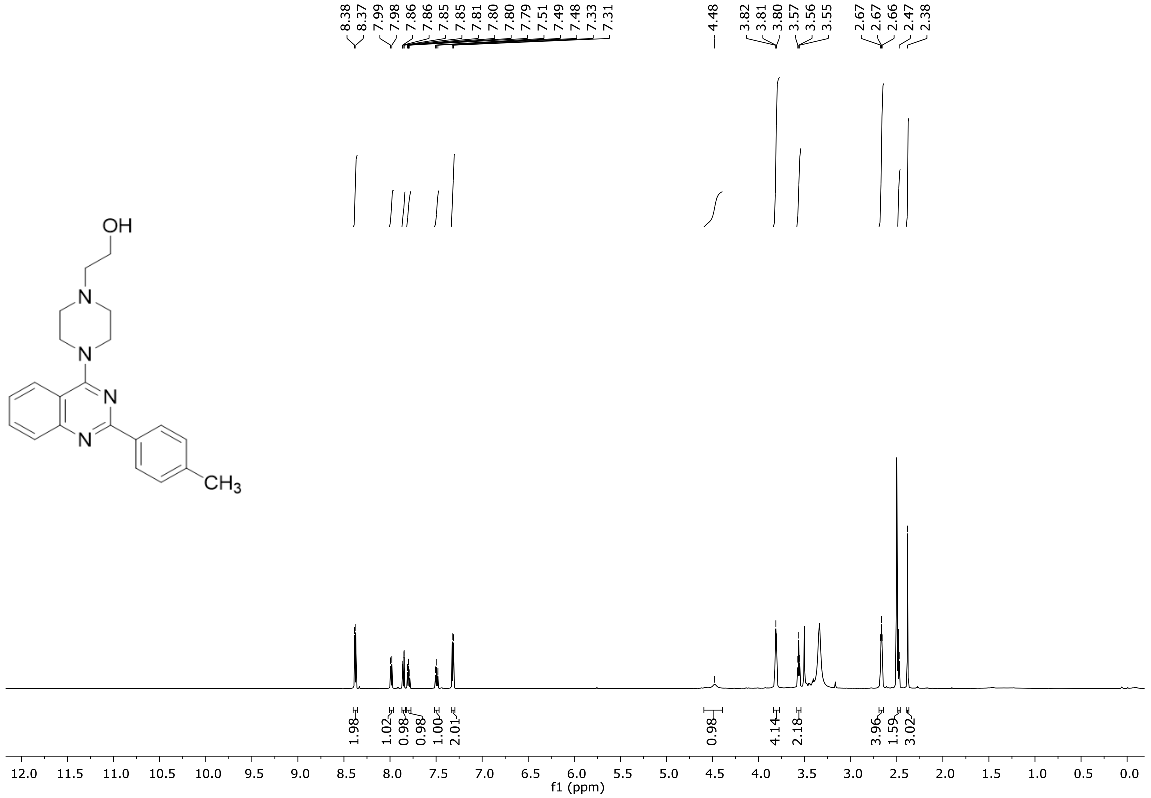

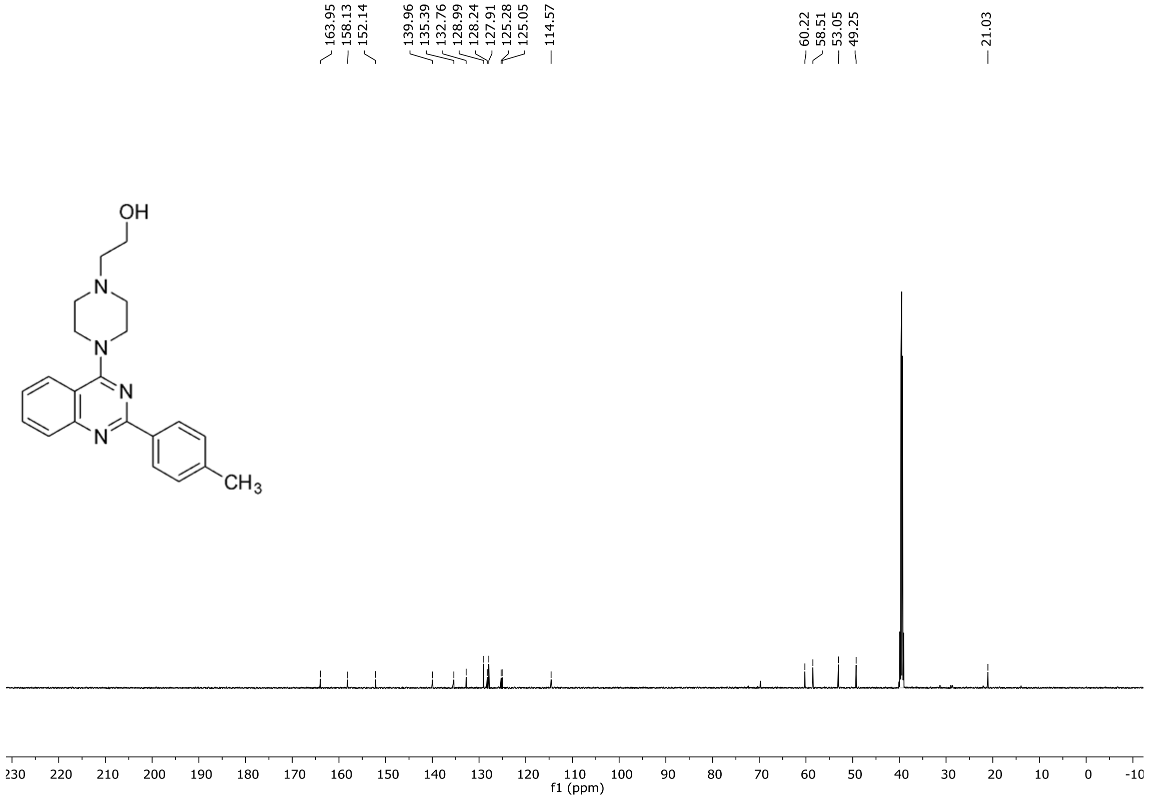

7-chloro- N-(2-morpholinoethyl)quinazolin-4-amine (**1.21**)

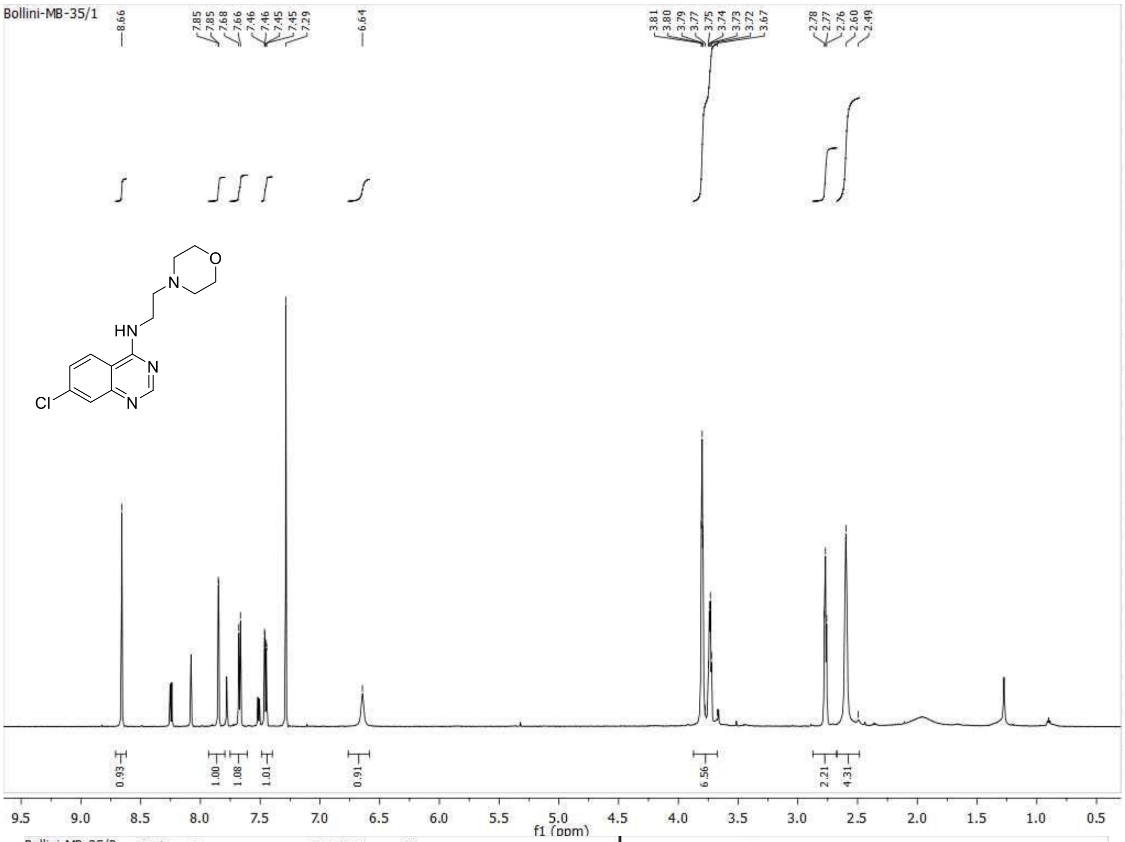

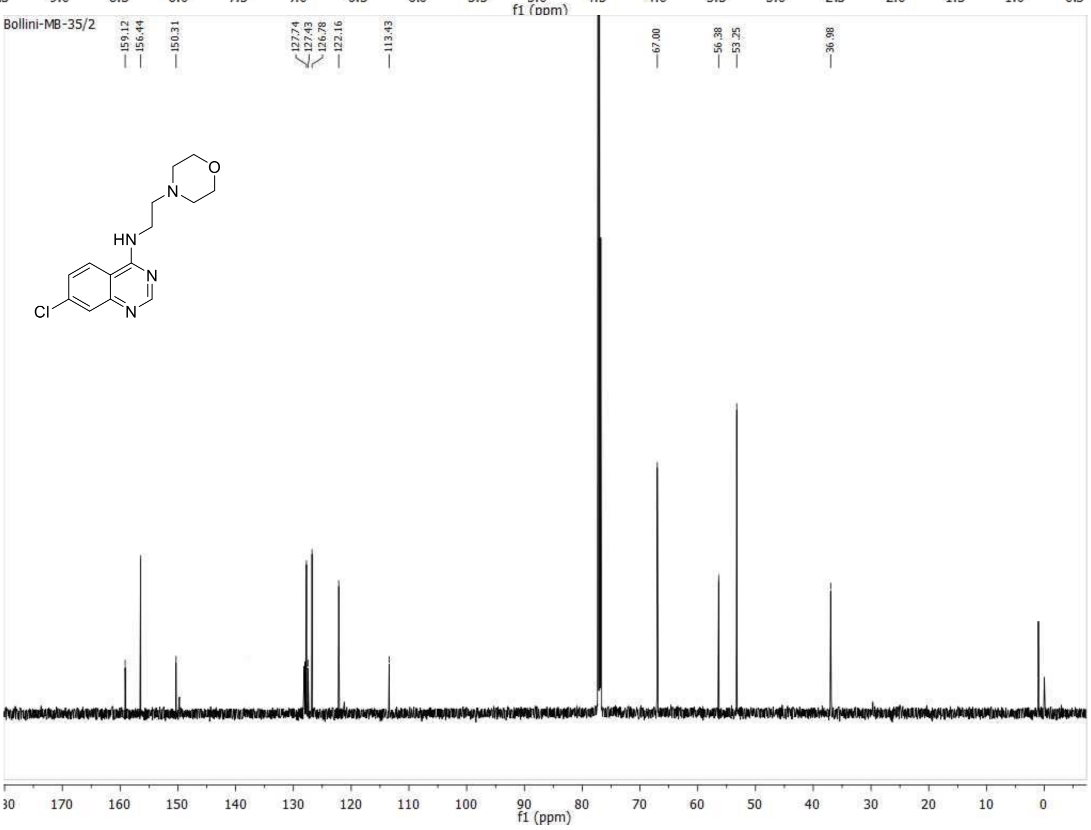

2-(4-(7-chloroquinazolin-4-yl)piperazin-1-yl)ethan-1-ol (**1.22**)

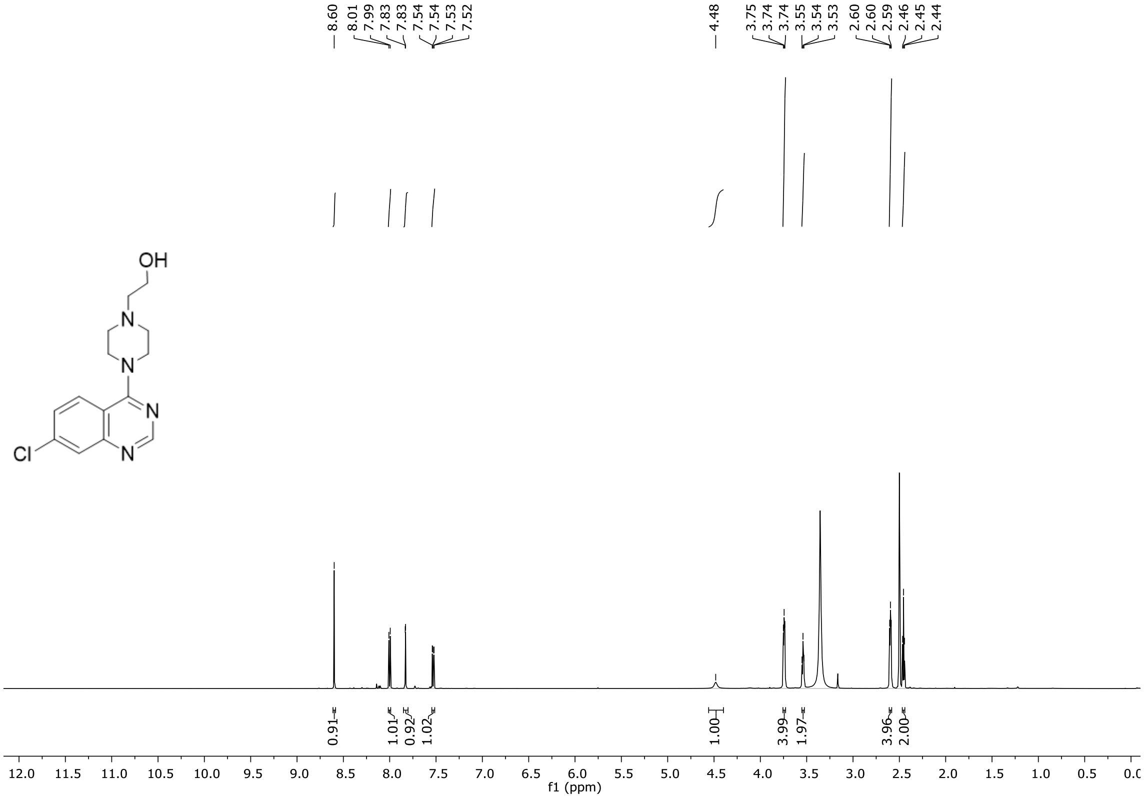

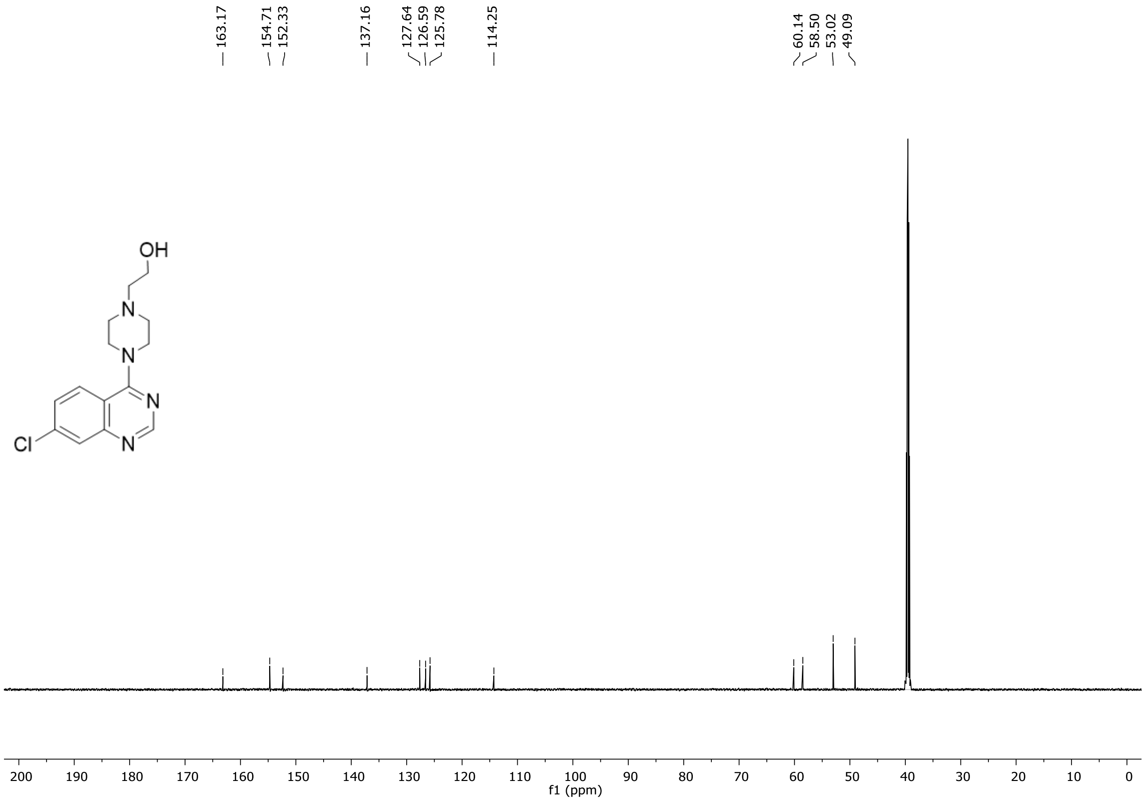

7-chloro-N-(2-(piperidin-1-yl)ethyl)quinazolin-4-amine (**1.23**)

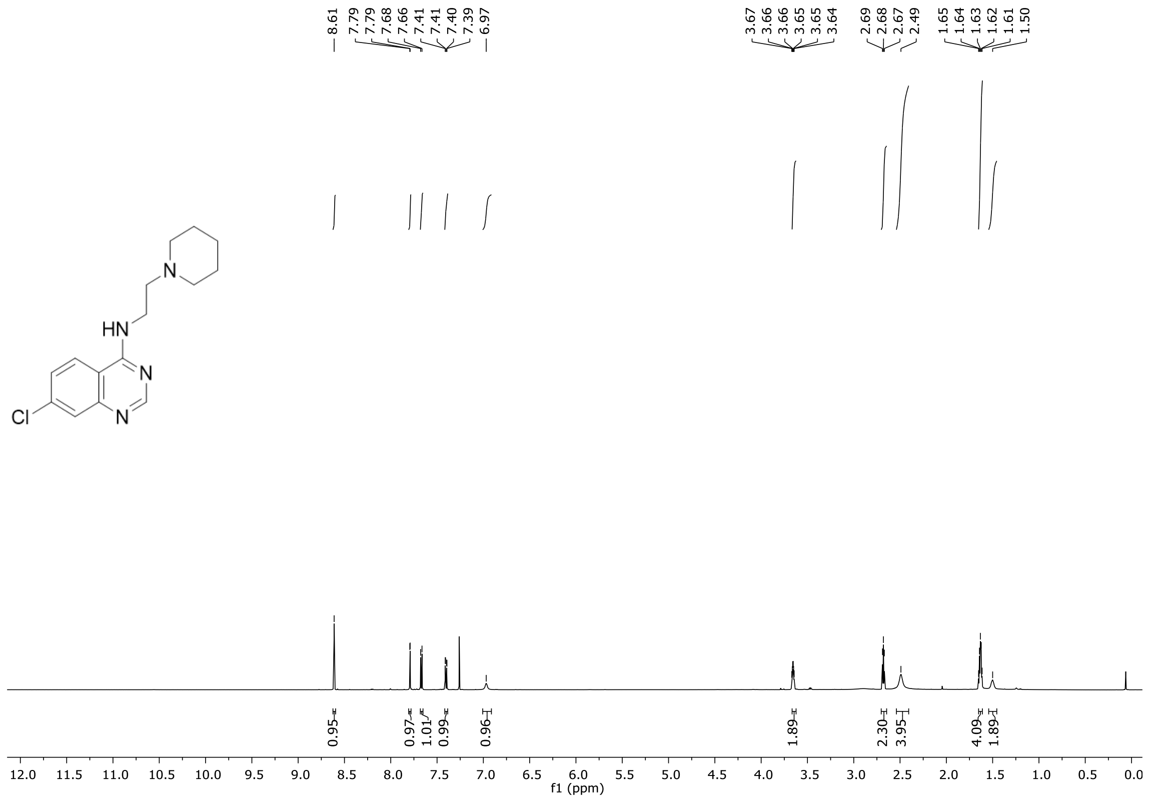

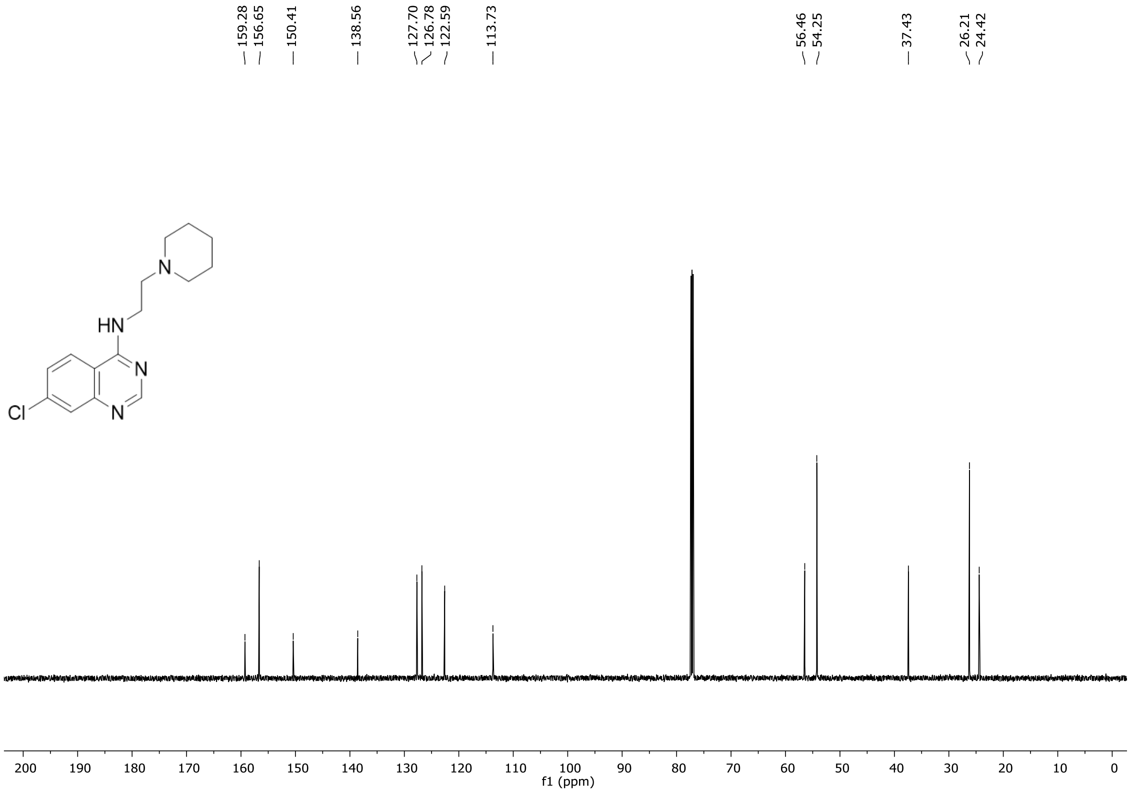

7-chloro-4-(4-methylpiperazin-1-yl)quinazoline (**1.24**)

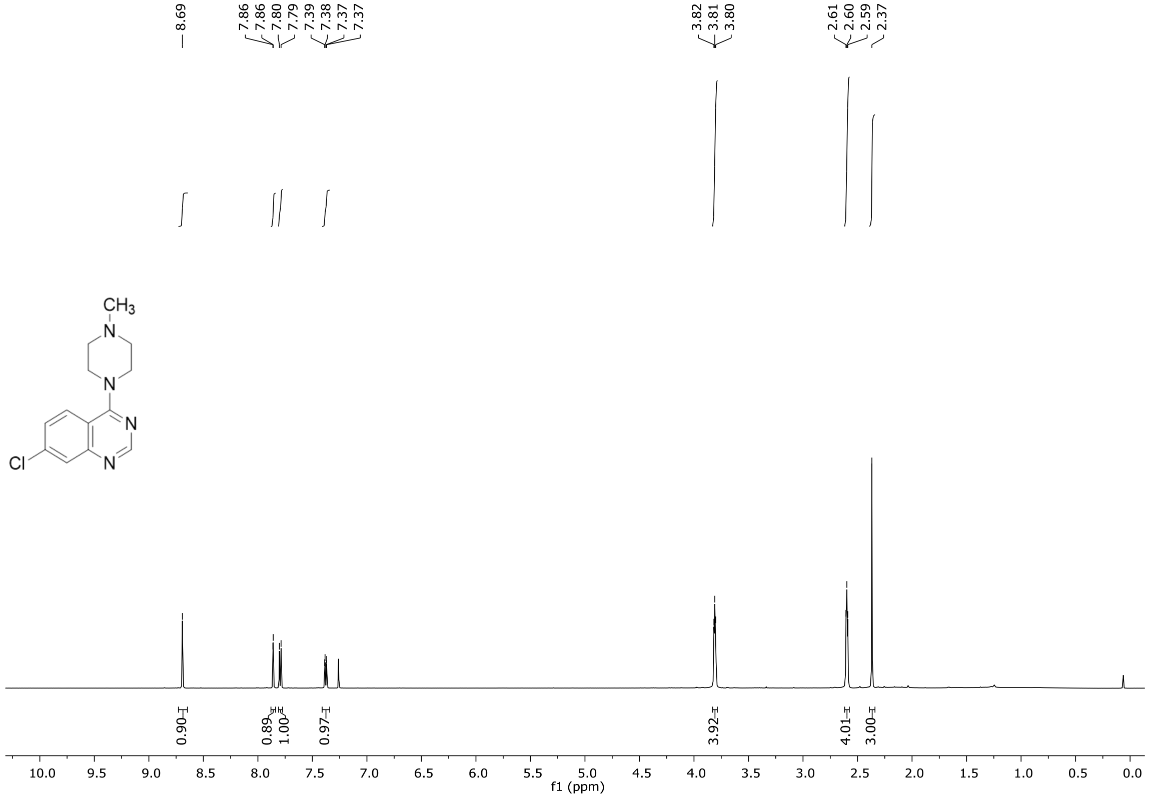

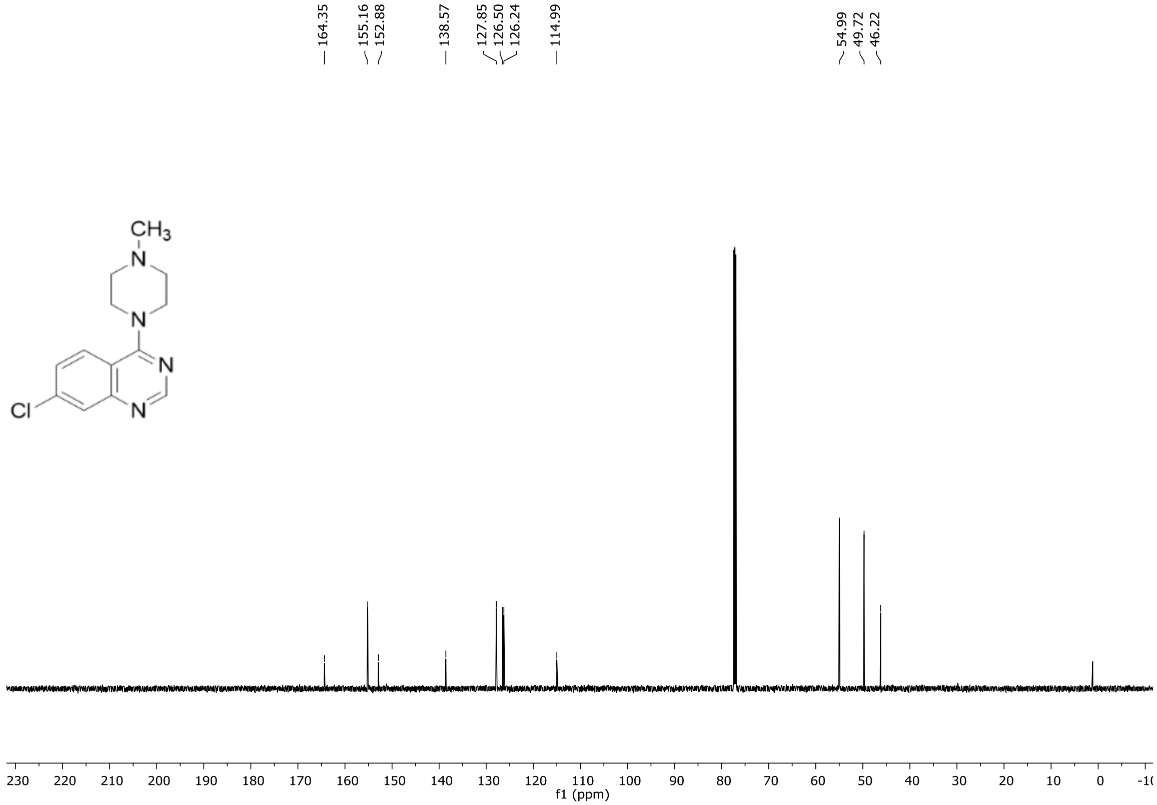

Supplement: Supplementary file 1 [file Data_Sheet_1.PDF]
